# Integrated Assessment of Artisanal and Small-Scale Gold Mining in Ghana—Part 2: Natural Sciences Review

**DOI:** 10.3390/ijerph120808971

**Published:** 2015-07-31

**Authors:** Mozhgon Rajaee, Samuel Obiri, Allyson Green, Rachel Long, Samuel J. Cobbina, Vincent Nartey, David Buck, Edward Antwi, Niladri Basu

**Affiliations:** 1 Department of Environmental Health Sciences, University of Michigan School of Public Health, Ann Arbor, MI 48109, USA; E-Mails: mrajae@umich.edu (M.R.); green.allyson@gmail.com (A.G.); rachlong@umich.edu (R.L.); 2 Council for Scientific and Industrial Research-Water Research Institute, Tamale, Ghana; E-Mail: obirisamuel@gmail.com; 3 Faculty of Renewable Natural Resources, University for Development Studies, Nyankpala, Ghana; E-Mail: cobbinasamuel@yahoo.com; 4 Department of Chemistry, University of Ghana, Legon, Ghana; E-Mail: vknartey@gmail.com; 5 Biodiversity Research Institute, Portland, ME 04103, USA; E-Mail: david.buck@briloon.org; 6 Centre for Energy, Environment & Sustainable Development, Kumasi, Ghana; E-Mail: oldsojagh@gmail.com; 7 Faculty of Agricultural and Environmental Sciences, McGill University, Montreal, QC H3A 0G4, Canada

**Keywords:** small-scale gold mining, Ghana integrated assessment, mercury, metals, water, public health, ecotoxicology

## Abstract

This paper is one of three synthesis documents produced via an integrated assessment (IA) that aims to increase understanding of artisanal and small-scale gold mining (ASGM) in Ghana. Given the complexities surrounding ASGM, an integrated assessment (IA) framework was utilized to analyze socio-economic, health, and environmental data, and co-develop evidence-based responses with stakeholders. This paper focuses on the causes, status, trends, and consequences of ecological issues related to ASGM activity in Ghana. It reviews dozens of studies and thousands of samples to document evidence of heavy metals contamination in ecological media across Ghana. Soil and water mercury concentrations were generally lower than guideline values, but sediment mercury concentrations surpassed guideline values in 64% of samples. Arsenic, cadmium, and lead exceeded guideline values in 67%, 17%, and 24% of water samples, respectively. Other water quality parameters near ASGM sites show impairment, with some samples exceeding guidelines for acidity, turbidity, and nitrates. Additional ASGM-related stressors on environmental quality and ecosystem services include deforestation, land degradation, biodiversity loss, legacy contamination, and potential linkages to climate change. Though more research is needed to further elucidate the long-term impacts of ASGM on the environment, the plausible consequences of ecological damages should guide policies and actions to address the unique challenges posed by ASGM.

## 1. Introduction

The practice of artisanal and small-scale gold mining (ASGM) is increasing in many low- and middle-income countries (LMICs), mainly due to the rising price of gold and widespread poverty. Gold from these informal mines may represent 20–30% of the world’s output [1]. It is estimated that about 15 million people work in ASGM and that perhaps 100 million people worldwide depend on the sector for their livelihood [2]. Gold has been mined in Ghana for over 1000 years [3], and in 2013 gold accounted for 34.4% of the country’s national export revenue [4]. The proportion of Ghana’s gold that is mined through ASGM has increased from 6% in 2000 to 23% in 2010 [5].

**Figure 1:**
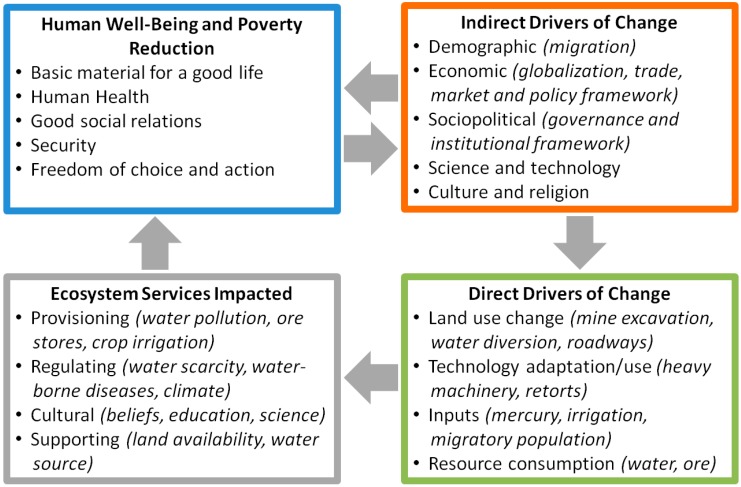
Framework linking key drivers and impacted natural systems. Principal domains of inquiry are highlighted. Framework is adapted from the Millennium Ecosystem Assessment [7].

Artisanal and small-scale gold mining, like other extractive activities, raises numerous environmental concerns. Emissions of mercury (Hg) into the atmosphere as well as direct releases of mercury to soil and water are of primary concern because of the extensive use of mercury to amalgamate gold by artisanal miners. Recent estimates suggest that the ASGM sector is the primary source of mercury into the global atmosphere, accounting for approximately 37% (727 tonnes) of all global emissions [6]. While mercury has gained most attention, there exist many other direct and indirect factors that contribute to poor ecological conditions in ASGM communities (Figure 1). This necessitates that impacts on the natural system, as well as planning for interventions be viewed under a broad ecosystem lens. 

### 1.1. Objective

This report is one of three papers [8,9] being co-published to provide a foundation for a special issue in the *International Journal of Environmental Research and Public Health* entitled “Integrated Assessment of Artisanal and Small-Scale Gold Mining in Ghana” (http://www.mdpi.com/journal/ijerph/special_issues/asgm). This integrated assessment (IA) is guided by an over-arching policy-relevant question: What are the causes, consequences, and correctives of artisanal and small-scale gold mining in Ghana? More specifically, what alternatives are available in resource-limited settings in Ghana that allow for artisanal and small-scale gold-mining to occur in a manner that is safe for ecological health and human health without affecting near- and long-term economic prosperity? Given the complex and global nature of ASGM, an integrated assessment provides the framework for us to analyze economic, social, and environmental data, and co-develop evidence-based solutions with pertinent stakeholders [10].

The purpose of this report is to document and scrutinize environmental impacts that may arise from ASGM activities in Ghana. The ultimate goal of the endeavor is to identify response and policy options associated with ASGM in Ghana that would lead to improved ecological health and sustainability. Ideally, the options would be sustainable, low-tech, health-promoting, and socially acceptable, while improving the standard of living of people who currently are involved in ASGM activities. As part of the IA, here we present evidence from Ghanaian ASGM sites that document relatively high levels of metals (e.g., mercury, cadmium, arsenic, and lead) in ecological media including soil, foodstuffs, sediment, and water. Impairment of water quality (e.g., acidity, turbidity, and nitrates) was noted at many sites based on “snapshot” samples by several studies. We also review limited data on deforestation, agriculture, biodiversity, desertification, and ecosystem services for people dependent upon altered lands. 

### 1.2. Limitations and Assumptions 

Substantial gaps in data availability, not only in Ghana but elsewhere, prevent a full assessment of ecological risks associated with ASGM. Public policy should be grounded in strong, objective, peer-reviewed science. Speculative conclusions and opinions about possible hazards based solely upon anecdotes and oversimplified chronologies are not a sufficient foundation to advance regulatory reforms or policies. Nevertheless, ecological concerns, especially those with scientific plausibility and those recurring across temporal and spatial scales, need to be taken seriously. In this report, all currently available evidence was reviewed and considered. As best as possible, all evidence from Ghana was reviewed (peer-reviewed and non-refereed; published and non-published; *etc*.), though studies from other regions were drawn in as appropriate.

A majority of the evidence reviewed in this report was obtained from studies undertaken in Ghana. However, within the country, there exists wide variation in the types of communities and ecosystems in which mines are situated (e.g., the south is more tropical, populated, and developed than the north of Ghana). While a majority of ecological issues (e.g., mercury contamination, land use degradation) are ubiquitous across sites, it is recognized that some risks may be site-specific owing to, for example, variation in types of receptors (e.g., organisms, vegetation) present. For the purposes of this assessment, we maintain broad generalizations as the focus is on developing countrywide response options. 

There is a low level of research on the effects of small-scale gold mining on the natural environment in Ghana. Many studies are based in areas with varied and overlapping activities (*i.e.*, large, small-scale, and illegal mining activities occurring simultaneously) making it difficult to specifically identify ASGM’s role in affecting ecological health. In addition, ASGM sites may cluster, making it difficult to isolate impacts associated with single sites. It is also near impossible to distinguish between legal and illegal ASGM mining operations, and thus determine if differences exist between the groups in terms of their impacts on the natural environment. In terms of operational methods, it is difficult to differentiate between the two groups of miners because they use similar methods for obtaining mineral-laden ore and for extracting the gold. 

This review is also limited by the available data in other studies. While a few studies provided full datasets to adequately summarize measures of central tendency, a number of studies lack information on the distribution and spread of metals concentrations, for example, or explicit information on the number of samples and subsamples collected. A few studies with inadequate information on data collection or low quality assurance and control were excluded from this review. 

The lack of coordination between research institutions, academia, and policy-makers; funding for issues of ASGM; the political will to implement policies; and legal and institutional frameworks concerning mining in Ghana all pose barriers to providing and exchanging information and implementing changes. 

## 2. An Assessment of the Ecological Health Issues 

**Figure 2:**
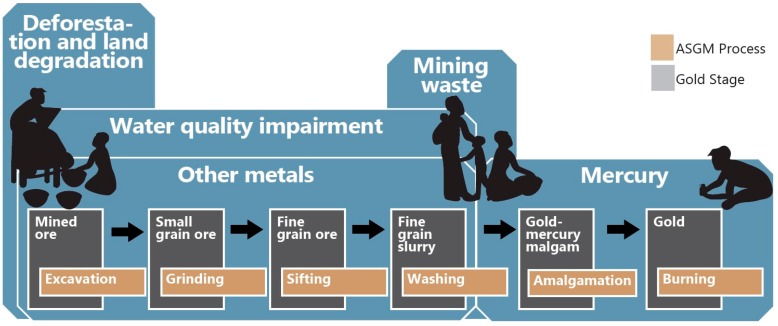
Key ecological hazards in the Ghanaian artisanal and small-scale gold mining (ASGM) sector. Silhouettes adapted from UNEP Mercury: Time to Act (2013) [11].

Here we review and analyze natural science issues that arise because of ASGM in Ghana by discussing the causes, status and trends, and consequences of key hazards. This assessment summarizes scientific knowledge to help build consensus and guide decision-making in the selection of response options. The information is intended to be an objective description of the current conditions. Here the key consequences of ASGM towards the health of natural systems are itemized and briefly described. These consequences serve as means to prioritize, combine, and summarize the most important points identified in the previous section. Figure 2 displays the causes and consequences highlighted in this report. 

### 2.1. Mercury Contamination

#### 2.1.1. Causes

Mercury is a naturally occurring metal that exists in three primary forms in nature: elemental (Hg^0^), inorganic mercurial salts (e.g., HgS, HgCl_2_, Hg^+^, Hg^2+^), and organic mercury (e.g., CH_3_Hg, or methylmercury). Elemental mercury is used in ASGM because of its ability to isolate gold from other non-target minerals. Mercury creates a bond with gold, called an amalgam. Because of the low vapor pressure of mercury, burning the gold-mercury amalgam leaves the valuable gold behind. Mercury emitted during amalgam burning can have significant impacts at the local scale in villages and towns where mercury vapor is emitted, and globally when that mercury vapor enters the global atmospheric pool and is transported large distances before being redeposited as inorganic mercury on the landscape [12]. Inorganic mercury can be methylated (bound to carbon) by microorganisms mainly in aquatic ecosystems. Methylmercury is often found in fish at higher concentrations since it is able to bioaccumulate and biomagnify in organisms [12,13,14,15]. ASGM with mercury can result in atmospheric emissions as well as direct releases to soil and water, accounting for an estimated 37% of total global anthropogenic mercury emissions annually [6]. It has now been estimated by the United National Environment Programme (UNEP) that ASGM has surpassed fossil fuel combustion as the largest contributor to global anthropogenic mercury in the atmosphere [6]. The largest regional consumers of mercury for ASGM are East and Southeast Asia, South America, and Sub-Saharan Africa [16].

The use of mercury was banned in Ghana between 1932 and 1989, but is now in use [17]. Registered ASGM operators and licensed traders can purchase and trade mercury legally through authorized dealers (Ghana Government 1989, PNDCL 217 S.96) [18], such as the Precious Minerals Marketing Corporation (PMMC) Ltd. [19]. Mercury use, however, appears to be greater than what is officially available, according to the Ghana Minerals Commission and the PMMC, suggesting a significant “black market” for mercury. Recent upsurges in the demand for mercury may be in response to market conditions, as both the rising price of gold and falling demand for diamonds has driven an increase in ASGM activity [8,19]. 

**Figure 3:**
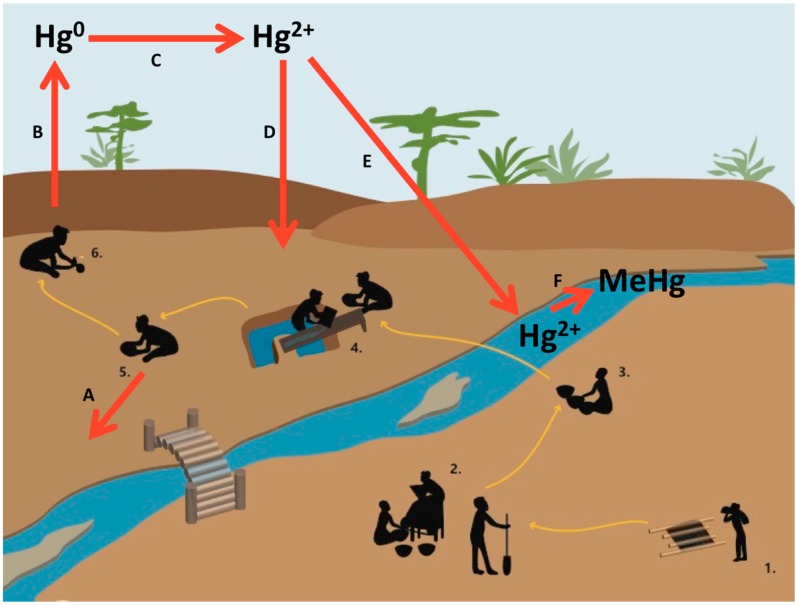
Mercury (Hg) cycle in a typical artisanal and small-scale gold mining (ASGM) process. Numbers represent key steps in the ASGM process: 1—excavation, 2—crushing and grinding, 3—sifting/shanking, 4—washing/sluicing, 5—amalgamation, and 6—burning. Letters represent key steps in the mercury cycle: A—residual mercury from amalgamation may be discarded in local soil and water, B—volatilization of elemental mercury into the atmosphere, C—oxidation of elemental mercury, D—deposition onto local terrestrial systems, E—deposition onto local aquatic systems, F—methylation of inorganic mercury to methylmercury.

Mercury is generally used in ASGM without any type of capture system to reduce chemical releases into the environment (Figure 3). Many miners have rejected retorts and complain of a slower process and an inability to see the gold [12,20]. Even transparent retorts, such as the ThermEx^®^ retort promoted by the Ghanaian government, have been underutilized because of their low capacity and fragility [20]. For example, a survey of 44 licensed and 77 unlicensed miners in the Denkyira corridor showed only 27% of respondents using a retort while 68% used open flame [21]. 

A life cycle analysis of ASGM in Peru by Valdivia and Ugaya [22] examined ASGM mining impacts and mercury use from gold ore. Using an alluvial mining case that most closely resembles ASGM in Ghana, they estimated that 2 kg of mercury is used to produce 1 kg of concentrated gold ore (99.5% gold) [22]. The authors of this paper visited two ASGM sites in Tarkwa, Ghana to estimate Hg use in the ASGM process. At one small-scale mine, after processing and concentration using sluices, approximately 210 grams of elemental mercury was added to the concentrate ore. Miners then combusted the amalgam ball, leaving behind sponge gold weighing 211.3 g. This material was then smelted using borax to remove any remaining impurities. Assuming impurities between 2–5%, the final amount of gold produced would be ~200g. 

**Figure 4:**
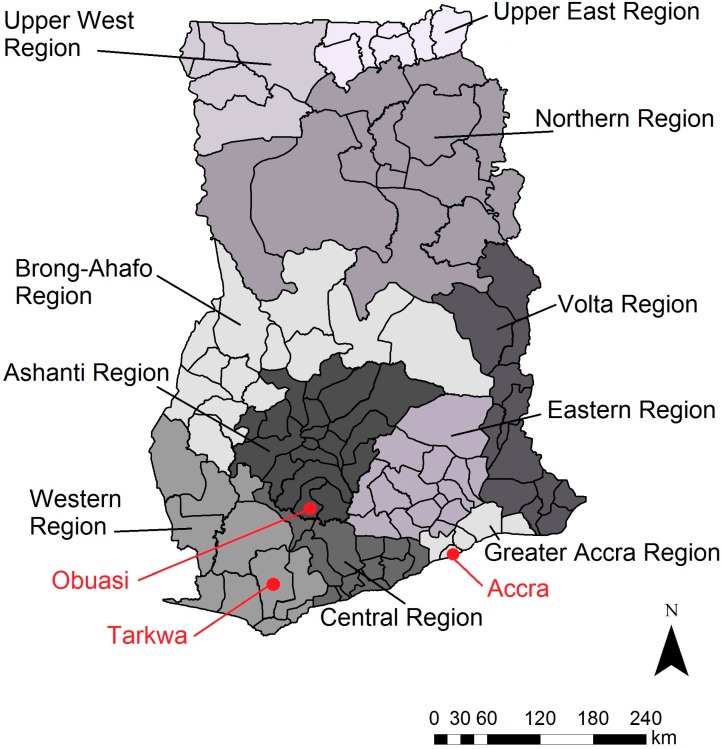
Regional map of Ghana. Key mining areas (Obuasi, Tarkwa) and the capital (Accra) are indicated.

#### 2.1.2. Status and Trends

Concern about mercury contamination in ecological media near ASGM sites in Ghana has prompted a number of studies assessing the extent of contamination. In total, 47 studies were found in nine regions of Ghana that documented mercury levels in soil, foodstuffs, sediment, water, tailings, and fish (Tables S1–S6, S19). Of the 47 studies, 20 sampled in the Western Region, 11 sampled in the Ashanti Region, nine sampled in the Central Region, and eight sampled in the Greater Accra Region. Only two studies sampled in the Eastern and Upper East Regions, respectively, and one study sampled in each the Northern, Volta, and Brong-Ahafo Regions. Research has focused in the southeast (Western, Ashanti, and Central Regions), where ASGM has historically been most common, but ASGM also occurs in the Upper East and Upper West Regions. Figure 4 displays a regional map of Ghana for reference. 

**Figure 5:**
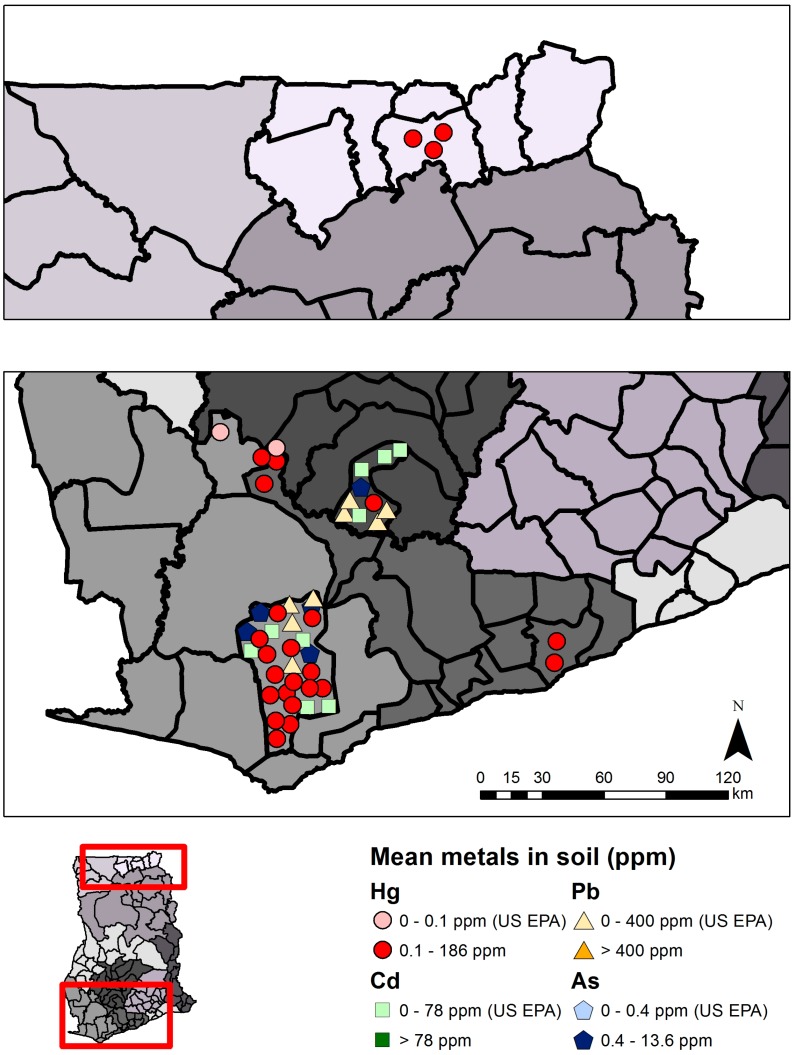
Metals in soil samples in southwest (bottom panel) and northeast (upper panel) Ghana. Each symbol represents the mean metals value (µg/g, ppm) from a single study in that region, and for illustrative convenience the symbols are scattered randomly within the district where the sampling took place. Political regions are distinguished by different shades of grey. Mean total mercury (Hg), lead (Pb), cadmium (Cd), or arsenic (As) above or below guidelines are indicated.

**Figure 6:**
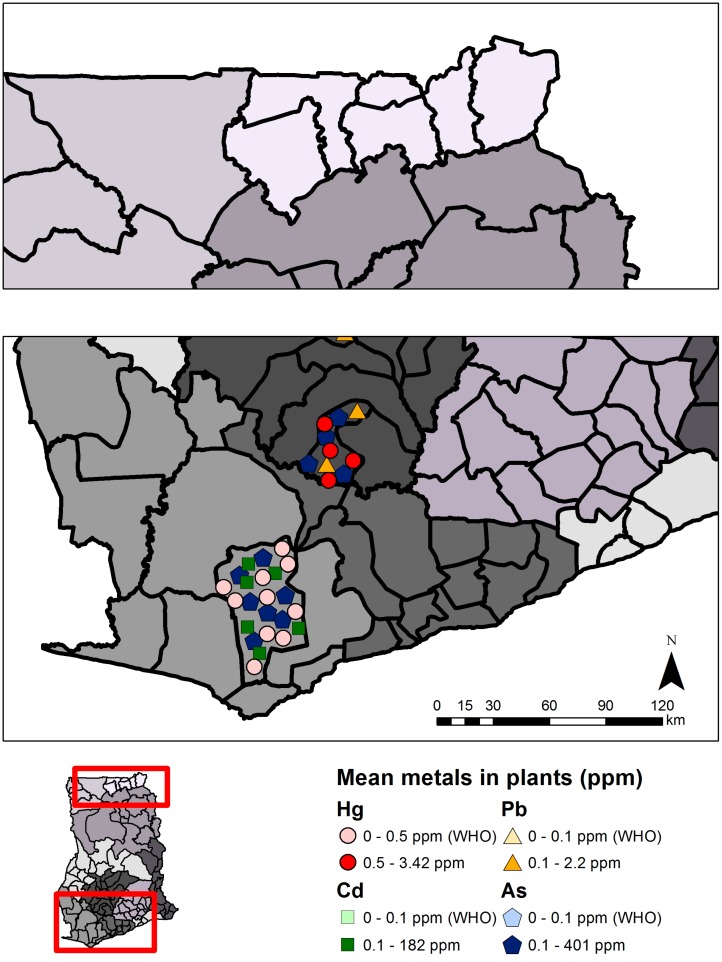
Metals in edible plant parts in southwest Ghana (bottom panel). Each symbol represents the mean metals value (µg/g or ppm) from a single study in that region, and for illustrative convenience the symbols are scattered randomly within the district where the sampling took place. Political regions are distinguished by different shades of grey. Mean total mercury (Hg), lead (Pb), cadmium (Cd), or arsenic (As) above or below guidelines are indicated. Plants sampled include cassava (*Manihot esculenta*), cocoyam (*Xanthosoma sagittifolium*), water cocoyam (*Colocasia esculenta*), plantain (*Musa paradisiacal*), and water fern (*Ceratopteris cornuta*) grown in or around current or former ASGM sites.

**Figure 7:**
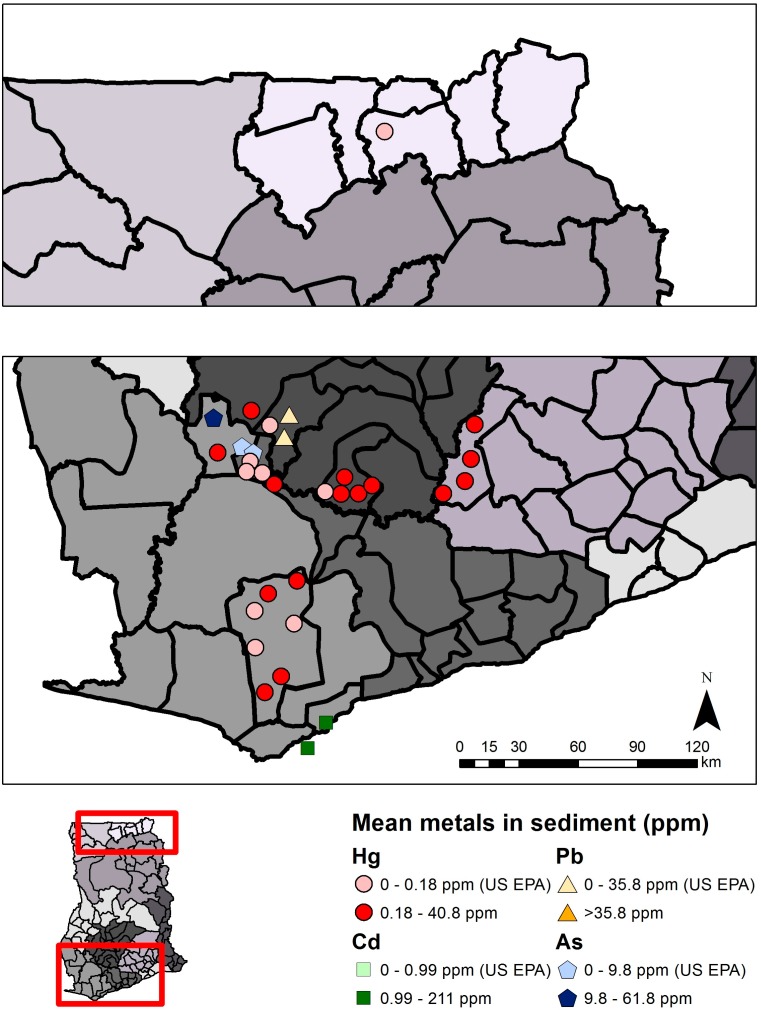
Metals in sediment samples in southwest (bottom panel) and northeast (upper panel) Ghana. Each symbol represents the mean metals value (µg/g or ppm) from a single study in that region, and for illustrative convenience the symbols are scattered randomly within the district where the sampling took place. Political regions are distinguished by different shades of grey. Mean total mercury (Hg), lead (Pb), cadmium (Cd), or arsenic (As) above or below guidelines are indicated.

**Figure 8:**
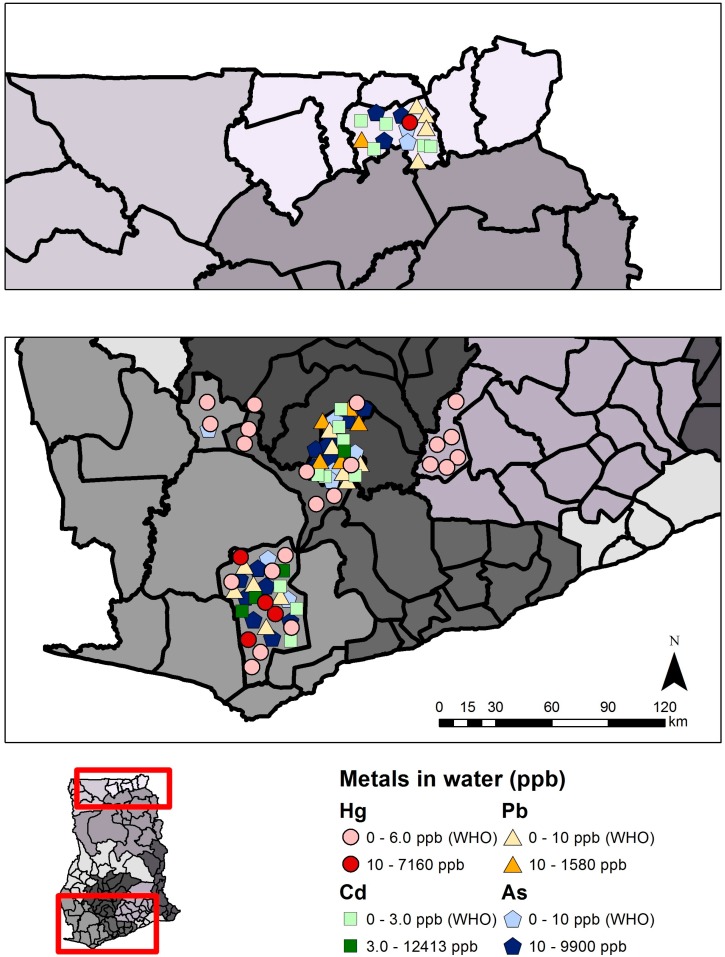
Metals in surface/groundwater samples in southwest (bottom panel) and northeast (upper panel) Ghana. Each symbol represents the mean metals value (µg/L or ppb) from a single study in that region, and for illustrative convenience the symbols are scattered randomly within the district where the sampling took place. Political regions are distinguished by different shades of grey. Mean total mercury (Hg), lead (Pb), cadmium (Cd), or arsenic (As) above or below guidelines are indicated.

**Figure 9:**
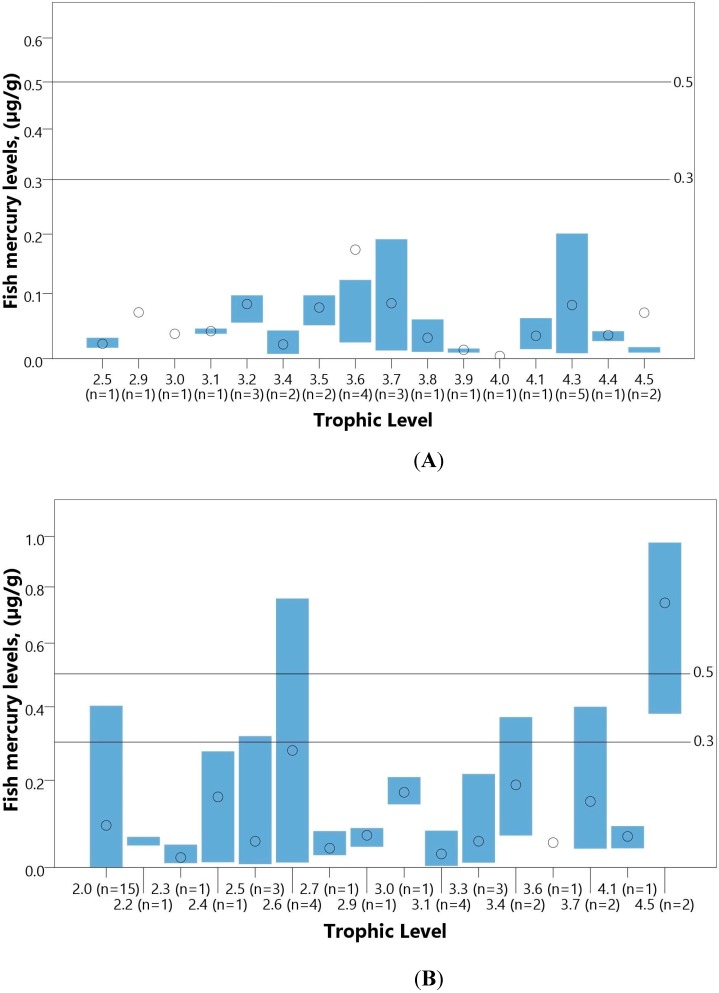
Mercury in marine (**A**) and freshwater (**B**) fish in Ghana. Bars represent range and dots represent means for fish at each trophic level (n = number of studies) across Ghana. For values below detection limit, standard protocol of dividing the detection limit by $\sqrt{2}$ was followed. Where minimum and maximum measurements were not available for a study, the mean value is included in the overall mean for the trophic level, but no minimum or maximum was included in calculations. WHO/FAO (0.5 µg/g) and U.S. EPA (0.3 µg/g) guideline values are indicated as horizontal lines [37,38]. All studies except for two sampled in districts or regions with ASGM, but only one explicitly sampled at or close to an ASGM site.

Below we provide a brief review of these studies with key results emphasized. International guideline values were used to evaluate mercury concentrations in various media when available, otherwise selected U.S. guidelines were used (Table 1). Figure 5, Figure 6, Figure 7, Figure 8 and Figure 9 show results synthesized from all relevant peer-reviewed data we could find. Table 2 summarizes the number of studies and samples reviewed for mercury and other heavy metals in various media, as well as reported mean concentration ranges. District boundaries have changed significantly in the past five years, but since boundary maps are not yet available and many studies reviewed refer to older districts, we have used the district names referred to in each respective study or older districts where geographic boundaries are available. Study sites referred to as “reference” sites in each respective study were designated as “non-mining” in this review. Since ASGM often occurs in areas with large-scale gold mining (LSGM), many sites reviewed include areas with ASGM and LSGM, and in areas near LSGM. These sites were designated as “mining” areas in this review. When detection limits were provided, a standard protocol of dividing the detection limit by $\sqrt{2}$ was followed for values below the detection limit. Values were reported as provided in each respective study when detection limits were not provided (*i.e.*, as zero or not detectable [ND]). 

Mercury concentrations in soil were reviewed in 11 studies including 727 samples (565 from ASGM and LSGM areas and 54 from non-mining areas) from the Western, Central, Ashanti, and Upper East Regions (Table 2, Figure 5). Mean total mercury concentration in soil (range across studies: not detectable–185.9 µg/g) were above the U.S. Environmental Protection Agency (U.S. EPA) Ecological Soil Screening level of 0.1 µg/g mercury in 88.0% of sampling sites reviewed at ASGM and LSGM sites (n = 25) [23,24]. Samples were generally below the Canadian Environmental Quality Guidelines (6.6 µg/g or residential soil; and 50 µg/g for industrial soil [25]). Some individual soil samples greatly exceeded guidelines (e.g., 185.9 µg/g Hg, abandoned Tarkwa mine site; 330.0 µg/g, ASGM community in the Upper East Region) [26,27] in certain cases. Mercury concentrations did not follow any obvious trend by season or temporally. Most sites in southern Ghana have mean mercury concentrations below those observed in northeast Ghana, with a couple exceptions in the Wassa West District near Tawkwa, Western Region [26]. Concentrations were highest from samples taken on-site of ASGM activities (n = 8 sites; mean range: 0.792 to 185.9 µg/g), followed by studies in ASGM and LSGM areas (n = 17 sites; mean range: 0.020 to 2.40 µg/g), and non-mining areas (n = 6 sites; mean range: not detectable to 0.170 µg/g). Samples from edible plants were highest from sites closest to ASGM and LSGM activities (Figure 6, Table S2). One out of six sites measuring mercury in cassava (n = 3 studies) and one out of two sites measuring mercury in plantains (n = 2 studies) were found to exceed the Food and Agriculture Organization (FAO) and the World Health Organization (WHO) guideline of 0.5 µg/g [28]. 

Mercury concentrations in sediment collected from rivers affected by mining activity more often exceeded guidelines than in non-mining areas. Fifteen studies were reviewed (140 samples from ASGM and LSGM areas and 53 from non-mining areas) from the Western, Central, Ashanti, Volta, Eastern, and Upper East Regions, with mean values ranging from not detectable to 40.8 µg/g Hg (Table S3). For example, 59.3% of sampling sites reviewed in ASGM areas (n = 10 studies) reported means above the U.S. EPA guideline of 0.18 µg/g Hg dry weight (d.w.) (Figure 7) [29]. Sediment mercury concentrations were generally higher in sites closer to ASGM activity. Mean mercury concentrations in non-mining areas (n = 5 studies) ranged from not detectable to 0.494 µg/g Hg [30,31]. There were no obvious temporal trends. Sediment mercury concentrations were often higher in the dry season compared to the wet season, with some exceptions. Only one study explored upstream and downstream samples in Birim North District, Eastern Region, and observed mercury concentrations that were at least double the concentration from downstream samples compared to upstream [32]. 

Surface and groundwater samples from rivers, streams, boreholes, and wells also show a range of mercury values (mean range: not detectable to 7160 µg/L Hg) (Table S4). Eighteen studies were reviewed across Ghana (615 samples from ASGM and LSGM areas and 112 from non-mining areas) in the Western, Central, Ashanti, Greater Accra, Eastern, Upper East, and Northern Regions. All but four studies analyzed unfiltered water samples. Most study means were below the WHO drinking water standard for inorganic mercury (6 µg/L, [33]); 23.3% of 30 sites sampled in mining areas exceeded 6 µg/L Hg (Figure 8). Groundwater mercury concentrations were generally lower than surface water concentrations. As with other ecological samples, mercury concentrations varied spatially with higher mercury concentrations found in districts with ASGM and LSGM. Seasonally, water mercury concentrations were higher in the dry season than the wet season with a few exceptions. 

Fifteen studies were reviewed on mercury in 65 species of freshwater and marine fish and shellfish (n = 1305 samples total, n = 15 studies) (Table S5). Nine studies sampled freshwater fish (31 species) and shellfish (1 species), and six studies sampled marine fish (28 species) and shellfish (5 species). Fish were assigned a trophic level value from FishBase [34], except for one noted study that indicated trophic levels [35]. All mercury concentrations are presented as wet weight concentrations; dry weight concentrations were converted to wet weight assuming an 80% moisture content when the moisture content was not listed [36]. Total mercury concentrations in fish and shellfish are presented in this review, since many studies lacked data on methylmercury concentrations. Although the U.S. EPA and FAO/WHO guidelines were developed specifically for methylmercury in fish (0.3 µg/g and 0.5 µg/g, respectively) [37,38], fish mercury is often >90% methylmercury [39]. Figure 9 displays the mean concentrations of total mercury in marine and freshwater fish by trophic levels. 

All of the freshwater fish studied were sampled in areas with ASGM except for two studies [36,40], but only one study specifically sampled fish directly from streams or reservoirs within ASGM sites or next to ASGM communities [27]. In freshwater fish and shellfish, mercury concentrations ranged from <0.001 µg/g wet weight (w.w.) in *Tilapia zilli*, *Tilapia multifasciata*, *Sarotherodon melanotheron*, and *Labeo coubie* species [35,36,40] to 0.975 µg/g w.w. in *Hepsetus odoe* [41]. Three of the four lowest samples were from areas without mining activities. In general, mean concentrations were generally below U.S. EPA and FAO/WHO guidelines (Figure 9). Mean concentrations of freshwater fish exceeded the FAO/WHO guideline of 0.5 µg/g in two sampling points [38], and approximately 7.7% (n = 4 of 52 total sampling sites and species) exceeded the U.S. EPA guideline of 0.3 µg/g Hg [37], specifically *Sarotherodon melanotheron*, *Tilapia zilli*, *Chrysichthys* species, *Synodontis* species, *Heterobranchus bidosalis*, and *Hepsetus odoe*. The study of fish from streams and reservoirs adjacent to ASGM communities in the Upper East Region observed low mercury concentrations in unspecified fish species (mean: 0.014 µg/g w.w., range: 0.005 to 0.044 µg/g Hg w.w.) [27]. Mean mercury concentrations were low for freshwater fish species in non-ASGM areas for *Sarotherodon melanotheron*, *Tilapia multifasciata*, and *Tilapia zilli*, but showed no differing trend between mining and non-mining areas in other species. 

Mercury concentrations in marine fish ranged from 0.004 µg/g w.w. in *Stromatteus flatola* [42] to 0.430 µg/g w.w. in *Decapterus rhonchus* [42]. Only the *Decapterus rhonchus* sampled by Voegborlo and Akagi exceeded the U.S. EPA guideline of 0.3 µg/g Hg, with the second highest mercury sampled in *Auxis thazard* thazard at 0.201 µg/g total mercury [43]. Mercury concentrations in marine shellfish ranged from 0.01 µg/g in *Anadara senilis* and *Crassostrea tulipa* [44,45] to 0.74 µg/g in *Perna perna* [45].

**Table 1:** Summary of guideline values for mercury (Hg), arsenic (As), cadmium (Cd), and lead (Pb) in soil, plants, sediment, water, fish, and tailings.

	Mercury (Hg)		Arsenic (As)		Cadmium (Cd)		Lead (Pb)	
Soil	0.1 µg/g THg	US EPA [23,24]	0.4 µg/g	US EPA [23]	78 µg/g	US EPA [23]	400 µg/g	US EPA [23]
Plants	0.5 µg/g MeHg	FAO/WHO [38]	0.1 µg/g	FAO/WHO [38]	0.1 µg/g	FAO/WHO [38]	0.1 µg/g	FAO/WHO [38]
Sediment	0.18 µg/g THg	US EPA [29]	9.8 µg/g	US EPA [29]	0.99 µg/g	US EPA [29]	35.8 µg/g	US EPA [29]
Water	6 µg/L Inorg. Hg	WHO [33]	10 µg/L	WHO, GSB/GWC [33,46]	3.0 µg/L	WHO, GSB/GWC [33,46]	10 µg/L	WHO, GSB/GWC [33,47,48]
Fish	0.3 µg/g MeHg	US EPA [37]						
	0.5 µg/g MeHg	FAO/WHO [38]						
Tailings	0.1 µg/g THg	US EPA [23,24]						

**Table 2:** Summaries of studies and samples reviewed for mercury (Hg), arsenic (As), cadmium (Cd), and lead (Pb), in soil, plants, sediment, water, fish, and tailings in all studies across Ghana, and those at or near ASGM and/or LSGM (mining) or in non-mining areas. Studies without mean values reported (e.g., only minimum and maximum) are not included in the min.–max. range in this table.

	Metal											
	Mercury (Hg)			Arsenic (As)			Cadmium (Cd)			Lead (Pb)		
	*n* studies	*n* samples	Mean min.–max.	*n* studies	*n* samples	Mean min.–max.	*n* studies	*n* samples	Mean min.–max.	*n* studies	*n* samples	Mean min.–max.
**Soil (µg/g)**												
Total	11	727	ND–185.9	5	549	0.1–227.0	3	430	0–3.958	2	422	0.042–11.0
Mining	10	565	0.020–185.9	3	295	1.1–227.0	2	271	10.0–3.958	2	266	0.042–5.80
Non-mining	5	54	ND–0.190	4	254	0.1–10.5	3	159	0–1.71	2	156	0.079–11.0
**Plants (µg/g)**												
Total	4	639	0.003–3.42	3	623	1.28–383.5	2	35	1.99–182	1	720	0.7–2.2
Mining	4	639	0.003–3.42	3	623	1.28–383.5	2	35	1.99–182	1	240	0.8–2.2
Non-mining	0	0	-	0	0	-	0	0	-	1	480	0.7–1.9
**Sediment (µg/g)**												
Total	15	193 *	ND–40.85	4	117 *	0.081–61.8	2	42	0.074–0.211	1	42	ND–0.243
Mining	13	140 *	ND–40.85	2	29 *	0.322–61.8	0	0	-	1	28	ND–0.243
Non-mining	5	53 *	ND–0.494	3	88	0.081–0.311	2	42	0.074–0.211	1	14	ND–0.105
**Water (µg/L)**												
Total	18	727 *	ND–7160	13	1053 *	0.017–10,100	9	575	<0.01–12,413	9	832	ND–1580.2
Mining	17	615 *	ND–7160	10	655 *	0.2–10,100	7	338	<0.01–12,413	8	626	ND–1580.2
Non-mining	5	112 *	ND–0.4	5	398	<0.017–1.93	4	237	<0.01–0.07	3	206	0.05–1.526
**Fish (µg/g)**												
Total	15	1305	0.004–0.430	-	-	-	-	-	-	-	-	-
**Tailings (µg/g)**												
Total	7	37 *	0.011–19.3	-	-	-	-	-	-	-	-	-

* Studies with unspecified sample sizes were assigned an n=1 for this summary, although the total sample size may be larger.

#### 2.1.3. Consequences

Health effects of mercury exposure on fish [49], wildlife [50], and human populations [51] have been documented worldwide. In fish and wildlife, relevant exposures have been shown in some cases to impair normal biological function in both individuals and populations with key systems impaired including the reproductive axis and nervous systems. In a recent review, Sandheinrich and Wiener [52] outline that sub-clinical changes such as gene expression alterations, oxidative stress, and effects upon reproductive hormones and behavior, occur in fish with muscle mercury concentrations ranging from 0.5 to 1.2 µg/g (w.w.) and in the whole body from 0.3 to 0.7 µg/g (w.w.). Most of the fish (individuals and species) studied in Ghana have levels below 0.3 µg/g, though a few instances of elevated exposures exist. Despite the potential for high mercury levels in Ghanaian fish, like in other parts of Africa the levels are lower than expected with reasons yet to be determined [53,54]. A review of mercury concentrations in fish in 12 countries in Sub-Saharan Africa found that only fish sampled near ASGM operations had mean mercury concentrations exceeding the FAO/WHO guideline [54]. For fish-eating wildlife, we are unaware of any studies in Ghana despite results from many other parts of the world showing these organisms to be sensitive to exposure [50]. 

#### 2.1.4. Certainty Evaluation

The numerous studies focusing on mercury contamination throughout Ghana provide a decent understanding of the state of mercury concentrations in various media. There is a high certainty (a number of scientific publications; plausibility; strength of association; consistency of findings) in the extent of mercury contamination in ecological media throughout Ghana. Mercury has been measured in many species of fish, foodstuffs, plants, sediment, soil, mining tailings, and water. Studies on mercury contamination, however, are concentrated in southwest Ghana. While this is the area with the most ASGM activity, mining occurs in other regions such as the Upper West and Upper East. Because studies have not been repeated at individual sites and have only occurred in the past twenty years, it is difficult to assess temporal and spatial trends. While no studies, to our knowledge, have linked mercury exposure with adverse effects in Ghanaian fish and wildlife, studies from other regions of the world give high certainty that real-world mercury levels are indeed harmful to the health of individuals and populations. However, when this understanding is extended to Ghana, at least for fish populations, it is possible that most have mercury levels below values associated with adverse outcomes.

### 2.2. Contamination from Other Heavy Metals 

#### 2.2.1. Causes

The land disruption that occurs in ASGM may play a role in increasing metals contamination. The excavation and processing of ore along with the disposal of tailings waste may facilitate the release of heavy metals into the environment that were otherwise sequestered. Some of these heavy metals can contaminate soils, sediments, and water sources and bioaccumulate in local biota [55]. 

#### 2.2.2. Status and Trends

Several studies have also focused on measuring arsenic (As), cadmium (Cd), copper (Cu), manganese (Mn), and lead (Pb) concentrations in soil, water, fish, biomarkers, sediment, and food crops such as cassava [56,57,58,59]. This review focuses on arsenic, cadmium, and lead, which were most widely studied in ecological media. Twenty-one studies were reviewed for arsenic, 14 for cadmium, and 13 for lead in soil, sediment, plants, and water (Table 2). Ghanaian and international guideline values were used to evaluate metals concentrations in various media when available, otherwise selected U.S. guidelines were used (Table 1). Figure 5, Figure 6, Figure 7 and Figure 8 summarize levels of selected metals in relation to guideline values. See Tables S7–S18 and S20–22 for complete review. District names are listed as described in the respective studies, although they may have been updated more recently. Sites were designated as non-mining (“reference”) or mining sites where ASGM and/or LSGM occur. 

Mean arsenic concentrations in soil, reviewed in five studies and 549 total samples, ranged from 0.1 to 227.0 µg/g (Figure 5, Table S7). The highest individual soil arsenic concentration was 48.9 µg/g, in Obuasi near both ASGM and LSGM activities [60]. All but one sampling site exceeded the U.S. EPA guideline value of 0.4 µg/g arsenic in soil [23]. Sample means were elevated at both mining and non-mining sites, although the highest concentrations were found in ASGM and LSGM areas. Mean cadmium in soil ranged from zero to 3.96 µg/g, from three studies and 430 samples (Figure 5, Table S11). Individual soil samples exceeded 50 µg/g cadmium in one study in the Wassa West District, Western Region [61], but all were below the US EPA guideline value (78 µg/g) [23]. Mean concentrations were highest in an ASGM and LSGM mining area (mean 3.96 µg/g) and non-mining area (mean = 1.71 µg/g) in the Wassa West District, Western Region [61], and showed no trend by mining activity. Lead was only reviewed in soil in two studies (422 samples total). Mean soil lead ranged from 0.042 to 11.0 µg/g (Figure 5, Table S15) and all sample site means were below the US EPA guideline value of 400 µg/g [23]. There was no obvious trend in lead concentrations by mining proximity. Not all of the studies, however, accurately reflect soil concentrations of cadmium, arsenic, or lead in solely ASGM areas, as most studies sampled from areas with both ASGM and LSGM. 

Arsenic concentrations were reviewed in three studies from ASGM, LSGM, and non-mining areas. Mean arsenic concentrations ranged from 1.28 µg/g in plantains (*Musa paradisiacal*) from Obuasi to 383.4 µg/g in water cocoyams (*Colocasia esculenta*) in Tarkwa [60,62]. Cadmium, reviewed in two studies, had mean plant concentrations that ranged from 1.76 µg/g in water cocoyam from the Wassa West District (sampled from mining and non-mining towns), to 182 µg/g in water cocoyam from Tarkwa [61,62]. Data were reported collectively for mining and non-mining areas of one study for arsenic and cadmium, and show no obvious trends by different types of mining areas. Lead was measured in oranges and avocadoes in one study by Golow and Laryea (1994) [63]. Lead concentrations were highest in avocado peels in the Obuasi mining area (2.2 µg/g lead), but varied for orange peels and flesh from Obuasi and non-mining areas in the Ashanti Region [63]. All edible plant samples were from mining sites and had mean concentrations of arsenic, cadmium, and lead above the WHO guidelines of 0.1 µg/g arsenic, cadmium, and lead in edible plants (Tables S20–S22) [38]. Regional summaries of arsenic, cadmium, and lead in plants are displayed in Figure 6, and reviewed in Tables S8, S12, and S16. 

Arsenic in sediment, reviewed in four studies, ranged from 0.081 to 61.8 µg/g (Table S9) [64,65,66,67]. Only one sampling site in an ASGM area exceeded the US EPA guideline of 9.8 µg/g arsenic in sediment [29]. One study observed higher arsenic concentrations in the dry season compared to the wet season along an ASGM area of the Pra River [65]. Two studies from non-mining areas measured cadmium in sediment (individual sample range: zero to 1.42 µg/g) (Table S13) [66,67]. Similarly, mean concentrations were all below the U.S. EPA guideline (0.99 µg/g) and were higher in the dry season [29,66]. Only one study examined lead in sediment, and mean concentrations were below U.S. EPA guideline values (35.8 µg/g) (Table S17) [29]. All samples measured in the dry season were below the detection limit, whereas samples measured in the wet season ranged from 0.012 to 2.441 µg/g [65]. Arsenic and lead concentrations were higher in mining areas compared to non-mining areas. Sediment concentrations of arsenic, cadmium, and lead are displayed in Figure 7. 

Arsenic was measured in water in 13 studies (n = 1053 total samples) (Table S10). Mean concentrations ranged from 0.017 µg/L in non-mining lagoons and estuaries [66] to 10,100 µg/L in a tailings dam at Obuasi [68]. Sixty-two percent of all sampling sites with ASGM and/or LSGM sampled (n = 29) exceeded the WHO and Ghana Standards Board/Ghana Water Company (GSB/GWC) standard of 10 µg/L (Table S20) [33,46,69]. No non-mining sites had mean arsenic concentrations that exceeded the WHO or GSB/GWC standards, but individual samples for surface and groundwater from Asante Akim Central District in the Ashanti Region (non-mining district) exceeded these standards (maximum of 6900 and 12,200 µg/L arsenic, respectively) [70]. Mean cadmium concentrations, reviewed in nine studies (n = 575 samples), ranged from <0.01 µg/L at a stream in communities around Obuasi [71] and a standpipe tap in Accra [57] to 12,413 µg/L from surface and tap water in Obuasi [72] (Table S14). Nineteen percent of all mining sampling sites (n = 21) reviewed had mean concentrations that exceeded the WHO and GSB/GWC standard of 3.0 µg/L cadmium (Table S21) [33,46,47]. All mean and individual sample concentrations were below the WHO and GSB/GWC standard for non-mining sampling sites. Cobbina *et al*. [56], for example, found elevated levels of cadmium and arsenic in a borehole at Kalin near Nangodi in the Nabdam District of the Upper East region, where mining is present. The mean concentrations of arsenic and cadmium were 348% and 1,108% higher than the recommended WHO permissible guideline value of 10 µg/L and 3.0 µg/L, respectively, for arsenic and cadmium [56]. Lead water concentrations were reviewed in eight studies (n = 832 total samples), and had mean values ranging from below the detection limit at a water treatment plant at Obuasi [73] to 1580.2 µg/L in surface and tap water at Obuasi [72] (Table S18). Six of the sampling sites with ASGM and/or LSGM (n = 28 total), or 21.4%, exceeded both the WHO and GSB/GWB standard of 10 µg/L lead (Table S22) [33,47,48]. None of the non-mining sampling sites had mean concentrations above the guideline values for lead in water. Water concentrations of arsenic, cadmium, and lead across Ghana are summarized in Figure 8. 

#### 2.2.3. Consequences

Metals contamination of flora, wildlife, aquatic life, and food crops may affect the health of these organisms. There is strong evidence that heavy metal-contaminated water, food, and soils can also impact human health [55,74]. The death of 463 children from lead poisoning in 2010 in an ASGM community in northwestern Nigeria, Zamfara, has highlighted the importance of heavy metals contamination in ASGM communities [75]. 

The source of these metals varies by location, and contamination is not always attributable to ASGM. Arsenic and cadmium are naturally occurring metals, but they are also associated with gold-bearing ore and are often found at elevated concentrations near gold mining sites [48,61,72]. Cadmium is also a by-product of smelting lead and zinc ores [48,72]. In the Tarkwa area, elevated cadmium concentrations may be the result of mining and processing of zinc and chalcophilic metals [48]. Some geologic formations, such as the Birimain and Tarkwanian rock systems found in the mining area of Tarkwa, contain high concentrations of lead, as well as cobalt and chromium [48]. Lead also derives from industrial discharges or mine drainage [48,72]. The weathering of ore tailings can lead to the leaching of heavy metals into other media such as water, soil, and sediment [56]. Proximity to gold mining was associated with arsenic and lead soil contamination [76]. Irrigated crops had higher arsenic concentrations when contaminated water was used for irrigation [76]. Another study found no association with arsenic, cadmium, or lead concentrations in soil and proximity to mining activities [72]. A study in Tarkwa and Accra observed no significant differences between urinary arsenic in people from Tarkwa and Accra, while arsenic in water remained low in both locations, suggesting other exposure routes to arsenic such as from contaminated food [57]. Our review showed highly elevated arsenic contamination in edible plants, supporting this additional exposure route concern. 

Arsenic, cadmium, and lead are all well-known toxic metals. Most research has focused on human health effects of these metals, but there are known impacts to organisms, wildlife, and ecosystem services. In arsenic-contaminated soils in Chhattisgarh, India, total biomass of microbials and fungi, and enzymatic activity were significantly reduced by all forms of arsenic [77]. While most soil samples reviewed in Ghana had mean concentrations below those observed in Chhattisgarh, close to one-third of sites have relevant arsenic concentrations. Acute toxicity tests observed that arsenic, cadmium, and lead had adverse effects on soybean root and shoot growth, although these concentrations are relevant to only about half of the Ghanaian sites sampled for arsenic and cadmium in soil [78]. Cadmium is able to accumulate in tissues of aquatic organisms and birds, where it can exert its toxicity [79]. Soil cadmium contamination may affect animal and human health particularly since it has significant soil-solution, soil-plant, and soil-invertebrate relationships that impact soil properties [80]. 

Arsenic and cadmium are associated with increased cancer risk and classified as carcinogenic to humans (Group 1; sufficient evidence of carcinogenicity in humans) by the International Agency for Research on Cancer [81]. Exposure to arsenic is associated with significant adverse effects on neurodevelopment and behavioral disorders, cardiovascular and respiratory diseases, skin lesions, and anaemia in pregnancy [82,83]. In Ghana, many of the buruli ulcer endemic communities are located in mining zones such as Amansie West, Wassa Amenfi, Dunkwa, and Tarkwa Nsuaem. Proximity to drainage channels and farmland with arsenic levels over 15 ppm, as well as distance to gold-mining sites, have been associated with a higher prevalence of buruli ulcer in these communities [84,85]. Cadmium ingestion is associated with kidney and skeletal toxicity (itai-itai disease), and cardiovascular disease [48]. In an historic ASGM town in the Upper East Region, Nangodi, the non-cancerous health risk for exposure to arsenic and cadmium in water exceeded the US EPA’s acceptable risk based on the potential development of keratosis, skin hyper-pigmentation, tremors, low IQ, and renal failure [56]. Childhood lead exposures are associated with reductions in grey matter in the brain in adults, particularly in the prefrontal cortex and anterior cingulate cortex, which are responsible for executive functions, decision-making, and mood regulation [86]. 

#### 2.2.4. Certainty Evaluation

There is moderate certainty (some scientific publications; plausibility, strength of association, consistency of findings) that ASGM activities across Ghana are releasing high, and potentially dangerous, levels of toxic metals such as arsenic and lead. A number of steps in the mining process (e.g., excavating and crushing ore) facilitate the release of other potentially toxic metals into the environment. Despite the potential for widespread contamination by toxic elements other than mercury, there is much less empirical evidence for such contamination in ASGM communities. A limited number of studies are now documenting contamination by metals in a number of sites in Ghana (e.g., contamination of water and food with relatively high levels of arsenic, cadmium, lead). Because studies on non-mercury heavy metals in ecological samples have so far been focused in southwest Ghana, the extent of pollution in northern Ghanaian mining areas, where mining activities are said to be expanding [87], is not well documented.

### 2.3. Water Quality

#### 2.3.1. Causes

Water conservation and protection policies are important to the ASGM sector and to those affected by it. Licensed miners with mineral rights may “for purposes of or ancillary to the mineral operations, obtain, divert, impound, convey and use water from a river, stream, underground reservoir or watercourse within the land the subject of the mineral right” (Ghana Minerals and Mining Act, Ghana Government 2006, Sec. 17) [88]. The National Water Policy of Ghana seeks to balance the demands of mining and community needs, to ensure its availability for hydropower generation, mining operations, industrial operations, transport, recreation, and to protect water sources in mining and other industrial areas. Established industries, like large scale mines, are required to develop and implement environmental management systems that account for their impact on water resources, including water use permits, effluent discharge permits, and efficient water use practices. ASGM practices, however, are not specified in the National Water Policy [89].

ASGM sites require water for a variety of functions (e.g., sluicing/washing, panning, and amalgamation preparation), and thus mining activities need to be located in close proximity to water sources. A life cycle analysis of ASGM in Peru by Valdivia and Ugaya [22] that examined ASGM mining impacts, found that alluvial mining required 49,019,000 L of water in the sluicing/washing step to yield one kilogram of concentrated gold ore (99.5% gold) from 23,922 tonnes of ore, or 2049 L of water for 1 tonne of ore processed. While estimates for ASGM water use in Ghana do not exist, it is reasonable to expect that the numbers are similar. This is an important area for future research.

In addition to the quantity of water used, ASGM activities can have significant impacts on water quality. Ore processing may result in intentional and unintentional releases of produced water and chemicals, cause erosion from surface disturbances, and alter water flow due to excessive surface or groundwater withdrawals. Localized lowered water tables, increased siltation in rivers, and increased flooding have all been observed with mining generally [90]. Owing to the transitional nature and short histories of some ASGM communities, potable water and sanitation infrastructure such as indoor plumbing and pit latrines, or even designated areas for defecation, may be non-existent, causing additional biological contamination concerns for drinking water sources [91]. 

#### 2.3.2. Status and Trends

While studies have assessed heavy metals contamination in water, few have examined water quality parameters. It is difficult to assess the impacts of ASGM on water quality alone, as ASGM and LSGM activities often occur in close proximity. A study in the Tarkwa area of southwest Ghana, where large and small-scale gold mining occur along with subsistence farming, found median groundwater and surface water pH (5.71, 5.05, and 6.40 for borehole, well, and stream water, respectively) below the WHO guideline range (6.5–8.5) [57]. One tap water sample from Accra had a pH of 7.50, well within the WHO guideline range [57]. Another study in Tarkwa measured 26 groundwater water samples and found that the median pH (6.39) was just below the WHO guideline and that 54% of samples did not comply with the WHO guideline range [92]. A study of water samples from boreholes and other improved water sources from across Ghana (n = 199) found a median pH of 6.43, with 53% of samples below the WHO guideline [93]. Samples with particularly low pH were in mining areas (large and small-scale) and in more heavily forested areas, which are expected to be more acidic [93]. These studies, along with others [47,48], indicate slight water acidity in relation to ASGM and large-scale mining activities, compared to sites without any mining activities. 

The aforementioned Armah *et al*. study in Tarkwa measured a number of other water quality parameters besides pH, including electrical conductivity, total dissolved solids, turbidity, nitrates, sulfates, and chemical oxygen demand. In this study, all samples were above the WHO guidelines for turbidity (5 Nephelometric Turbidity Units; NTU), with a median of 27.5 NTU. Eighty percent of all samples did not comply with the WHO guideline for chemical oxygen demand (20 mg/L), with a median of 39.0 mg/L. The median electrical conductivity, total dissolved solids, nitrates, and sulfates parameters were within WHO guideline values. The groundwater samples were also rated by a Water Quality Index (WQI; weighted ratings of the seven water quality parameters), and found that all samples were severely contaminated and unfit for human consumption and that the mean WQI was eight times greater than the limit for consumption [92]. Rossiter *et al*.’s study of drinking water sources across Ghana assessed 199 samples on conductivity, turbidity, nitrates, sulfates, uranium, and various heavy metals. Water samples were filtered before analyses on nitrates, sulfates, uranium, and heavy metals. Ninety percent (median = 0.79 NTU) and 21% (median = 6.39 mg/L) of samples exceeded the WHO guidelines values for turbidity and nitrates, respectively. Turbidity in this study, however, was measured to a WHO guideline for effective disinfection of 0.1 NTUs, far lower than the WHO drinking water guideline of 5 NTUs used in Armah *et al*.’s Tarkwa study. High turbidity in mining areas is an indication of land disturbances and can also decrease drinking water disinfection efficacy. Particularly in rural areas, high turbidity may lead to higher rates of gastrointestinal diseases since many people consume unfiltered or untreated surface and groundwater [91]. 

#### 2.3.3. Consequences

Mining activities and removal of vegetation for mining can cause siltation and sedimentation problems, and increase run off, affecting turbidity [94]. Pollution runoff can alter dissolved oxygen, pH, turbidity, conductivity, and other parameters in water bodies. The impact of pollution to water bodies in some mining communities in Ghana have resulted in water stress and the need for new drinking water sources [95]. Impacts to receiving water streams and their biota are likely linked to the density of ASGM activities, the number and population of surrounding communities, the rate of installations, the distance of mining activities to stream channels, and a combination of roads, agricultural pasture, and other industrial operations. Water removal can more greatly affect small streams, where decreased water flow from surface or groundwater withdrawals may lower the dilution rate of solid loadings or contaminants in the watershed. This increased concentration of silt or contaminants can adversely affect aquatic ecology. Increased siltation can cloud water and decrease photosynthetic activity, making aquatic habitats less hospitable for biota [96]. Additionally, chemicals and nutrients can accumulate in waters and biota of aquatic systems impacted by siltation as they tend to sorb to sediments. 

**Figure 10:**
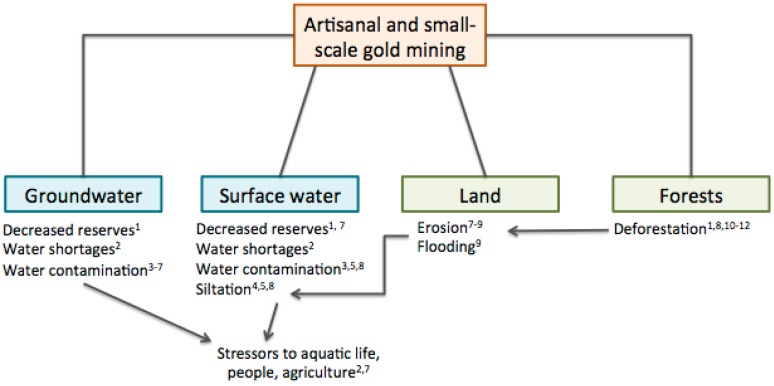
ASGM impacts on water systems. (^1^ Valdivia and Ugaya 2011 [22]; ^2^ Garvin *et al.* 2009 [95]; ^3^ Asante *et al*. 2007 [57]; ^4^ Armah *et al*. 2012 [92]; ^5^ Long *et al*. 2013 [91]; ^6^ Rossiter *et al*. 2010 [93]; ^7^ Burton and Johnston 2010 [96]; ^8^ Aryee *et al*. 2003 [94]; ^9^ United Nations Environment Programme (UNEP) 2008 [90]; ^10^ Kusimi 2008 [69]; ^11^ Schueler *et al*. 2011 [98]; ^12^ Tom-Dery *et al*. 2012 [99]).

Excessive surface and groundwater removal may also result in periods of water shortage that would impact drinking water access, agricultural irrigation, and aquatic life. Decreased surface and water quality is important, as it may be a major source of drinking water for ASGM communities [91]. Abandoned mine pits can fill with water, potentially providing breeding grounds for mosquitoes, and may serve as a source of water for mining activities and cooking purposes [91,94,97], posing an additional hazard to people living in ASGM communities, as reviewed in the Human Health review in this series [9]. Figure 10 summarizes ecological impacts on aquatic systems from ASGM.

#### 2.3.4. Certainty Evaluation

There is moderate certainty (some scientific publications; plausibility, strength of association) that ASGM activities are affecting water quality, although the majority of studies are from the southwest. Temporal data is lacking at individual sites to determine seasonal and natural variability in water quality, and the short- and long-term impacts of mining. Similarly, while there has been a focus on metals analysis in water and sediment, little attention has been paid to other water quality parameters at ASGM sites. It is also difficult to parcel out the impacts of large-scale mining (and other activities, such as farming) on water quality from those of ASGM, as these activities often occur in the same areas where studies have been conducted. Water withdrawal is a concern, but specific information on surface and groundwater recovery times, critical base flows for headwater streams, and impacts to aquatic life are largely unknown. 

### 2.4. Land Disturbances 

#### 2.4.1. Causes

Funding systems and policies are sometimes not in place to ensure that ASGM operations are done with environmentally sustainable practices nor to encourage environmental remediation. Because geologic explorations of sites are often not performed before small-scale mines are registered, miners may use a “trial and error” practice to locate gold deposits, which can increase environmental degradation [94]. This, despite the Minerals and Mining Act [88] stating that holders of a prospecting license shall “fill back or otherwise make safe to the satisfaction of the Commission a borehole or excavation made during the course of prospecting operations” and “remove within sixty days from the date of the expiration of the prospecting license a camp, temporary building or machinery erected or installed and make good to the satisfaction of the Commission damage to the surface of the ground occasioned by the removal” (Sec. 37.2). It can be difficult for ASG miners to access credit or funding to support sustainable mining practices, such as exploration, reclamation, or tailings disposal or processing [8,21,100]. 

**Figure 11:**
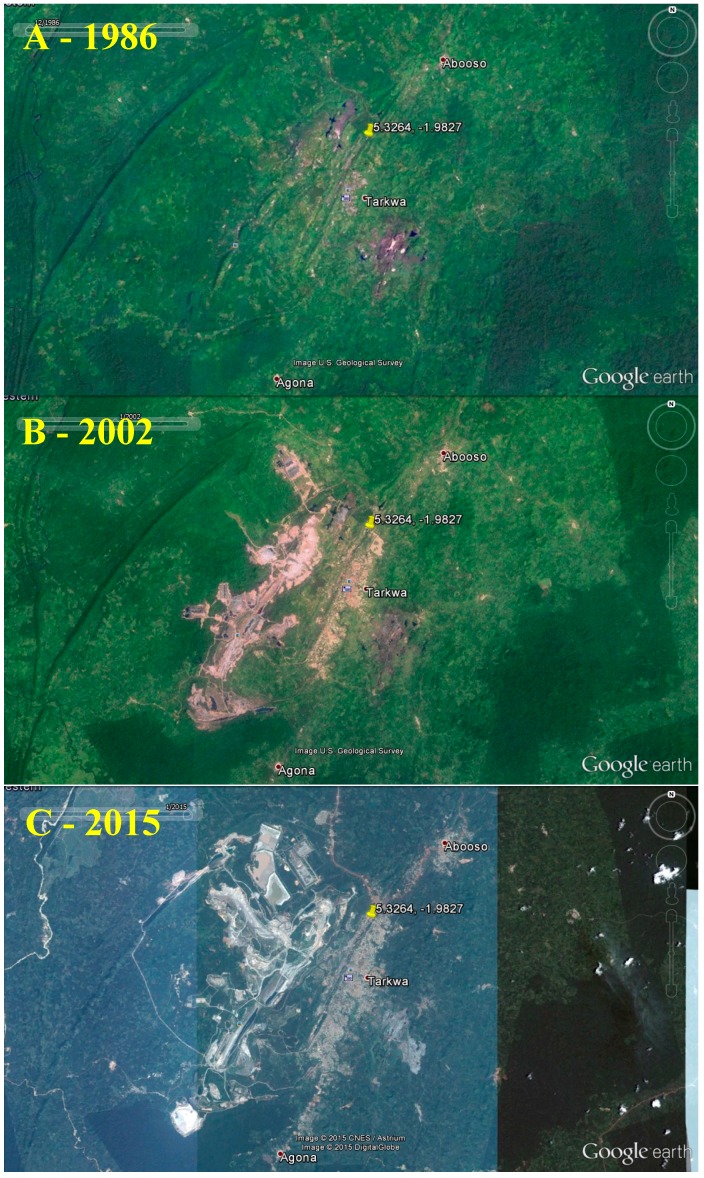
Satellite photos of Tarkwa, Western Region, from December 1986 (**A**), January 2002 (**B**), and January 2015 (**C**), depicting rapid gold mining development. Images from Google Earth (2015).

ASGM necessitates land disturbance and often requires the clearing of vegetation and forests [22]. Land disturbances (in ASGM, building construction, agriculture, *etc*.) increase the likelihood of soil erosion and sediment loading of streams. Landscape grades may need to be altered to accommodate ASGM activities. Access roads for transportation of materials and equipment may need to be established or constructed. Exposed surfaces increase run off of pollution and sediment to nearby streams, rivers, and lakes, if erosion controls are not enacted [96]. 

Terrestrial disturbances occur with all forms of ASGM though they perhaps are more common in illegal mining operations as there are fewer checks from authority. The legal consequences and violation fees for illegal mining are often not enough to deter unsustainable practices [94]. Legalization can provide oversight through permitting and increase monitoring of mining activities to potentially reduce negative environmental actions, although funding mechanisms remain an issue. 

#### 2.4.2. Status and Trends

Land degradation and deforestation is a substantial problem facing Ghana. Mining, as well as agriculture, population growth, and other factors, have contributed to widespread deforestation. Since 1940, there has been a 90% reduction of Ghana’s primary rain forest [101]). From 1990 to 2010, 34% of forest cover has been lost, decreasing from 32.7% to 21.7% nationally [102]. Forest cover was estimated to be 82,000 km^2^ in 1900, but by the late 1980s was reduced to only 18,000 km^2^, a loss of 78% of national forest cover [69]. Soil erosion and desertification is an additional problem threatening about one-third of land area [90]. 

The liberalization of gold mining in particular brought an influx of foreign investments and increased gold production. Mining was permitted and supported in forest reserves through guidance from the International Monetary Fund (IMF) and World Bank in the 1980s [90], and mining leases in forest reserves were allowed in 2003 [69]. Much of the forest loss due to mining comes from large-scale mines, but small-scale mines can also have detrimental impacts and often occur in tandem with large-scale mines, such as in Tarkwa, in the Wassa West District (Figure 11) [90].

In the Wassa West District of the Western Region, which is a major hub of all forms of gold mining discussed here (large-scale, registered small-scale, and illegal small-scale), remote sensing Landsat data from 1986 and 2002 has shown a decrease in natural land cover. During this same period there has been a 269.7% increase mining, farming, and open field land use. Biodiversity was also lost as primary forest cover degraded to secondary forest, and illegal mining activities, farming, and growth of urban areas contributed to a reduction in secondary forest [69]. Another study in the Western Region found that mining activities contributed to 58% of the deforestation and the loss of 45% of all farmland in the concession areas from 1986 to 2002; however, changes attributable to small-scale concessions and illegal activity in the area were outside the scope of the project [98]. Tom-Dery *et al*. [99] recorded low tree and shrub densities in mining communities in the Nangodi area in the Upper East Region. Along with deforestation from direct mining, communities built up around new mining sites may drive wood extraction and destruction of native vegetation for increasing agricultural, cooking fuel, and construction demands.

**Figure 12:**
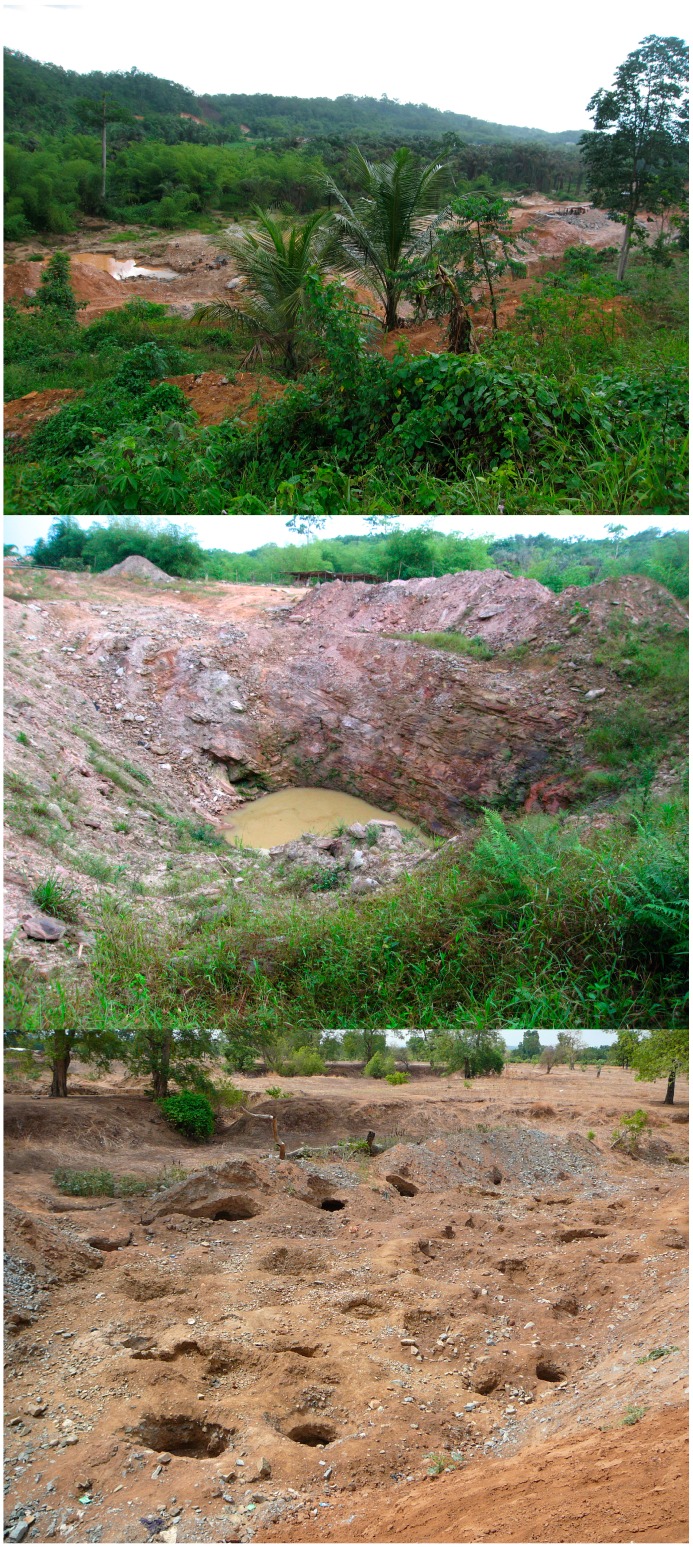
Images of land disturbances at ASGM sites in Tarkwa, Western Region (upper and middle photos) and the Talensi District, Upper East Region (bottom photo).

#### 2.4.3. Consequences

ASGM landscapes are often significantly altered from their previous natural state, with vegetation and soil layers removed and land left with piles of waste tailings, shallow dug-outs, and deep pits (Figure 12) [91]. The ecological and human health risks posed by un-reclaimed and abandoned mines have been documented [9,103,104], but current country-wide estimates of the extent of damage specifically attributable to ASGM are sparse. The most widely cited figure estimates 15,000 ha of land in Ghana was disturbed by small-scale mining in 1995 [105]. This was before the rapid expansion of the industry, and thus the current extant of land disturbance is likely much greater.

Clearing topsoil to make way for mining activity can reduce agricultural and ecological productivity and leave land more susceptible to erosion and desertification [90,94]. Mining in forest areas can threaten remaining forests through fragmentation, loss of biodiversity, and land degradation. In a Denkyira area survey, only 54% of miners stored the removed topsoil and subsoil for later reclamation [21]. Without reclamation, unfilled excavation pits not only become a physical hazard for humans and animals but can capture water and serve as breeding areas for mosquitoes [104]. Land disturbances due to mining, especially near riparian zones, are also believed to be linked to the incidence of buruli ulcer cases [106,107]. 

ASGM also poses additional stressors to wildlife. Though we are not aware of any data, it is highly possible that noise, light, and habitat fragmentation from ASGM communities have varying impacts on wildlife, depending on species type (e.g., migratory birds, amphibians, reptiles). Generators, grinding machines, and ore pounding activities, and artificial lights can produce loud, disruptive noises and light pollution, which have shown potential for wildlife disruptions in other settings [108,109]. Roads, increased vehicular and pedestrian traffic, and deforestation can reduce overall habitat land and fragment existing habitats for wildlife [110]. 

#### 2.4.4. Certainty Evaluation

There is low-moderate certainty on increasing deforestation owing to ASGM activities. Deforestation is known to occur, but the extent tied to ASGM, and even large-scale gold mining, is largely unknown. Land degradation is multifaceted and can occur from mining, industrial, and agricultural activities alike, but despite other contributing factors, ASGM activities often leave behind greatly disturbed landscapes. ASGM may also impact farming practices and encourage miners and mining communities to farm on marginal land that may be more prone to soil erosion or desertification [98]. 

### 2.5. Other Plausible Ecological Effects

With limited data available, other ecological effects associated with ASGM cannot be thoroughly reviewed. Nevertheless, issues such as climate change and mine waste deserve consideration and further investigation.

#### 2.5.1. Climate Change 

Climate change is a global phenomenon with global causes, mainly dealing with increasing releases of greenhouse gases (e.g., carbon dioxide, methane, nitrous oxides, *etc*.) and losses of carbon sinks (e.g., deforestation, loss of permafrost, acidification of oceans, *etc*.). Between 1990 and 2006, Ghana’s greenhouse gas (GHG) emissions rose 242.3% and accounted for 0.05% of total global emissions. Ninety-six percent of these emissions have occurred as a result of land use changes and forestry losses (*i.e.*, losses of carbon sinks). Nationally, the energy sector is the largest contributor to GHG emissions, accounting for 39% of emissions in 2006, driven mainly by the transportation and domestic sectors [111]. 

While Ghana is not a major contributor to GHG emissions, ASGM activities are tied to deforestation and fossil fuel use, which are contributors to climate change. Many analyses, however, are unable to parcel out ASGM from large-scale gold mining impacts on forests. No detailed study has been conducted in Ghana to investigate impacts of gold mining either by large-scale or small-scale mining on climate change, but the land use changes described above and the reliance on fossil fuels for certain tasks mean that GHG emissions, though potentially insignificant compared to other sectors, could be worth documenting. 

More than its contribution to climate change, the effects of climate change on the mining sector may be of concern, especially as it drives shifts in activity or practices. Ghana’s economy relies heavily on sectors that are sensitive to changes in climate, such as agriculture, fisheries, tourism, forest services, *etc*. ASGM already sees influxes in labor when farming becomes unviable [112,113], and this pattern could continue if rural livelihoods face pressure from climatic changes, potentially exacerbating the ecological issues described above. 

Without a robust body of research documenting the effects of climate change in Ghana, reliable predictions on the impact within the mining sector cannot be made. Changing patterns in rainfall and temperature, however, are beginning to be documented [114]. This area of research is ripe with opportunity. The physical, social, and economic dimensions of climate change as they relate to rural livelihoods could help shape effective policy for climate change mitigation and adaptation that supports improvements in ASGM.

#### 2.5.2. Mining Waste

**Figure 13:**
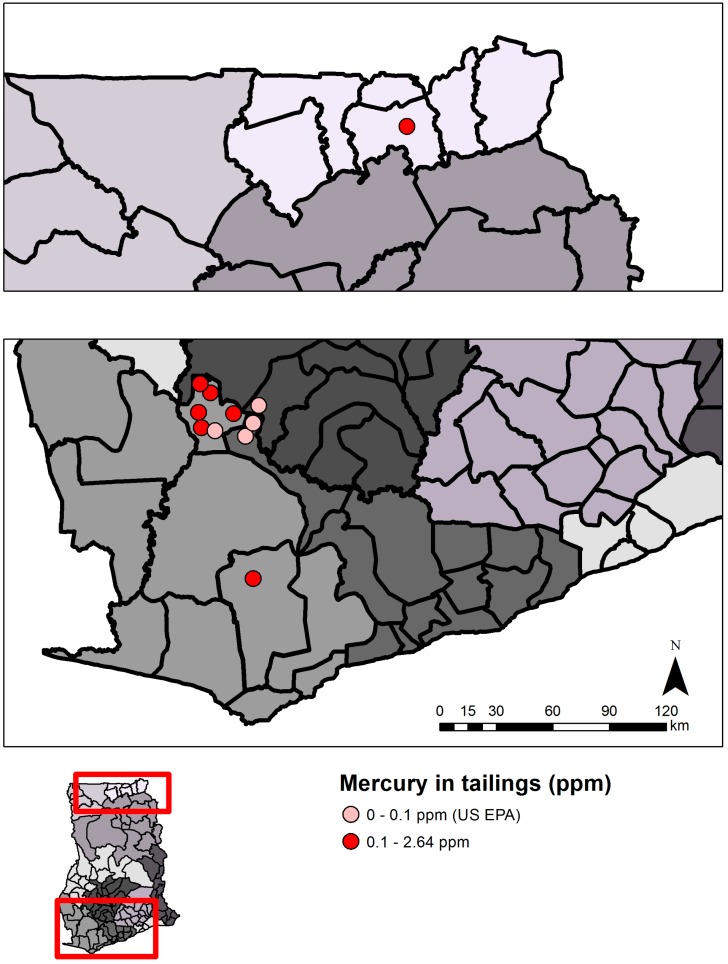
Mercury in tailings in southwest (bottom panel) and northern (upper panel) Ghana. Each circle represents the mean mercury value (µg/g or ppm) above or below the U.S. EPA guideline from a single study in that region, and for illustrative convenience the symbols are scattered randomly within the district where the sampling took place. Political regions are distinguished by different shades of grey. Samples were taken from dumps, dams, or sediments (surface and up to 60 cm) near current or former ASGM sites.

Mine tailings, mostly crushed ore and rock, pose potential threats to water quality, ecosystem, and human health. Proper management of this waste often requires extensive trucking to offsite locations; however, this is not possible in resource-limited settings, and waste generally sits indefinitely in the communities, either in sedimentation ponds or piles [103,115]. Of licensed small-scale gold mining operators in the Offin River, only about 21% treat tailings before discharging them into the Offin River, while 52% discharge directly without treatment, and 27% store tailings in mining pits [21]. While no studies have documented the direct effects of untreated waste on ecosystems, levels of mercury in and around tailings in old and current ASGM sites have been measured in ore tailings, soil, and sediment in some areas, with total mercury concentrations ranging from 0.011 µg/g to 19.3 µg/g (Figure 13, Table S6). Water samples from mine pits, one of which was used in brewing a local drink, in a Talensi District ASGM community contained levels of metals above WHO drinking water standards (e.g., aluminum [Al]: 156 to 6262 ppb, As: 34 to 197 ppb). Samples from gold-washing pools were even higher, with one pool containing levels of Cr, Mn, Ni, Cu, Zn, and Pb over 1,000 µg/L, and Al over 460,000 µg/L [91]. There is a particular lack of data on heavy metals concentrations in ore and the surrounding environ at various states of ore processing. Further research is needed in order to understand the short and long-term impact of mine waste on soil, sediment, water quality, and health. 

**Table 3:** Summary of ecological causes, status and trends, consequences, and certainty of ASGM impacts in Ghana.

	Causes	Status and Trends	Consequences	Certainty Evaluation
**Mercury contamination**	Mercury is used in ASGM to isolate gold from other minerals; mercury is not recaptured	Mercury in soil, sediment, and water is higher in ASGM and LSGM areas than non-mining areas.	Health effects on wildlife and ecological systems; human exposure via contamination of soil, sediment, water, and food.	High
**Contamination from other heavy metals**	Mobilization of toxic elements during excavation, grinding, and washing ore	Arsenic is elevated in soil, and sediment in ASGM and LSGM areas compared to non-mining areas; arsenic is elevated more in some mining sites compared to non-mining sites in water. Cadmium is higher in soil and water in ASGM areas. Lead is elevated slightly in sediment and plants, and in water in ASGM areas.	Health effects on wildlife and ecological systems; human exposure via contamination of soil, sediment, water, and food.	Moderate
**Water quality**	Ore washing, panning, and amalgamation preparation	Water acidity (lower pH) in ASGM and LSGM areas. High turbidity and chemical oxygen demand in mining areas. Other water quality parameters (conductivity, sulfates, and total dissolved solids) were within WHO standards.	Effects on aquatic life and human health via pollution, siltation, and excessive water withdrawals.	Moderate
**Land disturbances**	Vegetation and forests cleared for mining; excavation; temporary human settlements	Decreased natural land cover, shrub densities, and biodiversity in ASGM areas.	Deforestation; erosion; stress to wildlife; and loss of wildlife habitats and biodiversity.	Low-moderate
**Climate change**	Fossil fuel combustion, deforestation, industrial pollution	Greenhouse gas emissions increased mainly from land use changes and forestry losses.	Changes in agricultural and fishing patterns, desertification, and rising sea levels.	Low
**Mining waste**	Ore processing and unregulated tailings disposal	Tailings often not treated before discharging into rivers, deposition, or storage.	Elevated metals in tailings water and mercury in tailings.	Low

## 3. Conclusions

A number of ecological issues have been linked to ASGM in Ghana. Heavy metal contamination can result from the mining process, with elemental mercury used directly for amalgamation and other heavy metals such as arsenic present in gold ore. Mercury contamination in ecological media, including soil, foodstuffs, sediment, water, and fish, has been documented near ASGM sites in southwest Ghana, with soil and water concentrations generally lower than guideline values, but 59% of mean sediment concentrations from ASGM sites above guideline values. Concentrations exceeded WHO and Ghana (GSB/GWC) water standards for arsenic in 62%, cadmium in 19%, and lead in 21% of all sites with ASGM and/or LSGM. Other water quality parameters near ASGM sites show impairment, with some samples exceeding guidelines for acidity, turbidity, and nitrates. Deforestation and land degradation often accompany ASGM activity, potentially decreasing biodiversity, farmland, and soil fertility. While not well-documented, effects from and on climate change as well as contamination from mining waste may add additional stress on environmental quality and ecosystem services. The causes, status and trends, consequences, and certainty evaluations are summarized in Table 3. Spatial and temporal trends in each of these areas are not well-characterized, if at all, especially in western and northeastern Ghana. 

More research is needed to further elucidate the nature of the relationship between ASGM and ecological changes, and this paper has identified several research areas in need of contributions. However, the existing research on plausible ecological consequences of ASGM can help guide policies and actions to better address the unique challenges ASGM. All of this is particularly warranted and timely given that Ghana has signed the 2013 UNEP Minamata Convention on Mercury Pollution. This Convention has specific provisions concerning ASGM (Article 7), and in particular education, outreach and capacity-building initiatives (7.4B), as well as baseline estimates of the quantities of mercury used and the practices employed in artisanal and small-scale gold mining and processing within its territory (Annex C, 1d).

In light of this synthesis of extant and emerging data on the ecological issues associated with ASGM, along with data from public health [9] and social sciences and economics [8], members of our research team developed response options to address ASGM-related concerns in Ghana using the Delphi method [116]. The data presented here, along with these response options, will provide insight that relevant stakeholders can use to implement enduring solutions to the myriad challenges posed by ASGM in Ghana. 

## Supplementary Files

Supplementary File 1
