# Supplementary material for: Integrated Assessment of Artisanal and Small-Scale Gold Mining in Ghana—Part 2: Natural Sciences Review
Source: Int J Environ Res Public Health. 2015 Jul 31;12(8):8971–9011. doi: 10.3390/ijerph120808971 (PMC4555259; doi:10.3390/ijerph120808971)
Supplement: Supplementary File 1 [file ijerph-12-08971-s001.pdf]

SUPPLEMENTAL TABLES FOR:

Integrated Assessment of Artisanal and Small-Scale Gold Mining in Ghana - Part 2: Natural Sciences Review

Table of Contents

Table S1	Total mercury (Hg) levels in soils near Ghanaian small-scale gold mining sites and reference non-mining sites.	1
Table S2	Total mercury (Hg) levels in edible plants near Ghanaian small-scale gold mining sites.	5
Table S3	Total mercury (Hg) levels in sediment near Ghanaian small-scale gold mining sites and reference non-mining sites.	7
Table S4	Total mercury (Hg) levels in water near Ghanaian small-scale gold mining sites and reference non-mining sites.	11
Table S5	Total mercury (Hg) levels in fish and shellfish in Ghana and the Gulf of Guinea.	15
Table S6	Total mercury (Hg) levels in lichen and seaweed bioindicators and tailings in Ghana and from the Gulf of Guinea.	22
Table S7	Total arsenic (As) levels in soils near Ghanaian small-scale mining sites and reference non-mining sites.	25
Table S8	Total arsenic (As) levels in edible plants near Ghanaian small-scale mining sites and reference non-mining sites.	26
Table S9	Total arsenic (As) levels in sediment near Ghanaian small-scale mining sites and reference non-mining sites.	27
Table S10	Total arsenic (As) levels in water near Ghanaian small-scale mining sites and reference non-mining sites.	28
Table S11	Total cadmium (Cd) levels in soils near Ghanaian small-scale mining sites and reference non-mining sites.	31
Table S12	Total cadmium (Cd) levels in edible plants near Ghanaian small-scale mining sites and reference non-mining sites.	32
Table S13	Total cadmium (Cd) levels in sediment near Ghanaian small-scale mining sites and reference non-mining sites.	33
Table S14	Total cadmium (Cd) levels in water near Ghanaian small-scale mining sites and reference non-mining sites.	34
Table S15	Total lead (Pb) levels in soils near Ghanaian small-scale mining sites and reference non-mining sites.	36
Table S16	Total lead (Pb) levels in edible plants near Ghanaian small-scale mining sites and reference non-mining sites.	37
Table S17	Total lead (Pb) levels in sediment near Ghanaian small-scale mining sites and reference non-mining sites.	38
Table S18	Total lead (Pb) levels in water near Ghanaian small-scale mining sites and reference non-mining sites.	39

Table S19	Guidelines and standards for mercury (Hg) in ecological media. Data are reviewed by the number of sampling sites exceeding guideline values for total, mining (ASGM and/or LSGM), and non-mining sampling sites.	41
Table S20	Guidelines and standards for arsenic (As) in ecological media. Data are reviewed by the number of sampling sites exceeding guideline values for total, mining (ASGM and/or LSGM), and non-mining sampling sites.	42
Table S21	Guidelines and standards for cadmium (Cd) in ecological media. Data are reviewed by the number of sampling sites exceeding guideline values for total, mining (ASGM and/or LSGM), and non-mining sampling sites.	43
Table S22	Guidelines and standards for lead (Pb) in ecological media. Data are reviewed by the number of sampling sites exceeding guideline values for total, mining (ASGM and/or LSGM), and non-mining sampling sites.	44
	References	45

Table S1. Total mercury (Hg) levels in soils near Ghanaian small-scale gold mining sites and reference non-mining sites.

Study location	Year collected	Dry/wet season	Sample size (n)	Mean Hg (ng/g)	SD Hg (ng/g)	Min. Hg (ng/g)	Max. Hg (ng/g)	Reference	Notes
Dumasi Area (soil from active amalgamation site), Wassa West District, Western Region	2001	-	2	6092	-	-	-	[1]	Data has been extracted from a book chapter that was contributed to by the authors.
Dumasi area (soil from a miner's house with roasting activity), Wassa West District, Western Region	2001	-	2	21639	-	-	-	[1]	Data has been extracted from a book chapter that was contributed to by the authors.
Dumasi, Wassa West District, Western Region	-	-	12	240	100	130	420	[2]	Samples measured from farms near large-scale and ASGM; 5.5 km from mines. Nine surface samples and four samples taken from three soil depths (0-5cm, 5-15cm, and 15-30cm) at each site.
Dumasi, Wassa West District, Western Region	-	-	9	210	90	100	350	[2]	Samples measured from farms near large-scale and ASGM; 6.0 km from mines. Nine surface samples and four samples taken from three soil depths (0-5cm, 5-15cm, and 15-30cm) at each site.
Bogoso, Wassa West District, Western Region	-	-	12	230	50	120	280	[2]	Samples takenfrom farms near entrance of Bogoso Gold Ltd; 1.5 km from mines. Nine surface samples and four samples taken from three soil depths (0-5cm, 5-15cm, and 15-30cm) at each site.
Bogo, Wassa West District, Western Region	-	-	12	190	70	90	350	[2]	Samples measured from farms near large-scale and ASGM; 8.4 km from mines. Nine surface samples and four samples taken from three soil depths (0-5cm, 5-15cm, and 15-30cm) at each site.
Prestea Ankroba, Wassa West District, Western Region	-	-	12	280	70	170	400	[2]	Samples measured from farms near large-scale and ASGM; 14.0 km from mines. Nine surface samples and four samples taken from three soil depths (0-5cm, 5-15cm, and 15-30cm) at each site.

ND = Not detectable or below the detection limit.

Table S1. Total mercury (Hg) levels in soils near Ghanaian small-scale gold mining sites and reference non-mining sites.

Study location	Year collected	Dry/wet season	Sample size (n)	Mean Hg (ng/g)	SD Hg (ng/g)	Min. Hg (ng/g)	Max. Hg (ng/g)	Reference	Notes
Prestea Old Mine, Wassa West District, Western Region	-	-	12	310	50	220	400	[2]	Samples measured from farms near large-scale and ASGM; 16.0 km from mines. Nine surface samples and four samples taken from three soil depths (0-5cm, 5-15cm, and 15-30cm) at each site.
Tarkwa area (soil from abandoned mining/ processing site), Wassa West District, Western Region	2001	-	2	185,938	-	-	-	[1]	Data has been extracted from a book chapter that was contributed to by the authors.
Dompem, Wassa West District, Western Region	2006 - 2007	w/d	36	320	80	260	410	[3]	Samples were collected monthly (for 10 months; Sep. 2006 to Jun. 2007) from farms sites where large and small-scale mining occurs, at three depths (0-5cm, 10-15cm, and 25-30cm).
Tarkwa, Wassa West District, Western Region		w/d	36	370	140	210	480		
Aboso, Wassa West District, Western Region		w/d	36	2400	610	1700	2800		
Huni Valley, Wassa West District		w/d	36	290	40	260	330		
Atieku, Wassa West District, Western Region		w/d	36	120	20	100	130		
Wassa West District, Western Region	-	-	5	792	61.4	750	900	[4]	Data from five mining communities Iduapreim, Odumasi, Hemang, Huni Valley, and Damang.
River Ankobra, Western Region	-	-	7	150	-	51	366	[5]	This work was carried using the atomic absorption spectrum. Results were compared with literature and found to be consistent. Surface soil samples (0-10 cm) collected close to the main River Ankobra and its major tributaries at Ankobra, Bonsa, Subri, Beppoh, Bawdie, Terebebie, and Efuanta.
Bibiani, Western Region	2007	w	10	31	21	7	63	[6]	Data from farms/gardens in four communities with small and large-scale mining (Hwenampori, Lineso, New Town, Old Town) in Sept. 2007, up to 15-20cm depth.

ND = Not detectable or below the detection limit.

Table S1. Total mercury (Hg) levels in soils near Ghanaian small-scale gold mining sites and reference non-mining sites.

Study location	Year collected	Dry/wet season	Sample size (n)	Mean Hg (ng/g)	SD Hg (ng/g)	Min. Hg (ng/g)	Max. Hg (ng/g)	Reference	Notes
Ajumamu in Dunkwa area (soil at amalgamation site near river bank), Upper Denkyira East Municipal District, Central Region	2001	-	2	933.31	-	-	-	[1]	Data has been extracted from a book chapter that was contributed to by the authors.
Obuasi, Ashanti Region	1992 - 1993	w/d	168	900	600	300	2500	[7]	Samples collected once a month (Mar 1992 - Feb 1993) from from 14 sites located 0.31 - 17.5 km from Pompora (water) Treatment Plant. Both large and small-scale mining occurs nearby. Three samples per site.
River Pra Basin, Western, Central & Ashanti Regions	2002	w	27	427	1,310	11	5,547	[8]	Samples taken from the Rivers Offin, Upper Pra and Lower Pra, once in the wet & dry season each.
	2003	d	11	20	10	6	37		
River Pra Basin, Western, Central & Ashanti Regions	2005 - 2006	-	5	84	38	42	145	[9]	Data from five mining communities at the River Pra (Awisam, Twifo Praso, Twifo Mampong, Daboase, and Beposo).
Kejetia ASG mine, Talensi District, Upper East Region	2010	w	17	4785.2	9780.5	95.65	40,969.60	[10]	Household soil samples collected in May-June 2010 from the top 1-2 cm of soil.
Kejetia ASG mine, Talensi District, Upper East Region	2011	w	54	15,550	46,890	296.7	330,040	[10]	Household soil samples collected in June-July 2011 from the top 1-2 cm of soil.
ASGM gold refinery, Bolgatanga, Upper East Region	2012	w	4	57,752.7	58,217.0	5430.0	116,440.20	[10]	Samples taken from a refinery in Bolgatanga in August 2012 from the top 1-2 cm of soil. Samples were collected from within the refinery and at 3 and 6 meters away.

ND = Not detectable or below the detection limit.

Table S1. Total mercury (Hg) levels in soils near Ghanaian small-scale gold mining sites and reference non-mining sites.

Study location	Year collected	Dry/wet season	Sample size (n)	Mean Hg (ng/g)	SD Hg (ng/g)	Min. Hg (ng/g)	Max. Hg (ng/g)	Reference	Notes
Reference sites: Non-mining locations									
Wassa West District, Western Region	2006 - 2007	w/d	108	134	54	60	210	[3]	Samples were collected monthly (for 10 months; Sep. 2006 to Jun. 2007) from farms sites where no mining activity occurs (Agric farm, Shama Junction, Takarodi, Agona Nkwanta), at three depths (0-5cm, 10-15cm, and 25-30cm).
Wassa West District, Western Region	-	-	3	ND	ND	ND	ND	[4]	Data from three non-mining communities: Ayanfuri, Wassa Asikuma, and Krofofrum (all not detectable levels; no level of detection given).
Otsew, Gomoa West District, Central Region	2010	w	1	190	20	-	-	[11]	Sample of earthenware clay soil was purchased from a market in November 2010.
Otsew, Gomoa West District, Central Region	2010	w	10	140	-	30	310	[11]	Samples from earthenware clay soil deposits from 30cm depth in November 2010.
River Pra Basin, Western, Central & Ashanti Regions	2002	w	7	99.0	102	8	260	[8]	Samples taken from the Rivers Offin, Upper Pra and Lower Pra in non-mining areas, once in the wet & dry seasons each.
	2003	d	7	170	413	7	1106		
Gorogo, Nabdam District, Upper East Region	2011	w	26	41	23	13	114	[10]	Household soil samples collected in July 2011 from top 1-2 cm of soil.

ND = Not detectable or below the detection limit.

Table S2. Total mercury (Hg) levels in edible plants near Ghanaian small-scale gold mining sites.

Study location	Year collected	Dry/wet season	Species (common name)	Sample size (n)	Mean Hg (µg/g)	SD Hg (µg/g)	Min. Hg (µg/g)	Max. Hg (µg/g)	Reference	Notes
Obuasi, Ashanti Region	1992 - 1993	w/d	Manihot esculenta (cassava)	144	0.7083	-	-	-	[7]	Samples collected once a month from Mar to Feb from 12 villages around the Pompora Treatment Plant at Obuasi (3 samples per site).
Wassa West District, Western Region	-	-	Manihot esculenta (cassava)	8	0.2	0.203	0	0.56	[4]	Samples collected from farms at 5 gold mining towns (Iduapreim, Odumasi, Hemang, Huni Valley, Damang) and 3 non-mining towns (Ayanfuri, Wassa Asikuma, Krofofrom).
Bogoso, Wassa West District, Western Region	-	-	Manihot esculenta (flesh only; cassava)	4	0.13	0.03	0.10	0.16	[2]	Samples takenfrom farms near entrance of Bogoso Gold Ltd; 1.5 km from mines. Tubers collected from three plants.
Dumasi, Wassa West District, Western Region	-	-	Manihot esculenta (flesh only; cassava)	4	0.17	0.01	0.15	0.18	[2]	Samples measured from farms near large-scale and ASGM; 6.0 km from mines. Tubers collected from three plants.
Bogo, Wassa West District, Western Region	-	-	Manihot esculenta (flesh only; cassava)	4	0.12	0.04	0.08	0.17	[2]	Samples measured from farms near large-scale and ASGM; 8.4 km from mines. Tubers collected from three plants.
Prestea Ankroba, Wassa West District, Western Region	-	-	Manihot esculenta (flesh only; cassava)	4	0.15	0.02	0.13	0.18	[2]	Samples measured from farms near large-scale and ASGM; 14.0 km from mines. Tubers taken from three plants.
Tarkwa, Western Region	-	-	Xanthosoma sagittifolium (cocoyam)	1	0.003	-	-	-	[12]	Samples collected from a mining town (Efuantah).
Wassa West District, Western Region	-	-	Xanthosoma sagittifolium (cocoyam)	8	0.375	0.323	0	0.67	[4]	Samples collected from farms at 8 gold mining towns (Iduapreim, Odumasi, Hemang, Huni Valley, Damang, Ayanfuri, Wassa Asikuma, and Krofofrom).
Tarkwa, Western Region	-	-	Colocasia esculenta (water cocoyam)	2	0.0035	0.002	0.002	0.005	[12]	Samples collected from 2 mining towns (Nsuta, and Tamso),
Wassa West District, Western Region	-	-	Colocasia esculenta (water cocoyam)	8	0.374	0.315	0	0.66	[4]	Samples collected from farms at 8 gold mining towns (Iduapreim, Odumasi, Hemang, Huni Valley, Damang, Ayanfuri, Wassa Asikuma, and Krofofrom).

Table S2. Total mercury (Hg) levels in edible plants near Ghanaian small-scale gold mining sites.

Study location	Year collected	Dry/wet season	Species (common name)	Sample size (n)	Mean Hg (μg/g)	SD Hg (μg/g)	Min. Hg (μg/g)	Max. Hg (μg/g)	Reference	Notes
Obuasi, Ashanti Region	1992 - 1993	w/d	Musa paradisiaca (plantain)	156	0.6846	-	-	-	[7]	Samples collected once a month from Mar to Feb from 13 villages around the Pompora Treatment Plant at Obuasi (3 samples per site).
Wassa West District, Western Region	-	-	Musa paradisiaca (plantain)	8	0.283	0.279	0	0.65	[4]	Samples collected from farms at 5 gold mining towns (Iduapreim, Odumasi, Hemang, Huni Valley, Damang) and 3 non-mining towns (Ayanfuri, Wassa Asikuma, Krofofrom).
Obuasi, Ashanti Region	1992 - 1993	w/d	Ceratopteris cornuta (water fern)	168	3.4214	-	-	-	[7]	Samples collected once a month from Mar to Feb from 14 villages around the Pompora Treatment Plant at Obuasi (3 samples per site).
Obuasi, Ashanti Region	1992 - 1993	w/d	Pennisetum purpureum (elephant grass)	120	2.580	-	-	-	[7]	Samples collected once a month from Mar to Feb from 10 villages around the Pompora Treatment Plant at Obuasi (3 samples per site).

Table S3. Total mercury (Hg) levels in sediment near Ghanaian small-scale gold mining sites and reference non-mining sites.

Study location	Year collected	Dry/wet season	Sample size (n)	Mean Hg (ng/g)	SD Hg (ng/g)	Min. Hg (ng/g)	Max. Hg (ng/g)	Reference	Notes
Dumasi, Wassa West District, Western Region	2000	d	-	-	-	640	8500	[13]	Samples were taken from the Rivers Apopre and Rora in April 2000.
Dumasi Area (sediment from River Apopre), Wassa West District, Western Region	2001	-	2	4338	-	-	-	[1]	Data has been extracted from a book chapter(12) that was contributed to by the authors of the article. Galamsey tailing pond discharges into River Apopre.
Prestea, Wassa West District, Western Region	2002 - 2003	d	13	25,600	23250	1200	84300	[14]	Samples collected from Jan. through Apr. (Water & sediment samples were collected together.) The top 10-15 cm of sediment were collected. Six sample replicates were measured at each site (13 sites; 4 from Dagarti compound, 9 from Bondae; samples higher at Dagarti where galamsey is more common).
Tarkwa area (sediment from a fishing pond near galamsy site), Wassa West District, Western Region	2001	-	2	40,848	-	-	-	[1]	Data has been extracted from a book chapter(12) that was contributed to by the authors of the article.
River Angonabeng, Tarkwa, Western Region	2008 - 2009	d	5	212	149	-	-	[15]	Samples collected once a month from Sep. 2008 to Jan. 2009 from the stream.
River Bediabedwu, Tarkwa, Western Region	2008 - 2009	d	5	54	38	-	-	[15]	Samples collected once a month from Sep. 2008 to Jan. 2009 from the stream.
Spring 2, Teberebie Goldfields Limited, Tarkwa, Western Region	2008 - 2009	d	5	60	20	-	-	[15]	Samples collected once a month from Sep. 2008 to Jan. 2009 from springs at Mile 7 and Teberebie in Tarkwa.
Spring 3, Teberebie Goldfields Limited, Tarkwa, Western Region	2008 - 2009	d	5	10	10	-	-	[15]	Samples collected once a month from Sep. 2008 to Jan. 2009 from springs at Mile 7 and Teberebie in Tarkwa.
Mile 7, Iduapriem Anglogold Ashanti Mines, Tarkwa, Western Region	2008 - 2009	d	5	ND	-	-	-	[15]	Samples collected once a month from Sep. 2008 to Jan. 2009 from springs at Mile 7 and Teberebie in Tarkwa.

ND = Not detectable or below the detection limit.

Table S3. Total mercury (Hg) levels in sediment near Ghanaian small-scale gold mining sites and reference non-mining sites.

Study location	Year collected	Dry/wet season	Sample size (n)	Mean Hg (ng/g)	SD Hg (ng/g)	Min. Hg (ng/g)	Max. Hg (ng/g)	Reference	Notes
River Ankobra, Western Region	2001	w	-	-	-	60	1630	[16]	Samples collected in Sep. 2001 and Feb. 2002 from five ares on the River Ankobra: Ankwaso, Prestea, Dombase (all on the main River Ankobra), Beppo (Manse tributary) and Bonsaso (Bonsa tributary), and one area at Anwiaso (10km from the source).
River Ankobra, Western Region	2002	d	-	-	-	60	3340	[16]	Samples collected in Sep. 2001 and Feb. 2002 from five ares on the River Ankobra: Ankwaso, Prestea, Dombase (all on the main River Ankobra), Beppo (Manse tributary) and Bonsaso (Bonsa tributary), and one area at Anwiaso (10km from the source).
River Ankroba basin, Wassa West District, Western Region	-	-	6	139	87.65	63	270	[17]	Sediment from channel-bed alluvium. Samples were collected in the mining towns of Nsuta, Tarkwa, Damang, Abosso, Bogoso, and Prestea, in a tropical rainforest corridor. Large, small-scale and illegal mining ocurrs in the area.
Bibiani, Anwiaso, Bekwai District; Western Region	2008 - 2009	d	-	482	427	198	1115	[18]	Samples collected from waterways in the dry season from Oct 2008 to Mar 2009 in four sites affeced by mining (at Mpokwampa, Kyereyar, Pamonu stream, and Lake Amponsah). Surface sediments (0 to ~10 cm) were taken where water samples were also collected (~60 samples total for entire study).
Dunkwa-on-Offin & Keniago, River Offin, Central Region	2000	w/d	-	546	523	30	1631	[19]	Samples were collected once in July, Aug., Sep., and Feb. from each river edge with 2-3 feet PVC tubing immersed to the bottom sediment, with 6 samples at each riverbank on the Rivers Pra (2 sites), Fum (1 site), and Offin (3 sites).
Ajumamu in Dunkwa area (sediment from River Offin), Upper Denkyira East Municipal District, Central Region	2001	-	2	133.94	-	-	-	[1]	Data has been extracted from a book chapter(12) that was contributed to by the authors of the article.

ND = Not detectable or below the detection limit.

Table S3. Total mercury (Hg) levels in sediment near Ghanaian small-scale gold mining sites and reference non-mining sites.

Study location	Year collected	Dry/wet season	Sample size (n)	Mean Hg (ng/g)	SD Hg (ng/g)	Min. Hg (ng/g)	Max. Hg (ng/g)	Reference	Notes
Dunkwa-on-Offin, Upper Denkyira East Municipal District, Central Region	2006	d/w	4	186.15	77.18	-	-	[20]	Samples collected from town along River Offin in Feb. and Jul. 2006.
Awisam, Central Region	2006	d/w	4	96.11	30.7	-	-	[20]	Samples collected from town along River Offin in Feb. and Jul. 2006
Nkotumso, Central Region	2006	d/w	4	1010.08	102.89	-	-	[20]	Samples collected from town along River Offin in Feb. and Jul. 2006
Twifo Praso, River Pra, Central Region	2000	w/d	-	622	679	41	1797	[19]	Samples were collected once in July, Aug., Sep., and Feb. from each river edge with 2-3 feet PVC tubing immersed to the bottom sediment, with 6 samples at each riverbank on the Rivers Pra (2 sites), Fum (1 site), and Offin (3 sites).
River Pra Basin, Western, Central & Ashanti Regions	2002	w	11	171	71	22	678	[8]	Samples were collected in the wet and dry seasons of the year to observe the variation in (total) mercury levels during these two major seasons.
	2003	d	11	14	7	2	43		
River Pra Basin; Western, Central & Ashanti Regions	2005 - 2006	w/d	5	536	116	390	707	[9]	Data from five mining communities at the River Pra (Awisam, Twifo Praso, Twifo Mampong, Daboase, and Beposo) between Aug. 2005 and Jan. 2006.
Buabenso, Ashanti Region	2006	d/w	4	424.3	260.12	-	-	[20]	Samples collected from town along River Offin in Feb. and Jul. 2006
Dominase, Bekwai Municipal District, Ashanti Region	2006	d/w	4	144.55	38.21	-	-	[20]	Samples collected from town along River Offin in Feb. and Jul. 2006
Fumso, River Fum, Ashanti Region	2000	w/d	-	463	475	47	1127	[19]	Samples were collected once in July, Aug., Sep., and Feb. from each river edge with 2-3 feet PVC tubing immersed to the bottom sediment, with 6 samples at each riverbank on the Rivers Pra (2 sites), Fum (1 site), and Offin (3 sites).

ND = Not detectable or below the detection limit.

Table S3. Total mercury (Hg) levels in sediment near Ghanaian small-scale gold mining sites and reference non-mining sites.

Study location	Year collected	Dry/wet season	Sample size (n)	Mean Hg (ng/g)	SD Hg (ng/g)	Min. Hg (ng/g)	Max. Hg (ng/g)	Reference	Notes
Birim North District, Eastern Region	2009	w	6	427	194	134	732	[21]	Samples collected upstream once during wet months (Apr - Aug).
	2009 - 2010	d	6	860	604	381	1880		Samples collected upstream once during dry months (Oct - Feb).
	2009	w	6	1380	710	401	2320		Samples collected downstream once during wet months (Apr - Aug).
	2009 - 2010	d	6	3300	1400	1420	4890		Samples collected downstream once during dry months (Oct - Feb).
Talensi District, Upper East Region	2010	w	14	35.53	61.63	4.949	247.5	[10]	Samples were collected in May-June 2010 near ASGM mining sites from a stream and reservoir.
Reference sites: Non-mining locations									
Bibiani, Anwiaso, Bekwai District; Western Region	2008 - 2009	d	-	169	-	-	-	[18]	Samples collected in the dry season from Oct 2008 to Mar 2009 from Asusuo and Abubayenyunu, areas with no known mining activity. Surface sediments (0 to ~10 cm) were taken where water samples were also collected (~60 samples total for entire study).
Shama, Western Region (Pra); and Elmina (Benya), Cape Coast (Fosu), and Saltpong Beach (Narkwa), Central Region, Gulf of Guinea	-	w	16	5	5	0	17	[22]	Samples collected from Pra Estuary, Benya Lagoon, Fosu Lagoon, and Narkwa Lagoon during wet and dry season.
	-	d	16	9	15	0	55		
River Kakum, Central Region	2008 - 2009	d	5	ND	-	-	-	[15]	Samples collected once a month from Sep. 2008 to Jan. 2009 from the stream.
River Pra Basin, Western, Central & Ashanti Regions	2002	w	7	494	79	18	2,917	[8]	Samples taken from the Rivers Offin, Upper Pra and Lower Pra in non-mining areas, once in the wet & dry seasons each.
	2003	d	7	12	8	2	43		
Ada and Aveglo, Volta Estuary, Volta Region	2008 - 2009	w/d	-	-	-	6.9	24.0	[23]	Samples were collected at two sample sites during monthly intervals between March 2008 and August 2009.

ND = Not detectable or below the detection limit.

Table S4. Total mercury (Hg) levels in water near Ghanaian small-scale gold mining sites and reference non-mining sites.

Study location	Year collected	Dry/wet season	Water type	Water source	Sample size (n)	Mean Hg (µg/L)	SD Hg (µg/L)	Min. Hg (µg/L)	Max. Hg (µg/L)	Reference	Notes
River Angonabeng, Tarkwa, Western Region	2008 - 2009	d	Surface water	Stream	5	1484	256	-	-	[15]	Samples collected once a month from Sep. 2008 to Jan. 2009 from the stream.
River Bediabedwu, Tarkwa, Western Region	2008 - 2009	d	Surface water	Stream	5	522	510	-	-	[15]	Samples collected once a month from Sep. 2008 to Jan. 2009 from the stream.
River Ankobra Basin, Western Region	2001	w	Surface water	River	-	-	-	0.05	21	[16]	Samples collected in Sep. 2001 and Feb. 2002 from five areas on the River Ankobra Basin: Ankwaso, Prestea, Dombase, Beppo and Bonsaso, and one area at Anwiaso.
River Ankobra Basin, Western Region	2002	d	Surface water	River	-	-	-	0.053	0.185	[16]	Samples collected in Sep. 2001 and Feb. 2002 from five areas on the River Ankobra Basin: Ankwaso, Prestea, Dombase, Beppo and Bonsaso, and one area at Anwiaso.
Tarkwa, Western Region	2004	d	Surface water	Rivers/streams	12	0.4	-	-	0.4	[24]	Samples collected in March 2004 from rivers/streams. No samples recorded levels above the detection limit of 0.5 µg/L. Values reflect detection limit/sqrt(2).
River Pra basin; Western, Central, and Ashanti Regions	2002	w	Surface water	River	14	0.187	0.137	0.034	0.420	[8]	Samples collected in wet season of 2002 and dry seaon of 2003 from samples at the Lower Pra, Offin, and Upper Pra.
	2003	d	Surface water	River	13	0.187	0.132	0.046	0.462		
Prestea, Western Region	2002 - 2003	d	Surface water	River	13	7160	4900	500	19820	[14]	Samples collected from Jan. through Apr. (Water & sediment samples were collected together.) Six sample replicates were measured at each site (13 sites; 4 from Dagarti compound, 9 from Bondae; samples higher at Dagarti where galamsey is more common).
River Pra, Dunkwa-on-Offin	2000	w/d	Surface water	River	-	0.932	0.796	0.104	1.974	[19]	Data from 2 bridge sites on the river (Twifo Praso, Praso) colected once per month in July, Aug., Sept. and Feb. Nine samples were collected at each bridge site--6 analyzed in a lab and 3 analyzed with a Hg thermometer in the field--at 0.8 and 2 m depths.
River Fum, Dunkwa-on-Offin	2000	w/d	Surface water	River	-	0.783	0.756	0.019	1.818	[19]	Data from 1 bridge site on the river colected once per month in July, Aug., Sept. and Feb. Nine samples were collected at each bridge site--6 analyzed in a lab and 3 analyzed with a Hg thermometer in the field--at 0.8 and 2 m depths.
River Oda, Dunkwa-on-Offin	2000	w/d	Surface water	River	-	0.817	0.888	0.105	2.112	[19]	Data from 1 bridge site on the river colected once per month in July, Aug., Sept. and Feb. Nine samples were collected at each bridge site--6 analyzed in a lab and 3 analyzed with a Hg thermometer in the field--at 0.8 and 2 m depths.

ND = Not detectable or below the detection limit.

Table S4. Total mercury (Hg) levels in water near Ghanaian small-scale gold mining sites and reference non-mining sites.

Study location	Year collected	Dry/wet season	Water type	Water source	Sample size (n)	Mean Hg (µg/L)	SD Hg (µg/L)	Min. Hg (µg/L)	Max. Hg (µg/L)	Reference	Notes
Bibiani, Anwiaso, Bekwai District, Western Region	2008 - 2009	d	Surface water	Streams	-	0.452	0.514	0.124	1.341	[18]	Samples collected from waterways in the dry season from Oct 2008 to Mar 2009 in five sites affeced by mining (at Mpokwampa, Kyereyar, Pamonu stream, Lake Amponsah, and a galamsey tailing site). Surface sediments (0 to ~10 cm) were taken where water samples were also collected (~60 samples total for entire study).
Birim North District, Eastern Region	2009	w	Surface water	Stream (upstream)	6	0.180	0.110	0.045	0.358	[21]	Samples collected over 11 month period (Apr - Feb), once in the wet and once in the dry season.
	2009 - 2010	d		Stream (upstream)	6	0.339	0.057	0.248	0.412		
	2009	w		Stream (downstream)	6	0.553	0.212	0.282	0.872		
	2009 - 2010	d		Stream (downstream)	6	0.860	0.269	0.643	1.343		
Obuasi, Ashanti Region	2008	w	Surface water	Rivers	-	0.287	0.42	0.018	1.002	[25]	Samples collected during wet season (Mar - Jun), once per month (4 times) at 7 sites (Rivers Kaw, Kwabrafo, Pompo, Akopori, Jimi, Akyerempe, and Supu).
Dumasi, Wassa West District, Western Region	2000	d	Surface water	Rivers	5	-	-	0.18	0.76	[13]	Samples were taken from the Rivers Apopre and Rora in April 2000.
River Ankroba Basin, Western Region	-	d	Surface water	Streams	89	1.276	-	0.70	8.00	[17]	Data from 4 stream sites (Iduapriem, Tarkwa, Nsuta [a manganese mining company area], and Damang), collected during the dry season (Nov-Feb) from areas with large and/or small-scale mining.
River Ankroba Basin, Western Region	-	-	Surface & groundwater	Streams, boreholes, & hand-dug wells	70	1.5	1.25	<1	8	[17]	Samples collected from streams, boreholes, and hand-dug wells. Samples were collected in the mining towns of Nsuta, Tarkwa, Damang, Abosso, Bogoso, and Prestea, in a tropical rainforest corridor. Large, small-scale, and illegal mining ocurrs in the area.
Nangodi, Talensi-Nabdam District, Upper East Region	2010 - 2011	d	Surface & groundwater	Borehole, dug wells, & dugouts	30	38.4	58.05	<5	190	[26]	Samples collected between Nov 2010 to Apr 2011. One site from a borehole (Kalin), 2 from dug wells (Nadpadana, Nakpalig), and 2 from dugouts (Kolikpenge, Debahe).
Tinga, Bole-Bamboi District, Northern Region	2011 - 2012	d	Surface & groundwater	Stream & boreholes	42	50	40	10	172	[27]	Samples collected monthly between Oct. 2011 and April 2012 from stream (drinking water and gold ore washing) and 4 boreholes.

ND = Not detectable or below the detection limit.

Table S4. Total mercury (Hg) levels in water near Ghanaian small-scale gold mining sites and reference non-mining sites.

Study location	Year collected	Dry/wet season	Water type	Water source	Sample size (n)	Mean Hg (µg/L)	SD Hg (µg/L)	Min. Hg (µg/L)	Max. Hg (µg/L)	Reference	Notes
Konongo-Odumasi, Asante Akim North District, Ashanti Region	2011 - 2012	d	Surface & ground water	River, streams, wells, & mine pits	36	20	10	2	66	[28]	Samples collected upstream, midstream, and downstream in River Owere in addition to streams, wells, and mine pits between Nov. 2011 and April 2012. Large and small-scale mining present.
Obuasi, Ashanti Region	2009	d/w	Surface & tap water	Surface & tap water	48	4.0363	5.10287	0.001	25.3	[29]	Samples collected from Feb - Jun. in the Obuasi mining area.
Obuasi, Ashanti Region	2008	w	Groundwater	Rivers	-	0.02	0.001	0.019	0.021	[25]	Samples collected during wet season (Mar - Jun), once per month (4 times) at 7 sites (Rivers Kaw, Kwabrafo, Pompo, Akopori, Jimi, Akyerempe, and Supu).
Tarkwa, Western Region	2004	d	Groundwater	Boreholes	12	0.4	-	0.4	2.3	[24]	Samples collected from boreholes in March. Hg detected above detection level of 0.5 in only one sample.
Tarkwa, Western Region	2004	d	Groundwater	Wells	6	0.4	-	0.4	0.5	[24]	Samples collected from wells (n=6) in March. Hg detected above detection level of 0.5 in only one sample.
Spring 2, Teberebie Goldfields Limited, Tarkwa, Western Region	2008 - 2009	d	Groundwater	Springs	5	14	10	-	-	[15]	Samples collected once a month from Sep. 2008 to Jan. 2009 from springs at Mile 7 and Teberebie in Tarkwa.
Spring 3, Teberebie Goldfields Limited, Tarkwa, Western Region	2008 - 2009	d	Groundwater	Springs	5	ND	-	-	-	[15]	Samples collected once a month from Sep. 2008 to Jan. 2009 from springs at Mile 7 and Teberebie in Tarkwa.
Mile 7, Iduapriem Anglogold Ashanti Mines, Tarkwa, Western Region	2008 - 2009	d	Groundwater	Springs	5	ND	-	-	-	[15]	Samples collected once a month from Sep. 2008 to Jan. 2009 from springs at Mile 7 and Teberebie in Tarkwa.
Dumasi, Wassa West District, Western Region	2000	d	Groundwater	Springs	8	-	-	0.12	0.27	[13]	Samples were taken from boreholes near Dumasi in April 2000.
River Lower Offin Basin, Central Region	2004	d	Groundwater	Boreholes, hand-dug wells, & springs	148	0.18	1.8	0.4	3.85	[30]	Samples collected during dry seaon (Dec) from boreholes, hand-dug wells and springs. Minimum below detection level of 0.5 µg/L.
Birim North District, Eastern Region	2009	w	Groundwater	Boreholes	6	0.291	0.193	0.078	0.619	[21]	Samples collected from boreholes, over 11 month period (Apr - Feb), once in the wet & once in the dry season.
	2009 - 2010	d		Boreholes	6	0.420	0.067	0.301	0.482		

ND = Not detectable or below the detection limit.

Table S4. Total mercury (Hg) levels in water near Ghanaian small-scale gold mining sites and reference non-mining sites.

Study location	Year collected	Dry/wet season	Water type	Water source	Sample size (n)	Mean Hg (μg/L)	SD Hg (μg/L)	Min. Hg (μg/L)	Max. Hg (μg/L)	Reference	Notes
Reference sites: Non-mining locations											
River Kakum, Central Region	2008 - 2009	d	Surface water	Stream	5	ND	-	-	-	[15]	Samples collected once a month from Sep. 2008 to Jan. 2009 from the stream.
Shama, Western Region (Pra); and Elmina (Benya), Cape Coast (Fosu), and Saltpong Beach (Narkwa), Central Region, Gulf of Guinea	-	w	Surface water	Estuary & lagoons	16	0.001	0.001	0	0.003	[22]	Samples collected from Pra Estuary, Benya Lagoon, Fosu Lagoon, and Narkwa Lagoon during wet and dry seasons.
		d		Estuary & lagoons	16	0.002	0.002	0	0.005		
River Pra basin; Western, Central, and Ashanti Regions	2002	w	Surface water	River	7	0.119	0.110	0.029	0.296	[8]	Samples collected in wet season of 2002 and dry seaon of 2003 from samples at the Lower Pra, Offin, and Upper Pra.
	2003	d	Surface water	River	7	0.115	0.046	0.072	0.188		
Bibiani, Anwiaso, Bekwai District; Western Region	2008 - 2009	d	Surface water	Streams	60	0.164	0.055	0.125	0.203	[18]	Samples collected twice in the dry season from Oct 2008 to Mar 2009 from Asusuo and Abubayenyunu, at streams with no known mining activity (0 - ~10cm).
Accra, Greater Accra Region	2004	d	Groundwater	Standpipe (tap)	1	0.4	-	-	-	[24]	Samples collected from a standpipe (tap) in Accra in March. No samples over detection level of 0.5 μg/L.

ND = Not detectable or below the detection limit.

Table S5. Total mercury (Hg) levels in fish and shellfish in Ghana and the Gulf of Guinea. Hg concentrations are presented in wet weight (dry weight values were converted to w.w. assuming 80% moisture [32]).

Study location/ Sample collection	Year collected	Dry/Wet Season	Species	Common name(s)	Fresh or Marine	Trophic Level[c]	Sample size (n)	Mean Hg (µg/g)	SD Hg (µg/g)	Min. Hg (µg/g)	Max. Hg (µg/g)	Reference	Notes
Fish													
Upper Volta Basin at Yeji, Brong-Ahafo Region	2006 - 2007	d	Distichodus rostratus	Grass-eater, Perch	F	2	7	0.02052	-	0.01639	0.02583	[31]	Samples were collected from commercial catches at Yeji from Oct. 2006 to Mar. 2007.
River Pra Basin, Western, Central, and Ashanti Regions	2005 - 2006	w/d	Labeo coubie	African carp, Black carp	F	2	45	0.049	0.019	<0.001	0.136	[9]	Samples collected from Twifo Praso, Awisam, Beposo, and Daboase between Aug. 2005 to Jan. 2006 from fishermen.
Nkotumso, Central Region	2006	d/w	Labeo coubie	African carp, Black carp	F	2	6	0.12135	0.03433	0.10083	0.19076	[20]	Samples collected from fishermen at town along River Offin in Feb. and Jul. 2006
Dunkwa-on-Offin, Upper Denkyira East Municipal District, Central Region	2006	d/w	Labeo coubie	African carp, Black carp	F	2	5	0.14377	0.01649	0.12075	0.15790	[20]	Samples collected from fishermen at town along River Offin in Feb. and Jul. 2006
Dominase, Bekwai Municipal District, Ashanti Region	2006	d/w	Oreochromis niloticus	Nile tilapia	F	2	3	0.16531	0.06969	0.09079	0.22887	[20]	Samples collected from fishermen at town along River Offin in Feb. and Jul. 2006
Nkotumso, Central Region	2006	d/w	Oreochromis niloticus	Nile tilapia	F	2	14	0.13213	0.05351	0.05979	0.24751	[20]	Samples collected from fishermen at town along River Offin in Feb. and Jul. 2006
Dunkwa-on-Offin, Upper Denkyira East Municipal District, Central Region	2006	d/w	Oreochromis niloticus	Nile tilapia	F	2	10	0.14539	0.06861	0.06392	0.25976	[20]	Samples collected from fishermen at town along River Offin in Feb. and Jul. 2006
Sakumo Lagoon, Greater Accra Region	2009	d	Sarotherodon melanotheron	Blackchin tilapia	F	2	20	0.007	0.006	< 0.01	0.017	[32]	Samples collected from fisherman between Sept. and Nov. 2009., adjusted to wet weight based on moisture content given in article.
River Pra River, Western, Central, and Ashanti Regions	2003	d	Sarotherodon melanotheron	Blackchin tilapia	F	2	4	0.016	--	-	--	[8]	Dry fish samples were purchased from local fisherman during Janurary 2003 (dry season).
Dominase, Bekwai Municipal District, Ashanti Region	2006	d/w	Sarotherodon melanotheron	Blackchin tilapia	F	2.5	4	0.14633	0.11359	0.08272	0.31642	[20]	Samples collected from fishermen at town along River Offin in Feb. and Jul. 2006
Lake Bosomtwi, Ashanti Region	2004 - 2005	d/w	Tilapia busumana	-	F	2.3	16	0.021	-	0.009	0.049	[33]	Samples collected from commercial catches from fisherman in villages along the Lake from Feb. through Dec.
River Pra Basin, Western, Central, and Ashanti Regions	2005 - 2006	w/d	Tilapia multifasciata (synonym: sarotherodon galilaeus)	Mango tilapia	F	2	15	0.040	0.019	0.013	0.163	[9]	Samples collected from Beposo and Daboase between Aug. 2005 to Jan. 2006 from fishermen.
Lake Bosomtwi, Ashanti Region	2004 - 2005	d/w	Tilapia multifasciata (synonym: sarotherodon galilaeus)	Mango tilapia	F	2	39	0.008	-	<0.001	0.070	[33]	Samples collected from commercial catches from fisherman in villages along the Lake from Feb. through Dec.
Akosombo Reservoir, Eastern Region	2004 - 2005	d/w	Tilapia zilli	Redbelly tilapia	F	2	39	0.012	-	<0.001	0.028	[33]	Samples collected from commercial catches from fisherman in villages along the reservoir from Feb. through Dec.

ND = Not detectable.
a Trophic level from Oppong et al. 2010 [9].
b Trophic level is for *Clarias anguilaris* for Donkor et al., 2006 [8].
c All other trophic levels from FishBase [40].

Table S5. Total mercury (Hg) levels in fish and shellfish in Ghana and the Gulf of Guinea. Hg concentrations are presented in wet weight (dry weight values were converted to w.w. assuming 80% moisture [32]).

Study location/ Sample collection	Year collected	Dry/Wet Season	Species	Common name(s)	Fresh or Marine	Trophic Level[c]	Sample size (n)	Mean Hg (µg/g)	SD Hg (µg/g)	Min. Hg (µg/g)	Max. Hg (µg/g)	Reference	Notes
Kpong Reservoir, Eastern Region	2004 - 2005	d/w	Tilapia zilli	Redbelly tilapia	F	2	9	0.030	-	0.016	0.076	[33]	Samples collected from commercial catches from fisherman in villages along the reservoir from Feb. through Dec.
River Pra Basin, Western, Central, and Ashanti Regions	2005 - 2006	w/d	Tilapia zilli	Redbelly tilapia	F	2	16	0.044	0.022	0.019	0.109	[9]	Samples collected from Twifo Praso and Daboase between Aug. 2005 to Jan. 2006 from fishermen.
Dominase, Bekwai Municipal District, Ashanti Region	2006	d/w	Tilapia zilli	Redbelly tilapia	F	2	13	0.22180	0.08599	0.09330	0.40305	[20]	Samples collected from fishermen at town along River Offin in Feb. and Jul. 2006
Nkotumso, Central Region	2006	d/w	Tilapia zilli	Redbelly tilapia	F	2	6	0.07897	0.01971	0.05716	0.09760	[20]	Samples collected from fishermen at town along River Offin in Feb. and Jul. 2006
Buabenso, Ashanti Region	2006	d/w	Tilapia zilli	Redbelly tilapia	F	2	2	0.16646	0.01815	0.15363	0.17929	[20]	Samples collected from fishermen at town along River Offin in Feb. and Jul. 2006
Lake Bosomtwi, Ashanti Region	2004 - 2005	d/w	Tilapia discolour (synonymn: Coptodon discolor)	-	F	2.2	30	0.017	-	0.066	0.047	[33]	Samples collected from commercial catches from fisherman in villages along the Lake from Feb. through Dec.
Dunkwa-on-Offin, Upper Denkyira East Municipal District, Central Region	2006	d/w	Brycinus sp.	Robber tetra	F	2.2 - 3.3	3	0.14158	0.03916	0.09790	0.17352	[20]	Samples collected from fishermen at town along River Offin in Feb. and Jul. 2006
Kpong Reservoir, Eastern Region	2004 - 2005	d/w	Pelmatochromis guntheri (synonym: chromidotilapia guntheri, hemichromis guntheri)	Guenther's mouthbrooder	F	2.4	8	0.159	-	0.011	0.275	[33]	Samples collected from commercial catches from fisherman in villages along the reservoir from Feb. through Dec.
Upper Volta Basin at Yeji, Brong-Ahafo Region	2006 - 2007	d	Chrysichthys nigrodigitatus	Bagrid catfish, mudfish	F	2.6	29	0.02569	-	0.01048	0.06190	[31]	Samples were collected from commercial catches at Yeji from Oct. 2006 to Mar. 2007.
Dominase, Bekwai Municipal District, Ashanti Region	2006	d/w	Chrysichthys nigrodigitatus	Bagrid catfish, mudfish	F	2.6	5	0.31135	0.08307	0.18275	0.40949	[20]	Samples collected from fishermen at town along River Offin in Feb. and Jul. 2006
Dunkwa-on-Offin, Upper Denkyira East Municipal District, Central Region	2006	d/w	Chrysichthys nigrodigitatus	Bagrid catfish, mudfish	F	2.6	5	0.47324	0.21327	0.26439	0.75683	[20]	Samples collected from fishermen at town along River Offin in Feb. and Jul. 2006
Buabenso, Ashanti Region	2006	d/w	Chrysichthys nigrodigitatus	Bagrid catfish, mudfish	F	2.6	3	0.29940	0.10208	0.21106	0.41116	[20]	Samples collected from fishermen at town along River Offin in Feb. and Jul. 2006
Kpong Reservoir, Eastern Region	2004 - 2005	d/w	Chrysichthys auratus	Golden Nile catfish, Long fin catfish	F	2.7	10	0.041	-	0.026	0.079	[33]	Samples collected from commercial catches from fisherman in villages along the reservoir from Feb. through Dec.
River Pra Basin, Western, Central, and Ashanti Regions	2003	d	Synodontis sp.	Catfish	F	2.7 - 3.4	4	0.192	--	--	--	[8]	Dry fish samples were purchased from local fisherman during Janurary 2003 (dry season).
Buabenso, Ashanti Region	2006	d/w	Synodontis sp.	Catfish	F	2.7 - 3.4	23	0.25714	0.13271	0.11546	0.64940	[20]	Samples collected from fishermen at town along River Offin in Feb. and Jul. 2006
Awisam, Central Region	2006	d/w	Synodontis sp.	Catfish	F	2.7 - 3.4	30	0.28976	0.12560	0.14086	0.79594	[20]	Samples collected from fishermen at town along River Offin in Feb. and Jul. 2006

ND = Not detectable.
a Trophic level from Oppong et al. 2010 [9].
b Trophic level is for *Clarias anguilaris* for Donkor et al., 2006 [8].
c All other trophic levels from FishBase [40].

Table S5. Total mercury (Hg) levels in fish and shellfish in Ghana and the Gulf of Guinea. Hg concentrations are presented in wet weight (dry weight values were converted to w.w. assuming 80% moisture [32]).

Study location/ Sample collection	Year collected	Dry/Wet Season	Species	Common name(s)	Fresh or Marine	Trophic Level[c]	Sample size (n)	Mean Hg (µg/g)	SD Hg (µg/g)	Min. Hg (µg/g)	Max. Hg (µg/g)	Reference	Notes
Upper Volta Basin at Yeji, Brong-Ahafo Region	2006 - 2007	d	Synodontis gambiensis (synonym: synodontis schall)	Catfish, Mandi, Nile squeaker, Shield-headed catfish, Wahrindi	F	2.9	14	0.06951	-	0.04417	0.08588	[31]	Samples were collected from commercial catches at Yeji from Oct. 2006 to Mar. 2007.
Akosombo Reservoir, Eastern Region	2004 - 2005	d/w	Synodontis guntheri (synonym: synodontis membranaceus)	Catfish	F	3.1	6	0.028	-	0.020	0.042	[33]	Samples collected from commercial catches from fisherman in villages along the reservoir from Feb. through Dec.
Upper Volta Basin at Yeji, Brong-Ahafo Region	2006 - 2007	d	Synodontis membranaceus	Catfish	F	3.1	9	0.04315	-	0.00326	0.07993	[31]	Samples were collected from commercial catches at Yeji from Oct. 2006 to Mar. 2007.
Upper Volta Basin at Yeji, Brong-Ahafo Region	2006 - 2007	d	Synodontis ocellifer	Catfish	F	3.1	16	0.02452	-	0.01311	0.03864	[31]	Samples were collected from commercial catches at Yeji from Oct. 2006 to Mar. 2007.
Kpong Reservoir, Eastern Region	2004 - 2005	d/w	Amphilius grammatophorus (synonym: amphilius platychir	Mountain barbel (catfish)	F	3	4	0.170	-	0.141	0.208	[33]	Samples collected from commercial catches from fisherman in villages along the reservoir from Feb. through Dec.
Upper Volta Basin at Yeji, Brong-Ahafo Region	2006 - 2007	d	Gnathonemus senegalensis (synonym: marcusenius senegalensis)	Trunkfish (an elephant fish)	F	3.1	15	0.01921	-	0.01233	0.02418	[31]	Samples were collected from commercial catches at Yeji from Oct 2006 to Mar 2007.
Dominase, Bekwai Municipal District, Ashanti Region	2006	d/w	Heterobranchus sp.	Catfish	F	3.2 - 3.7	5	0.03579	0.00142	0.03477	0.03828	[20]	Samples collected from fishermen at town along River Offin in Feb. and Jul. 2006
Nkotumso, Central Region	2006	d/w	Heterobranchus sp.	Catfish	F	3.2 - 3.7	4	0.08869	0.07958	0.00102	0.16844	[20]	Samples collected from fishermen at town along River Offin in Feb. and Jul. 2006
Dunkwa-on-Offin, Upper Denkyira East Municipal District, Central Region	2006	d/w	Heterobranchus sp.	Catfish	F	3.2 - 3.7	1	0.06572	-	-	-	[20]	Samples collected from fishermen at town along River Offin in Feb. and Jul. 2006
Obuasi, Ashanti Region	1992 - 1993	w/d	Heterobranchus bidorsalis	African catfish; Eel-like fattyfin catfish; Mudfish	F	3.7	33	0.131	0.114	0.040	0.400	[7]	Samples collected once a month from Mar to Feb from 14 villages (3 at each site) around the Pompora Treatment Plant at Obuasi.
River Pra Basin, Western, Central, and Ashanti Regions	2005 - 2006	w/d	Heterobranchus longifillis (alt. spelling: Heterobranchus longifilis)	Sampa	F	3.7	3	0.165	-	0.142	0.187	[9]	Samples collected from Twifo Praso between Aug. 2005 to Jan. 2006 from fishermen.
River Pra Basin, Western, Central, and Ashanti Regions	2005 - 2006	w/d	Alestes imberi (synonymn:Brycinus imberi)	Characin, spot-tail tetra/robber	F	3.3	53	0.049	0.014	0.014	0.216	[9]	Samples collected from Twifo Praso, Twifo Mampong, and Beposo between Aug. 2005 to Jan. 2006 from fishermen.
Kpong Reservoir, Eastern Region	2004 - 2005	d/w	Apistogramma trifasciata	Three-striped apisto (cichlid)	F	3.3	3	0.011	-	0.010	0.011	[33]	Samples collected from commercial catches from fisherman in villages along the reservoir from Feb. through Dec.
Buabenso, Ashanti Region	2006	d/w	Schilbe mystus	African butter catfish	F	3.3	2	0.10902	0.00524	0.10532	0.11273	[20]	Samples collected from fishermen at town along River Offin in Feb. and Jul. 2006
River Pra Basin, Western, Central, and Ashanti Regions	2005 - 2006	w/d	Chrysichthys sp.	Catfish	F	3.4[a]	28	0.169	0.017	0.069	0.370	[9]	Samples collected from Twifo Praso, Beposo, and Daboase between Aug. 2005 to Jan. 2006 from fishermen.

ND = Not detectable.
a Trophic level from Oppong et al. 2010 [9].
b Trophic level is for *Clarias anguilaris* for Donkor et al., 2006 [8].
c All other trophic levels from FishBase [40].

Table S5. Total mercury (Hg) levels in fish and shellfish in Ghana and the Gulf of Guinea. Hg concentrations are presented in wet weight (dry weight values were converted to w.w. assuming 80% moisture [32]).

Study location/ Sample collection	Year collected	Dry/Wet Season	Species	Common name(s)	Fresh or Marine	Trophic Level[c]	Sample size (n)	Mean Hg (μg/g)	SD Hg (μg/g)	Min. Hg (μg/g)	Max. Hg (μg/g)	Reference	Notes
River Pra Basin, Western, Central, and Ashanti Regions	2003	d	Clarias sp.	Catfish, Mudfish	F	3.4[b]	2	0.208	--	--	--	[8]	Dry fish samples were purchased from local fisherman during Janurary 2003 (dry season).
Dunkwa-on-Offin, Upper Denkyira East Municipal District, Central Region	2006	d/w	Papyrocranus afer	Reticulate knifefish	F	3.6	1	0.05329	-	-	-	[20]	Samples collected from fishermen at town along River Offin in Feb. and Jul. 2006
Upper Volta Basin at Yeji, Brong-Ahafo Region	2006 - 2007	d	Bagrus docmak	Sudan catfish; silver catfish; Semutundu	F	4.1	19	0.06703	-	0.04080	0.09030	[31]	Samples were collected from commercial catches at Yeji from Oct. 2006 to Mar. 2007.
River Pra Basin, Western, Central, and Ashanti Regions	2003	d	Hepsetus odoe	Kafue pike, African pike, Pike characid	F	4.5	2	0.895	--	--	--	[8]	Dry fish samples were purchased from local fisherman during Janurary 2003 (dry season).
Dunkwa-on-Offin, Upper Denkyira East Municipal District, Central Region	2006	d/w	Hepsetus odoe	Kafue pike, African pike, Pike characid	F	4.5	2	0.58704	0.29352	0.37950	0.97459	[20]	Samples collected from fishermen at town along River Offin in Feb. and Jul. 2006
Dunkwa-on-Offin, Upper Denkyira East Municipal District, Central Region	2006	d/w	Mormyrus sp.	Elephantfish	F	-	1	0.04681	-	-	-	[20]	Samples collected from fishermen at town along River Offin in Feb. and Jul. 2006
Kejetia ASG mine, Talensi District, Upper East Region	2010	w	unknown	unknown	F	-	12	0.0141	0.0115	0.0049	0.0444	[10]	Samples collected from streams and reservoirs surrounding ASGM communities. Dry weight was converted using 80% moisture assumption.
Gulf of Guinea	2004	d	Auxis thazard thazard	Frigate tuna	M	4.3	21	0.108	-	0.044	0.201	[34]	Samples collected from commercial catches at the Tema Fishing Harbour in Apr. 2004.
Jamestown, Accra, Greater Accra Region	2003 - 2004	d	Auxis thazard thazard	Frigate tuna	M	4.3	1	0.117	-	-	-	[35]	Samples collected from commercial catches at the James Town fishing port in Accra between Nov. 2003 and Feb. 2004.
Gulf of Guinea	2004	w	Auxis thazard thazard	Frigate tuna	M	4.3	9	0.112	0.044	0.053	0.197	[36]	Samples collected from commercial catches from the Tema Fishing Harbour in May 2004.
Jamestown, Accra, Greater Accra Region	2003 - 2004	d	Brachydeuterus auritus	Bigeye grunt	M	3	5	0.037	0.017	-	-	[37]	Samples collected from commercial catches at James Town, Accra between Nov. 2003 and Jan. 2004.
Jamestown, Accra, Greater Accra Region	2003 - 2004	d	Caranx crysos	Blue runner	M	4.4	3	0.035	-	0.026	0.041	[35]	Samples collected from commercial catches at the James Town fishing port in Accra between Nov. 2003 and Feb. 2004.
Jamestown, Accra, Greater Accra Region	2003 - 2004	d	Caranx hippos	Crevalle jack	M	3.5	3	0.063	-	0.050	0.072	[35]	Samples collected from commercial catches at the James Town fishing port in Accra between Nov. 2003 and Feb. 2004.
Jamestown, Accra, Greater Accra Region	2003 - 2004	d	Chloroscombrus chrysurus	Atlantic bumper	M	3.2	3	0.112	-	0.094	0.129	[35]	Samples collected from commercial catches at the James Town fishing port in Accra between Nov. 2003 and Feb. 2004.

ND = Not detectable.
a Trophic level from Oppong et al. 2010 [9].
b Trophic level is for *Clarias anguilaris* for Donkor et al., 2006 [8].
c All other trophic levels from FishBase [40].

Table S5. Total mercury (Hg) levels in fish and shellfish in Ghana and the Gulf of Guinea. Hg concentrations are presented in wet weight (dry weight values were converted to w.w. assuming 80% moisture [32]).

Study location/ Sample collection	Year collected	Dry/Wet Season	Species	Common name(s)	Fresh or Marine	Trophic Level[c]	Sample size (n)	Mean Hg (µg/g)	SD Hg (µg/g)	Min. Hg (µg/g)	Max. Hg (µg/g)	Reference	Notes
Jamestown, Accra, Greater Accra Region	2003 - 2004	d	Cynoglossus cadenati	Ghanaian tonguesole	M	3.4	3	0.033	-	0.027	0.042	[35]	Samples collected from commercial catches at the James Town fishing port in Accra between Nov. 2003 and Feb. 2004.
Jamestown, Accra, Greater Accra Region	2003 - 2004	d	Decapterus punctatus	Round scad	M	4.3	2	0.009	-	0.008	0.010	[35]	Samples collected from commercial catches at the James Town fishing port in Accra between Nov. 2003 and Feb. 2004.
Jamestown, Accra, Greater Accra Region	2003 - 2004	d	Decapterus rhonchus (synonym: Caranx rhonchus)	False scad	M	3.6	5	0.430	0.020	-	-	[37]	Samples collected from commercial catches at James Town, Accra between Nov. 2003 and Jan. 2004.
Jamestown, Accra, Greater Accra Region	2003 - 2004	d	Dentex canariensis	Canary porgy, Canary dentex	M	3.6	2	0.099	-	0.075	0.122	[35]	Samples collected from commercial catches at the James Town fishing port in Accra between Nov. 2003 and Feb. 2004.
Jamestown, Accra, Greater Accra Region	2003 - 2004	d	Diplodus puntazzo	Sharpsnout seabream	M	2.9	4	0.070	0.013	-	-	[37]	Samples collected from commercial catches at James Town, Accra between Nov. 2003 and Jan. 2004.
Jamestown, Accra, Greater Accra Region	2003 - 2004	d	Galeoides decadactylus	Guinean threadfin, Lesser African threadfin	M	3.6	5	0.041	0.020	-	-	[37]	Samples collected from commercial catches at James Town, Accra between Nov. 2003 and Jan. 2004.
Jamestown, Accra, Greater Accra Region	2003 - 2004	d	Galeoides decadactylus	Guinean threadfin, Lesser African threadfin	M	3.6	4	0.041	-	0.024	0.065	[35]	Samples collected from commercial catches at the James Town fishing port in Accra between Nov. 2003 and Feb. 2004.
Jamestown, Accra, Greater Accra Region	2004 - 2004	d	Gerres nigri	Guinean striped mojarra	M	3.2	5	0.056	0.024	-	-	[37]	Samples collected from commercial catches at James Town, Accra between Nov. 2003 and Jan. 2004.
Jamestown, Accra, Greater Accra Region	2003 - 2004	d	Hemiramphus brasiliensis	Ballyhoo, Ballyhoo halfbeak	M	2.5	3	0.022	-	0.016	0.031	[35]	Samples collected from commercial catches at the James Town fishing port in Accra between Nov. 2003 and Feb. 2004.
Jamestown, Accra, Greater Accra Region	2003 - 2004	d	Lagocephalus lagocephalus	Oceanic puffer	M	3.7	4	0.066	0.023	-	-	[37]	Samples collected from commercial catches at James Town, Accra between Nov. 2003 and Jan. 2004.
Jamestown, Accra, Greater Accra Region	2003 - 2004	d	Parapristipoma humile	Guinean grunt	M	3.6	3	0.122	0.021	-	-	[37]	Samples collected from commercial catches at James Town, Accra between Nov. 2003 and Jan. 2004.
Jamestown, Accra, Greater Accra Region	2003 - 2004	d	Pomatomus saltatrix	Bluefish	M	4.5	1	0.126	-	-	-	[35]	Samples collected from commercial catches at the James Town fishing port in Accra between Nov. 2003 and Feb. 2004.
Jamestown, Accra, Greater Accra Region	2003 - 2004	d	Pseudotolithus senegalensis	Captainfish, Cassava croaker	M	3.8	3	0.031	0.025	-	-	[37]	Samples collected from commercial catches at James Town, Accra between Nov. 2003 and Jan. 2004.

ND = Not detectable.
a Trophic level from Oppong et al. 2010 [9].
b Trophic level is for *Clarias anguilaris* for Donkor et al., 2006 [8].
c All other trophic levels from FishBase [40].

Table S5. Total mercury (Hg) levels in fish and shellfish in Ghana and the Gulf of Guinea. Hg concentrations are presented in wet weight (dry weight values were converted to w.w. assuming 80% moisture [32]).

Study location/ Sample collection	Year collected	Dry/Wet Season	Species	Common name(s)	Fresh or Marine	Trophic Level[c]	Sample size (n)	Mean Hg (µg/g)	SD Hg (µg/g)	Min. Hg (µg/g)	Max. Hg (µg/g)	Reference	Notes
Jamestown, Accra, Greater Accra Region	2003 - 2004	d	Pseudotolithus senegalensis	Captainfish, Cassava croaker	M	3.8	3	0.031	-	0.010	0.059	[35]	Samples collected from commercial catches at the James Town fishing port in Accra between Nov. 2003 and Feb. 2004.
Jamestown, Accra, Greater Accra Region	2003 - 2004	d	Pseudotolithus typus	Flathead captainfish, Longneck croaker	M	3.7	3	0.160	-	0.116	0.191	[35]	Samples collected from commercial catches at the James Town fishing port in Accra between Nov. 2003 and Feb. 2004.
Jamestown, Accra, Greater Accra Region	2003 - 2004	d	Pseudupeneus prayensis	West African goatfish	M	3.2	3	0.081	-	0.054	0.097	[35]	Samples collected from commercial catches at the James Town fishing port in Accra between Nov. 2003 and Feb. 2004.
Jamestown, Accra, Greater Accra Region	2003 - 2004	d	Sardinella aurita	Spanish sardine, Round sardinella	M	3.4	3	0.009	-	0.007	0.012	[35]	Samples collected from commercial catches at the James Town fishing port in Accra between Nov. 2003 and Feb. 2004.
Jamestown, Accra, Greater Accra Region	2003 - 2004	d	Scomber japonicus	Chub mackerel, Pacific chub mackerel	M	3.1	2	0.041	-	0.037	0.045	[35]	Samples collected from commercial catches at the James Town fishing port in Accra between Nov. 2003 and Feb. 2004.
Jamestown, Accra, Greater Accra Region	2003 - 2004	d	Selene dorsalis	Guinean moonfish, African moonfish	M	4.1	5	0.034	-	0.014	0.061	[35]	Samples collected from commercial catches at the James Town fishing port in Accra between Nov. 2003 and Feb. 2004.
Jamestown, Accra, Greater Accra Region	2003 - 2004	d	Selene dorsalis	Guinean moonfish, African moonfish	M	4.1	5	0.034	0.023	-	-	[37]	Samples collected from commercial catches at James Town, Accra between Nov. 2003 and Jan. 2004.
Jamestown, Accra, Greater Accra Region	2003 - 2004	d	Sphyraena guachancho	Guachanche barracuda	M	3.9	3	0.013	-	0.009	0.015	[35]	Samples collected from commercial catches at the James Town fishing port in Accra between Nov. 2003 and Feb. 2004.
Jamestown, Accra, Greater Accra Region	2003 - 2004	d	Stromatteus fiatola	Blue butterfish	M	4	4	0.004	0.003	-	-	[37]	Samples collected from commercial catches at James Town, Accra between Nov. 2003 and Jan. 2004.
Gulf of Guinea	2004	w	Thunnus albacares	Yellowfin tuna	M	4.3	11	0.061	0.021	0.036	0.090	[36]	Samples collected from commercial catches from the Tema Fishing Harbour in May 2004.
Jamestown, Accra, Greater Accra Region	2003 - 2004	d	Trachinotus goreensis	Longfin pompano	M	3.7	3	0.027	-	0.012	0.049	[35]	Samples collected from commercial catches at the James Town fishing port in Accra between Nov. 2003 and Feb. 2004.
Jamestown, Accra, Greater Accra Region	2003 - 2004	d	Trachurus trecae	Smallscale scad, Cunene horse mackerel	M	3.5	2	0.092	-	0.087	0.097	[35]	Samples collected from commercial catches at the James Town fishing port in Accra between Nov. 2003 and Feb. 2004.
Jamestown, Accra, Greater Accra Region	2003 - 2004	d	Trichiurus lepturus	Atlantic cutlassfish, Largehead hairtail	M	4.5	3	0.013	-	0.009	0.017	[35]	Samples collected from commercial catches at the James Town fishing port in Accra between Nov. 2003 and Feb. 2004.

ND = Not detectable.
a Trophic level from Oppong et al. 2010 [9].
b Trophic level is for *Clarias anguilaris* for Donkor et al., 2006 [8].
c All other trophic levels from FishBase [40].

Table S5. Total mercury (Hg) levels in fish and shellfish in Ghana and the Gulf of Guinea. Hg concentrations are presented in wet weight (dry weight values were converted to w.w. assuming 80% moisture [32]).

Study location/ Sample collection	Year collected	Dry/Wet Season	Species	Common name(s)	Fresh or Marine	Trophic Level[c]	Sample size (n)	Mean Hg (µg/g)	SD Hg (µg/g)	Min. Hg (µg/g)	Max. Hg (µg/g)	Reference	Notes
Shellfish													
Ada and Aveglo, Volta Estuary, Volta Region	2008-2009	d/w	Galatea paradoxa	Clam	F	-	-	-	-	0.006	0.015	[23]	Samples were collected from fisherman at two sample sites during monthly intervals between March 2008 and August 2009.
Sakumo Lagoon, Greater Accra Region	1997	d	Anadara senilis (synonym: Senilia senilis)	Clam	M	-	30	0.032 (median)	-	0.02	0.09	[38]	Samples collected in Feb. 1997.
Sakumo Lagoon, Greater Accra Region	1996	w	Anadara senilis (synonym: Senilia senilis)	Clam	M	-	38	0.022 (median)	-	0.01	0.08	[38]	Samples collected in Oct. 1996.
Benya and Ningo Lagoons, Greater Accra Region	1997	d	Anadara senilis (synonym: Senilia senilis)	Clam	M	-	81	0.066 (median)	-	0.03	0.18	[38]	Samples collected in Feb. 1997.
Benya and Ningo Lagoons, Greater Accra Region	1996	w	Anadara senilis (synonym: Senilia senilis)	Clam	M	-	45	0.038 (median)	-	0.02	0.07	[38]	Samples collected in Oct. 1996.
Jamestown, Accra, Greater Accra Region	2003 - 2004	d	Calappa rubroguttata	Crab	M	-	5	0.057	0.022	-	-	[37]	Samples collected from commercial catches at James Town, Accra between Nov. 2003 and Jan. 2004.
Jamestown, Accra, Greater Accra Region	2003 - 2004	d	Panulirus argus	Caribbean spiny lobster	M	-	5	0.035	0.015	-	-	[37]	Samples collected from commercial catches at James Town, Accra between Nov. 2003 and Jan. 2004.
Benya Lagoon, Greater Accra Region	1996	w	Perna perna	Brown mussel	M	-	30	0.40 (median)	--	0.02	0.06	[39]	Data from samples collected in the wet and dry seasons at Benya Lagoon (open).
Benya Lagoon, Greater Accra Region	1997	d	Perna perna	Brown mussel	M	-	30	0.74 (median)	--	0.04	0.13	[39]	Data from samples collected in the wet and dry seasons at Benya Lagoon (open).
Sakumo Lagoon, Greater Accra Region	1996	w	Perna perna	Brown mussel	M	-	18	0.052 (median)	--	0.03	0.15	[39]	Data from samples collected in the wet and dry seasons at Sakuma Lagoon (closed).
Sakumo Lagoon, Greater Accra Region	1997	d	Perna perna	Brown mussel	M	-	19	0.066 (median)	--	0.04	0.11	[39]	Data from samples collected in the wet and dry seasons at Sakuma Lagoon (closed).
Benya Lagoon, Greater Accra Region	1996	w	Crassostrea tulipa	Mangrove oyster	M	-	24	0.028 (median)	--	0.02	0.06	[39]	Data from samples collected in the wet and dry seasons at Benya Lagoon (open).
Benya Lagoon, Greater Accra Region	1997	d	Crassostrea tulipa	Mangrove oyster	M	-	59	0.042 (median)	--	0.02	0.09	[39]	Data from samples collected in the wet and dry seasons at Benya Lagoon (open).
Ningo Lagoon, Greater Accra Region	1996	w	Crassostrea tulipa	Mangrove oyster	M	-	12	0.026 (median)	--	0.02	0.03	[39]	Data from samples collected in the wet and dry seasons at Ningo Lagoon (open).
Ningo Lagoon, Greater Accra Region	1997	d	Crassostrea tulipa	Mangrove oyster	M	-	31	0.032 (median)	--	0.01	0.05	[39]	Data from samples collected in the wet and dry seasons at Ningo Lagoon (open).
Sakumo Lagoon, Greater Accra Region	1996	w	Crassostrea tulipa	Mangrove oyster	M	-	55	0.024 (median)	--	0.01	0.05	[39]	Data from samples collected in the wet and dry seasons at Sakuma Lagoon (closed).
Sakumo Lagoon, Greater Accra Region	1997	d	Crassostrea tulipa	Mangrove oyster	M	-	25	0.026 (median)	--	0.02	0.04	[39]	Data from samples collected in the wet and dry seasons at Sakuma Lagoon (closed).

ND = Not detectable.
a Trophic level from Oppong et al. 2010 [9].
b Trophic level is for *Clarias anguilaris* for Donkor et al., 2006 [8].
c All other trophic levels from FishBase [40].

Table S6. Total mercury (Hg) levels in lichen and seaweed bioindicators and tailings in Ghana and from the Gulf of Guinea.

Study location	Year collected	Dry/wet season	Media / species name	Sample size (n)	Mean Hg (μg/g)	SD Hg (μg/g)	Min. Hg (μg/g)	Max. Hg (μg/g)	Reference	Notes
LICHENS & SEAWEED (BIOINDICATORS)										
Tarkwa, Wassa West District, Western Region	2008-2009	d	Lichens: Parmelia sulcat	50	-	-	0.16	1.24	[41]	In situ lichens were sampled once per month from Sep. to Jan. from 10 sampling points from the barks of trees, 1.5-2 m above the ground.
Obuasi, Ashanti Region	2002-2003	w	Lichens: Parmelia	-	3.5	-	1.2	5.2	[42]	In situ lichens were collected from June 2002 to Aug. 2003 from tree barks, at least 1.0 m above the ground.
Prestea, Western Region	2002-2003	w	Lichens: Parmelia	-	6.2	-	1	14.5	[42]	In situ lichens were collected from June 2002 to Aug. 2003 from tree barks, at least 1.0 m above the ground.
Reference sites: Non-mining locations										
Afianya & Doryum, Greater Accra Region	2002-2003	w	Lichens: Parmelia	-	0.01	-	-	-	[42]	In situ lichens were collected from June 2002 to Aug. 2003 from tree barks, at least 1.0 m above the ground. Afianya & Doryum are industrial areas.
Samreboi, Greater Accra Region	2002-2003	w	Lichens: Parmelia	-	0.78	-	0.5	1.4	[42]	In situ lichens were collected from June 2002 to Aug. 2003 from tree barks, at least 1.0 m above the ground. Samreboi and Akim Tafo are farming areas.
Akim Tafo, Greater Accra Region	2002-2003	w	Lichens: Parmelia	-	2.2	-	0.55	4.5	[42]	In situ lichens were collected from June 2002 to Aug. 2003 from tree barks, at least 1.0 m above the ground. Samreboi and Akim Tafo are farming areas.
University of Cape Coast, Central Region	2008-2009	d	Lichens: Parmelia sulcata	5	ND	-	-	-	[41]	In situ lichens were sampled once per month from Sep. to Jan. from 1 sampling point from the barks of trees, 1.5-2 m above the ground.
Prampram, Nungua, Winneba, Cape Coast, Sekondi and Axim beaches, Gulf of Guinea	1996-1998	d/w	Seaweed: Enteromorpha flexuosa	8*	ND	-	-	-	[43]	Samples collected bimonthly from rocky shores on beaches, at 2 sites 8 times.
		d/w	Seaweed: Ulva fasciata	8*	0.13	0.02	-	-		Samples collected bimonthly from rocky shores on beaches, at 3 sites 5 times and 6 sites 3 times.
		d/w	Seaweed: Chaetomorpha antennina	6	ND	-	-	-		Samples collected bimonthly from rocky shores on beaches, at 1 site 6 times.
		d/w	Seaweed: Chaetomorpha linum	8*	0.31	0.09	-	-		Samples collected bimonthly from rocky shores on beaches, at 1 site 4 times, and at 4 sites 4 times.
		d/w	Seaweed: Caulerpa taxifolia	7	0.65	0.01	-	-		Samples collected bimonthly from rocky shores on beaches, at 1 site 7 times.
		d/w	Seaweed: Bachelotia antillarium	6*	0.052	0.003	-	-		Samples collected bimonthly from rocky shores on beaches, at 1 site 2 times and at 2 sites 4 times.
		d/w	Seaweed: Padina durvilliae	3*	0.054	0.007	-	-		Samples collected bimonthly from rocky shores on beaches, at 2 sites 1 time, and at 4 sites 2 times.
		d/w	Seaweed: Sargassum vulgare	13*	0.02	0.002	-	-		Samples collected bimonthly from rocky shores on beaches, at 3 sites 7 times, and at 4 sites 6 times.

ND = Not detectable or below the detection limit.

Table S6. Total mercury (Hg) levels in lichen and seaweed bioindicators and tailings in Ghana and from the Gulf of Guinea.

Study location	Year collected	Dry/wet season	Media / species name	Sample size (n)	Mean Hg (µg/g)	SD Hg (µg/g)	Min. Hg (µg/g)	Max. Hg (µg/g)	Reference	Notes
Prampram, Nungua, Winneba, Cape Coast, Sekondi, and Axim beaches, Gulf of Guinea	1996-1998	d/w	Seaweed: Polycavernousa dentata	13*	0.100	0.008	-	-	[44]	Samples collected bimonthly from rocky shores on beaches, at 3 sites 7 times and 6 sites 6 times.
		d/w	Seaweed: Gigartina acicularis	6	0.058	0.006	-	-		Samples collected bimonthly from rocky shores on beaches, at 1 site 6 times.
		d/w	Seaweed: Centroceras clavulatum	5	0.220	0.009	-	-		Samples collected bimonthly from rocky shores on beaches, at 1 site 5 times.
		d/w	Seaweed: Bryoclidia thysigera	8*	ND	-	-	-		Samples collected bimonthly from rocky shores on beaches, at 1 site 3 times and 2 sites 5 times.
		d/w	Seaweed: Jania rubens	4	ND	-	-	-		Samples collected bimonthly from rocky shores on beaches, at 1 site 4 times.
		d/w	Seaweed: Hypnea musciformis	4*	0.11	0.007	-	-		Samples collected bimonthly from rocky shores on beaches, at 2 sites 4 times.

*sample sizes may be greater than listed

TAILINGS										
River Ankroba Basin, Wassa West District, Western Region	666	-	Tailings	6	0.795	1.155	0.033	2.500	[17]	Old mine tailing dumps. Samples were collected in the mining towns of Nsuta, Tarkwa, Damang, Abosso, Bogoso, and Prestea, in a tropical rainforest corridor. Large, small-scale and illegal mining ocurrs in the area.
River Pra Basin; Western, Central, and Ashanti Regions	2002	w	Sediment tailings	3	0.079	0.050	0.032	0.132	[8]	Tailings in sediments. Samples taken from the Rivers Offin, Upper Pra and Lower Pra, once in the wet & dry seasons each.
	2002	d	Sediment tailings	3	0.054	0.057	0.008	0.118		
Dumasi Area (sediment from a galamsay tailing pond that discharges into Apopre river), Wassa West District, Western Region	2001	-	Sediment tailings	2	19.296	-	-	-	[1]	Data has been extracted from a book chapter(12) that was contributed to by the authors of the article
River Pra Basin; Western, Central, and Ashanti Regions	2002	w	Soil tailings	3	1.642	2.800	0.015	4.876	[8]	Tailings in soil. Samples taken from the Rivers Offin, Upper Pra and Lower Pra, once in the wet & dry seasons each.
	2003	d	Soil tailings	2	0.019	0.013	0.010	0.028		
Bibiani District, Western Region	2007	w	Tailings	4	0.011	0.006	0.007	0.020	[6]	Tailings from tailings dams of the Central African Goldmine. Samples collected in Sep. Samples collected from 0 to 15-20 cm depth. Two sites recorded levels below the detection limit (0.01 µg/g)
Bibiani District, Western Region	2008 - 2009	d	Tailings	4	0.373	0.301	0.102	0.786	[21]	Duplicate samples collected from surface, 20, 40, and 60cm depths at Bibiani north galamsey tailings dump (old) during the dry season (Oct - Mar).

ND = Not detectable or below the detection limit.

Table S6. Total mercury (Hg) levels in lichen and seaweed bioindicators and tailings in Ghana and from the Gulf of Guinea.

Study location	Year collected	Dry/wet season	Media / species name	Sample size (n)	Mean Hg (μg/g)	SD Hg (μg/g)	Min. Hg (μg/g)	Max. Hg (μg/g)	Reference	Notes
Bibiani, Anwiaso, Bekwai District, Western Region	2008 -2009	d	Tailings	4	2.643	2.213	0.472	4.998	[21]	Duplicate samples collected from surface, 20, 40, and 60cm depths at galamsey tailings dump (current) during the dry season (Oct - Mar).
Bibiani, Anwiaso, Bekwai District; Western Region	2008 - 2009	d	Tailings	-	1.739	-	-	-	[18]	Samples collected from waterway in the dry season from Oct 2008 to Mar 2009 a galamsey tailing site. Surface sediments (0 to ~10 cm) were taken where water samples were also collected (~60 samples total for entire study).
Bibiani, Anwiaso, Bekwai District, Western Region	2008 - 2009	d	Tailings	4	0.898	0.133	0.721	1.024	[21]	Duplicate samples collected from surface, 20, 40, and 60cm depths at abandoned galamesey tailings dump during the dry season (Oct - Mar).
Kejetia ASG mine, Talensi District, Upper East Region	2011	w	Tailings	1	0.4203	-	-	-	[10]	Tailing collected from a sluicing pond in June 2011.

ND = Not detectable or below the detection limit.

Table S7. Total arsenic (As) levels in soils near Ghanaian small-scale mining sites and reference non-mining sites.

Study Location	Year collected	Dry/wet season	Sample size (n)	Mean As (ng/g)	SD As (ng/g)	Min. As (ng/g)	Max. As (ng/g)	Reference	Notes
Obuasi, Ashanti Region	1992-1993	w/d	168	12900	17500	2100	48900	[7]	Samples collected once a month (Mar 1992 - Feb 1993) from from 14 sites located 0.31 - 17.5 km from Pompora (water) Treatment Plant. Both large and small-scale mining occurs nearby. Three samples per site.
Wassa West, Western Region	-	-	5	13360	970.6	12280	14840	[4]	Samples collected from farms at 5 gold mining towns (Iduapreim, Odumasi, Hemang, Huni Valley, Damang).
Abosso Goldfield Ltd.-Daamang, Western Region	-	d	36	1800	-	-	-	[45]	Samples collected at 10cm depth from a variety of soil sites at each mine (Goldfields Ghana Limited - Tarkwa, Abosso Goldfields Limited - Daamang, Anglogold Ashanti - Iduapriem, Bogosu Gold Limited), including topsoil, waste dumps, tailings, and undisturbed vegetation.
Bogosu Gold Ltd., Western Region			20	227000	-	-	-		
Anglogold Ashanti-Iduapriem Mine, Western Region			24	1100	-	-	-		
Goldfields Ghana Ltd.-Tarkwa, Western Region			42	1900	-	-	-		
Reference sites: Non-mining locations									
Abonse, Eastern Region	2002	w	40	2050	390	1620	2540	[46]	Sampling done at 3-month intervals (Mar to Dec), at a depth of 10-20 cm
Mamfe, Eastern Region			40	1080	220	850	1360		
Pepease, Eastern Region			40	3860	1330	2130	5190		
Otsew, Gomoa West District, Central Region	2010	d	1	6730	100	-	-	[11]	Sample of earthenware clay soil was purchased from a market in November 2010.
Otsew, Gomoa West District, Central Region	2010	d	10	9480	-	ND	18470	[11]	Samples from 10 sites for earthenware clay soil deposits from 30cm depth in November 2010.
Wassa West, Western Region	-	-	3	7403	487.6	6850	7770	[4]	Samples collected from farms at 3 non-mining towns (Ayanfuri, Wassa Asikuma, and Krofofrom).
Duabone and Nwowam, Brong-Ahafo Region	-	-	6	100	-	-	-	[45]	3 to 15 random samples of paddy soil samples were collected in inland valley or irrigated paddy fields at a 10cm depth.
Ejura, Kobriti, Biemso (at 2 sites), Odumase, and Nobewam, Ashanti Region		-	27	10500	-	-	-		
Assin Dansame and Assin Akropong, Central Region		-	9	700	-	-	-		
Abenase, Oda Nkwanta, and Kpong, Eastern Region		-	36	700	-	-	-		
Sefwi Asafo, Sefwi Anyinabrim, Mempeasem, Aduku Tanoso, Yirase, Simpa Krutu/Anikorkor, and Kobina Anokrom, Western Region		-	42	1300	-	-	-		

ND = Not detectable or below the detection limit.

Table S8. Total arsenic (As) levels in edible plants near Ghanaian small-scale mining sites.

Study Location	Year collected	Dry/wet season	Species (common name)	Sample size (n)	Mean As (μg/g)	SD As (μg/g)	Min. As (μg/g)	Max. As (μg/g)	Reference	Notes
Wassa West District, Western Region	-	-	Manihot esculentus (cassava)	8	6.87	2.89	2.98	9.84	[4]	Samples collected from farms at 5 gold mining towns (Iduapreim, Odumasi, Hemang, Huni Valley, Damang) and 3 non-mining towns (Ayanfuri, Wassa Asikuma, Krofofrom).
Wassa West District, Western Region	-	-	Xanthosoma sagittifolium (cocoyam)	8	6.065	2.478	2.74	8.51	[4]	Samples collected from farms at 5 gold mining towns (Iduapreim, Odumasi, Hemang, Huni Valley, Damang) and 3 non-mining towns (Ayanfuri, Wassa Asikuma, Krofofrom).
Wassa West District, Western Region	-	-	Colocasia esculenta (water cocoyam)	8	6.411	2.202	3.69	-	[4]	Samples collected from farms at 5 gold mining towns (Iduapreim, Odumasi, Hemang, Huni Valley, Damang) and 3 non-mining towns (Ayanfuri, Wassa Asikuma, Krofofrom).
Wassa West District, Western Region	-	-	Musa paradisiacal (plantain)	8	6.014	2.004	3.12	8.43	[4]	Samples collected from farms at 5 gold mining towns (Iduapreim, Odumasi, Hemang, Huni Valley, Damang) and 3 non-mining towns (Ayanfuri, Wassa Asikuma, Krofofrom).
Obuasi, Ashanti Region	1992-1993	w/d	Musa paradisiacal (plantain)	156	1.862	-	0.5	4.3	[7]	Samples collected once a month from Mar to Feb from 13 villages around the Pompora Treatment Plant at Obuasi (3 samples per site).
Obuasi, Ashanti Region	1992-1993	w/d	Ceratopteris cornuta (fern)	168	24.05	-	2.1	78.7	[7]	Samples collected once a month from Mar to Feb from 14 villages around the Pompora Treatment Plant at Obuasi (3 samples per site).
Obuasi, Ashanti Region	1992-1993	w/d	Pennisetum purpureum (elephant grass)	120	6.72	-	1.3	27.4	[7]	Samples collected once a month from Mar to Feb from 10 villages around the Pompora Treatment Plant at Obuasi (3 samples per site).
Obuasi, Ashanti Region	1992-1993	w/d	Manihot esculenta (cassava)	144	1.283	-	0.7	2.6	[7]	Samples collected once a month from Mar to Feb from 12 villages around the Pompora Treatment Plant at Obuasi (3 samples per site).
Efuantah, Tarkwa, Western Region	-	-	Xanthosoma sagititolium (cocoyam)	1	146	-	-	-	[12]	Samples collected from a mining town (Efuantah)
Nsuta and Tamso, Tarkwa, Western Region	-	-	Colocasia esculenta (water cocoyam)	2	383.5	24.7	366	401	[12]	Samples collected from 2 mining towns (Nsuta, and Tamso)

Table S9. Total arsenic (As) levels in sediment near Ghanaian small-scale mining sites and reference non-mining sites.

Study Location	Year collected	Dry/wet season	Sample size (n)	Mean As (ng/g)	SD As (ng/g)	Min. As (ng/g)	Max. As (ng/g)	Reference	Notes
River Ankobra, Western Region	2001-2002	w/d	-	61800	92700	1150	183,000	[16]	Samples collected in Sep. 2001 and Feb. 2002 from five ares on the River Ankobra: Ankwaso, Prestea, Dombase (all on the main Ankobra River), Beppo (Manse tributary) and Bonsaso (Bonsa tributary), and one area at Anwiaso (10km from the source).
Mining area, River Pra Basin; Western, Ashanti, Central Regions	2002-2003	d	14	684	1291	88	5076	[47]	14 sampling sites along the River Pra (2 downstream--1 past ASGM--and 12 upstream), collected during the wet season of 2002 and dry season of 2003.
		w	14	322	215	79	714		
Reference sites: Non-mining locations									
Non-mining area, River Pra Basin; Western, Central, Ashanti Regions	2002-2003	d	7	230	108	103	371	[47]	7 sites from non-mining areas along the River Pra (5 are from downstream and 2 upstream), collected during the wet season of 2002 and dry season of 2003.
		w	7	266	132	125	414		
Shama, Western Region (Pra); and Elmina (Benya), Cape Coast (Fosu), and Saltpong Beach (Narkwa), Central Region, Gulf of Guinea	-	w	32	311.1	439.7	0	1432.0	[22]	Samples collected from Pra Estuary, Benya Lagoon, Fosu Lagoon, and Narkwa Lagoon during wet and dry season.
Shama, Western Region (Pra); and Elmina (Benya), Cape Coast (Fosu), and Saltpong Beach (Narkwa), Central Region, Gulf of Guinea	-	d	32	80.6	222.3	0	910.0		
River Subin, Kumasi, Ashanti Region	2005	d/w	10	-	-	2340	7650	[48]	Samples were collected bi-monthly form Jan. to Sep. 2005

Table S10. Total arsenic (As) levels in water near Ghanaian small-scale mining sites and reference non-mining sites.

Study Location	Year collected	Dry/wet season	Water type	Water source	Sample Size (n)	Mean As (µg/L)	SD As (µg/L)	Min. As (µg/L)	Max. As (µg/L)	Reference	Notes
Tarkwa, Western Region	2004	d	Groundwater	Borehole	12	1.3	1.1	0.5	4	[24]	Drinking water samples taken from around Tarkwa in March 2004.
Tarkwa, Western Region	2004	d	Groundwater	Well	6	0.2	0.4	0.07	1	[24]	Drinking water samples taken from around Tarkwa in March 2004.
Tereribe, Anglogold Ashanti-Iduapriem Mine, Tarkwa, Western Region	2010	d	Groundwater	Boreholes	3	330	-	< 1	860	[49]	Samples collected in March 2010 from boreholes.
Mile 7, Anglogold Ashanti-Iduapriem Mine, Tarkwa, Western Region	2010	d	Groundwater	Boreholes	2	310	-	80	530	[49]	Samples collected in March 2010 from boreholes.
Abekoase, Gold Fields Ghana Ltd., Tarkwa, Western Region	2010	d	Groundwater	Boreholes	5	270	-	< 1	960	[49]	Samples collected in March 2010 from boreholes.
Huniso, Gold Fields Ghana Ltd., Tarkwa, Western Region	2010	d	Groundwater	Boreholes	2	20	-	< 1	40	[49]	Samples collected in March 2010 from boreholes.
Dumasi, Wassa West District, Western Region	2004-2005	d/w	Groundwater	Boreholes	160	5390	860.0	4520	6521	[50]	Samples were collected from 4 boreholes (40 samples at each borehole) between Aug. 2004 and Jun. 2005. Values here are the means from each borehole.
Obuasi, Ashanti Region	2008	-	Groundwater	Tap	8	1.44	2.12	0.28	6.63	[51]	Samples were collected from around Obuasi based on the water demand of the surrounding communities (as a reference to an e-waste worker study).
				Borehole	12	2.73	4.83	0.16	16.6		
				Spring	1	1.2	--	--	--		
				Well	14	0.77	0.8	<0.1	3.04		
Kejetia ASGM community, Talensi District, Upper East Region	2011	d	Groundwater	Borehole	2	11.095	15.5	0.13	22.06	[52]	Samples collected in June and July 2011 from drinking water sources cited by ASGM community members.
				Bottled/sachet	2	0.845	0.43	0.54	1.15		
Nangodi, Talensi-Nabdam District, Upper East Region	2010-2011	d	Groundwater & surface water	Borehole, dug-wells, and dugouts	30	-	-	12.02	65.3	[26]	Samples collected between Nov 2010 to Apr 2011. One site from a borehole (Kalin), 2 from dug wells (Nadpadana, Nakpalig), and 2 from dugouts (Kolikpenge, Debahe).
Kejetia ASGM community, Talensi District, Upper East Region	2011	d	Surface water	Mine pit water	3	135.48	88.9	33.62	197.12	[52]	Samples collected in June and July 2011 from drinking water sources cited by ASGM community members.
				Rain water	1	0.17	-	-	-		
				Stream	9	17.83	8.02	7.45	28.69		
Obuasi, Ashanti Region	2009	d/w	Surface & tap water		48	6600.4	6940.47	5	3460	[29]	Samples collected from Feb - Jun. in the Obuasi mining area, from surface water and tap water.
Obuasi, Ashanti Region	2010	w	Surface water	Process effluent	75	7200	2700	-	-	[53]	Samples collected between Jun. and Sep. 2010 in the morning over 75 days.
				Process effluent	75	7100	2600	-	-		Samples collected between Jun. and Sep. 2010 in the afternoon over 75 days.
				Tailings dam	75	10100	2100	-	-		Samples collected between Jun. and Sep. 2010 in the morning over 75 days.
				Tailings dam	75	9900	2000	-	-		Samples collected between Jun. and Sep. 2010 in the afternoon over 75 days.

ND = Not detectable or below the detection limit.

Table S10. Total arsenic (As) levels in water near Ghanaian small-scale mining sites and reference non-mining sites.

Study Location	Year collected	Dry/wet season	Water type	Water source	Sample Size (n)	Mean As (µg/L)	SD As (µg/L)	Min. As (µg/L)	Max. As (µg/L)	Reference	Notes
Tarkwa, Western Region	2004	d	Surface water	Stream/river	12	18	24	0.5	73	[24]	Drinking water samples taken from around Tarkwa in March 2004.
River Ankobra Basin, Western Region	2002	d	Surface water	Rivers	-	9.61	11.7	0.79	25.56	[16]	Samples collected in February 2002 from five areas on the Ankobra Rive Basin: Ankwaso, Prestea, Dombase, Beppo and Bonsaso, and one area at Anwiaso. Total arsenic.
Obuasi, Ashanti Region	-	w	Surface water	Stream	8	720	-	ND	2200	[54]	Samples from 8 sites at the Pompora Treatment Plant (the old pyrometallurgical pre-treatment plant).
		d			8	1800	-	10	4500		
Tereribe, Anglogold Ashanti-Iduapriem Mine, Tarkwa, Western Region	2010	d	Surface water	Streams	2	< 1	-	< 1	< 1	[49]	Samples collected in March 2010 from Etiheni and Drobo Streams.
Abekoase, Gold Fields Ghana Ltd., Tarkwa, Western Region	2010	d	Surfacewater	River	2	< 1	-	< 1	< 1	[49]	Samples collected in March 2010 from River Essumang.
Huniso, Gold Fields Ghana Ltd., Tarkwa, Western Region	2010	d	Surfacewater	River	1	30	-	-	-	[49]	Samples collected in March 2010 from River Huni.
Obuasi, Ashanti Region	2008	-	Surface water	Stream	1	17.3	--	--	--	[51]	Samples were collected from around Obuasi based on the water demand of the surrounding communities (as a reference to an e-waste worker study).
Reference sites: Non-mining locations											
Across Ghana	2007	w	Groundwater	Boreholes, wells, and some standpipes & trucked water	195	1.93	-	< 0.003	169.5	[55]	Samples collected from boreholes and wells mainly, but also from some standpipes and trucked water during the 2007 rainy season (Jul/Aug) from Regions across Ghana.
Shama, Western Region (Pra); and Elmina (Benya), Cape Coast (Fosu), and Saltpong Beach (Narkwa), Central Region, Gulf of Guinea	-	w	Surface water	Lagoons & estuary	32	0.017	0.028	0.000	0.096	[22]	Samples collected from Pra Estuary, Benya Lagoon, Fosu Lagoon, and Narkwa Lagoon during wet and dry seasons.
Shama, Western Region (Pra); and Elmina (Benya), Cape Coast (Fosu), and Saltpong Beach (Narkwa), Central Region, Gulf of Guinea	-	d	Surface water	Lagoons & estuary	32	0.027	0.048	0.000	0.152		
Accra, Greater Accra Region	2004	d	Groundwater	Standpipe (tap)	1	< 0.1	-	-	-	[24]	Sample collected in March 2004. Sample was not over the detection limit of 0.1 µg/L.

ND = Not detectable or below the detection limit.

Table S10. Total arsenic (As) levels in water near Ghanaian small-scale mining sites and reference non-mining sites.

Study Location	Year collected	Dry/wet season	Water type	Water source	Sample Size (n)	Mean As (μg/L)	SD As (μg/L)	Min. As (μg/L)	Max. As (μg/L)	Reference	Notes
Konongo (Konongo-Odumase), Asante Akim Central District, Ashanti Region	1995-1996	w/d	Groundwater	Boreholes, pipeborne water, spring, well, & underground old mine	64	-	-	ND	12200	[56]	Samples collected from in Konongo and surroundings towns (Odumase, Petriensa, & Obenemase), at 6 sampling sites (water from an underground old mine shaft, boreholes, pipe borne water, spring, and well).
Konongo (Konongo-Odumase), Asante Akim Central District, Ashanti Region	1995-1996	w/d	Surface water	Rivers & tailing dams	64	-	-	30	6900	[56]	Samples collected from in Konongo and surroundings towns (Odumase, Petriensa, & Obenemase), at 10 sampling sites (water from River Owerri, ponds on River Awerekye, and tailing dams).
Accra, Greater Accra Region	2008	-	Groundwater	Tap	10	0.34	0.21	0.1	0.76	[51]	Samples were collected randomly from the environment (tap water) in Accra.

ND = Not detectable or below the detection limit.

Table S11. Total cadmium (Cd) levels in soils near Ghanaian small-scale mining sites and reference non-mining sites.

Study location	Year collected	Dry/wet season	Sample size (n)	Mean Cd (ng/g)	SD Cd (ng/g)	Min. Cd (ng/g)	Max. Cd (ng/g)	Reference	Notes
Wassa West, Western Region	-	-	5	3958.00	1189.70	2870.00	5930	[4]	Samples collected from farms at 5 gold mining towns (Iduapreim, Odumasi, Hemang, Huni Valley, Damang).
Obuasi, Ashanti Region	-	-	36	11.0	3.0	-	-	[57]	Samples collected at 20cm depth from mined, rehabilitaed soil within the Anglogold Ashanti concessions in Obuasi.
			36	10.0	4.0	-	-		Samples collected at 20cm depth from rehabilitated waste dump soil within the Anglogold Ashanti concessions in Obuasi.
			36	20.0	2.0	-	-		Samples collected at 20cm depth from unrehabilitated waste dump soil within the Anglogold Ashanti concessions in Obuasi.
			36	19.0	3.0	-	-		Samples collected at 20cm depth from mined, unrehabilitaed soil within the Anglogold Ashanti concessions in Obuasi.
Abosso Goldfield Ltd.-Daamang, Western Region	-	d	36	30.0	-	-	< 100	[45]	Samples collected at 10cm depth from a variety of soil sites at each mine (Goldfields Ghana Limited - Tarkwa, Abosso Goldfields Limited - Daamang, Anglogold Ashanti - Iduapriem, Bogosu Gold Limited), including topsoil, waste dumps, tailings, and undisturbed vegetation.
Bogosu Gold Ltd., Western Region			20	10.0	-	-	< 100		
Anglogold Ashanti-Iduapriem Mine, Western Region			24	20.0	-	-	< 100		
Goldfields Ghana Ltd.-Tarkwa, Western Region			42	2210	-	-	69100		
Reference sites: Non-mining locations									
Wassa West, Western Region	-	-	3	1710.00	270.00	1420.00	1960	[4]	Samples collected from farms at 3 non-mining towns (Ayanfuri, Wassa Asikuma, and Krofofrom).
Obuasi, Ashanti Region	-	-	36	15.0	2.0	-	-	[57]	Samples collected at 20cm depth from unmined, native soil within the Anglogold Ashanti concessions in Obuasi.
Duabone and Nwowam, Brong-Ahafo Region	-	-	6	0	-	-	-	[45]	3 to 15 random samples of paddy soil samples were collected in inland valley or irrigated paddy fields at a 10cm depth.
Ejura, Kobriti, Biemso (at 2 sites), Odumase, and Nobewam, Ashanti Region			27	210	-	-	-		
Assin Dansame and Assin Akropong, Central Region			9	20	-	-	-		
Abenase, Oda Nkwanta, and Kpong, Eastern Region			36	40	-	-	-		
Sefwi Asafo, Sefwi Anyinabrim, Mempeasem, Aduku Tanoso, Yirase, Simpa Krutu/Anikorkor, and Kobina Anokrom, Western Region			42	10	-	-	-		

Table S12. Total cadmium (Cd) levels in edible plants near Ghanaian small-scale mining sites.

Study location	Year collected	Dry/wet season	Species (common name)	Sample size (n)	Mean Cd (µg/g)	SD Cd (µg/g)	Min. Cd (µg/g)	Max. Cd (µg/g)	Reference	Notes
Wassa West District, Western Region	-	-	Colocasia esculenta (water cocoyam)	8	1.755	1.411	0.34	4.93	[4]	Samples collected from farms at 5 gold mining towns (Iduapreim, Odumasi, Hemang, Huni Valley, Damang) and 3 non-mining towns (Ayanfuri, Wassa Asikuma, Krofofrom).
Tarkwa, Wassa West District, Western Region	-	-	Colocasia esculenta (water cocoyam)	2	182	131	89	274	[12]	Samples collected from 2 mining towns (Nsuta, and Tamso)
Wassa West District, Western Region	-	-	Manihot esculentus (cassava)	8	1.99	0.87	0.76	2.97	[4]	Samples collected from farms at 5 gold mining towns (Iduapreim, Odumasi, Hemang, Huni Valley, Damang) and 3 non-mining towns (Ayanfuri, Wassa Asikuma, Krofofrom).
Wassa West District, Western Region	-	-	Musa paradisiacal (plantain)	8	2.204	1.498	0.12	4.22	[4]	Samples collected from farms at 5 gold mining towns (Iduapreim, Odumasi, Hemang, Huni Valley, Damang) and 3 non-mining towns (Ayanfuri, Wassa Asikuma, Krofofrom).
Wassa West District, Western Region	-	-	Xanthosoma sagittifolium (cocoyam)	8	2.03	1.484	0.54	4.51	[4]	Samples collected from farms at 5 gold mining towns (Iduapreim, Odumasi, Hemang, Huni Valley, Damang) and 3 non-mining towns (Ayanfuri, Wassa Asikuma, Krofofrom).
Tarkwa, Wassa West District, Western Region	-	-	Xanthosoma sagittifolium (cocoyam)	1	43	-	-	-	[12]	Samples collected from Efuantah mining town.

Table S13. Total cadmium (Cd) levels in sediment at reference non-mining sites.

Study location	Year collected	Dry/wet season	Sample size (n)	Mean Cd (ng/g)	SD Cd (ng/g)	Min. Cd (ng/g)	Max. Cd (ng/g)	Reference	Notes
Shama, Western Region (Pra); and Elmina (Benya), Cape Coast (Fosu), and Saltpong Beach (Narkwa), Central Region, Gulf of Guinea	-	w	32	74	70	0	253	[22]	Samples collected from Pra Estuary, Benya Lagoon, Fosu Lagoon, and Narkwa Lagoon during wet and dry season. Non-mining site.
		d		211	373	0	852		
River Subin, Kumasi, Ashanti Region	2005	d/w	10	-	-	280	1420	[48]	Samples were collected bi-monthly form Jan. to Sep. 2005. Non-mining site.

Table S14. Total cadmium (Cd) levels in water near Ghanaian small-scale mining sites and reference non-mining sites.

Study location	Year collected	Dry/wet season	Water type	Water source	Sample size (n)	Mean Cd (µg/L)	SD Cd (µg/L)	Min. Cd (µg/L)	Max. Cd (µg/L)	Reference	Notes
Dumasi, Wassa West District, Western Region	2004-2005	d/w	Groundwater	Boreholes	160	3.5	1.29	2	5	[50]	Samples were collected from 4 boreholes (40 samples at each borehole) between Aug. 2004 and Jun. 2005. Values here are the means from each borehole.
Obuasi, Ashanti Region	2008	-	Groundwater	Tap	8	0.04	0.03	0.01	0.1	[51]	Samples were collected from around Obuasi based on the water demand of the surrounding communities (as a reference to an e-waste worker study).
				Borehole	11	0.04	0.02	< 0.01	0.09		
				Spring	1	0.04	-	-	-		
				Well	14	0.05	0.05	< 0.01	0.19		
Tarkwa, Western Region	2004	d	Groundwater	Borehole	12	0.06	0.05	0.01	0.16	[24]	Drinking water samples taken from around Tarkwa in March 2004.
				Well	6	0.06	0.01	0.04	0.08		
Tereribe, Anglogold Ashanti-Iduapriem Mine, Tarkwa, Western Region	2010	d	Groundwater	Boreholes	4	< 10	-	< 10	< 10	[49]	Samples collected in March 2010 from boreholes.
Mile 7, Anglogold Ashanti-Iduapriem Mine, Tarkwa, Western Region	2010	d	Groundwater	Boreholes	2	120	-	< 10	220	[49]	Samples collected in March 2010 from boreholes.
Abekoase, Gold Fields Ghana Ltd., Tarkwa, Western Region	2010	d	Groundwater	Boreholes	5	140	-	< 10	370	[49]	Samples collected in March 2010 from boreholes.
Huniso, Gold Fields Ghana Ltd., Tarkwa, Western Region	2010	d	Groundwater	Boreholes	2	< 10	-	< 10	< 10	[49]	Samples collected in March 2010 from boreholes.
Kejetia ASGM community, Talensi District, Upper East Region	2011	d	Groundwater	Borehole	2	0.035	0.02	0.02	0.035	[52]	Samples collected in June and July 2011 from drinking water sources cited by ASGM community members.
				Bottled/sachet	2	0.02	0.014	0.01	0.03		
Nangodi, Talensi-Nabdam District, Upper East Region	2010-2011	d	Groundwater & surface water	Borehole, dug-wells, & dugouts	30	-	-	266.5	533	[26]	Samples collected between Nov 2010 to Apr 2011. One site from a borehole (Kalin), 2 from dug wells (Nadpadana, Nakpalig), and 2 from dugouts (Kolikpenge, Debahe).
Kejetia ASGM community, Talensi District, Upper East Region	2011	d	Surface water	Mine pit water	3	0.067	0.025	0.04	0.09	[52]	Samples collected in June and July 2011 from drinking water sources cited by ASGM community members.
				Rain water	1	0.03	-	-	-		
				Stream	9	0.25	0.36	0.03	1.16		
Obuasi, Ashanti Region	2009	d/w	Surface & tap water	-	48	12413	14044.5	45	53700	[29]	Samples collected from Feb - Jun. in the Obuasi mining area.
Obuasi, Ashanti Region	2008	-	Surface water	Stream	1	< 0.01	-	-	-	[51]	Samples were collected from around Obuasi based on the water demand of the surrounding communities (as a reference to an e-waste worker study).
Tarkwa, Western Region	2004	d	Surface water	River/stream	12	0.04	0.05	0.007	0.13	[24]	Drinking water samples taken from around Tarkwa in March 2004.
Tereribe, Anglogold Ashanti-Iduapriem Mine, Tarkwa, Western Region	2010	d	Surface water	Streams	2	< 10	-	< 10	< 10	[49]	Samples collected in March 2010 from Etiheni and Drobo Streams.

Table S14. Total cadmium (Cd) levels in water near Ghanaian small-scale mining sites and reference non-mining sites.

Study location	Year collected	Dry/wet season	Water type	Water source	Sample size (n)	Mean Cd (µg/L)	SD Cd (µg/L)	Min. Cd (µg/L)	Max. Cd (µg/L)	Reference	Notes
Abekoase, Gold Fields Ghana Ltd., Tarkwa, Western Region	2010	d	Surfacewater	River	2	-	-	< 10	270	[49]	Samples collected in March 2010 from River Essumang
Huniso, Gold Fields Ghana Ltd., Tarkwa, Western Region	2010	d	Surfacewater	River	1	< 10	-	-	-	[49]	Samples collected in March 2010 from Huni River.
Reference sites: Non-mining locations											
Across Ghana	2007	w	Groundwater	Boreholes, wells, and some standpipes & trucked water	195	0.025	-	< 0.001	1.755	[55]	Samples collected from boreholes and wells mainly, but also from some standpipes and trucked water during the 2007 rainy season (Jul/Aug) from Regions across Ghana
Shama, Western Region (Pra), and Elmina (Benya), Cape Coast (Fosu), and Saltpong Beach (Narkwa), Central Region, Gulf of Guinea	-	w	Surface water	Estuary & lagoons	16	0.030	0.020	0	0.057	[22]	Samples collected from Pra Estuary, Benya Lagoon, Fosu Lagoon, and Narkwa Lagoon during wet and dry seasons.
		d	Surface water	Estuary & lagoons	16	0.029	0.010	0	0.068		
Accra, Greater Acrra Regoin	2008	-	Groundwater	Tap	9	0.07	0.06	< 0.01	0.19	[51]	Tap water samples randomly collected in Accra.
Tarkwa, Western Region	2004	d	Groundwater	Standpipe (tap)	1	< 0.01	-	-	-	[24]	Samples collected from a standpipe (tap) in Accra in March. No samples over detection level of 0.01 µg/L.

Table S15. Total lead (Pb) levels in soils near Ghanaian small-scale mining sites and reference non-mining sites.

Study location	Year collected	Dry/wet season	Sample size (n)	Mean Pb (ng/g)	SD Pb (ng/g)	Min. Pb (ng/g)	Max. Pb (ng/g)	Reference	Notes
Obuasi, Ashanti Region	-	-	36	52	4	-	-	[57]	Samples collected at 20cm depth from mined, rehabilitaed soil within the Anglogold Ashanti concessions in Obuasi.
	-	-	36	42	8	-	-		Samples collected at 20cm depth from rehabilitated waste dump soil within the Anglogold Ashanti concessions in Obuasi.
	-	-	36	48	3	-	-		Samples collected at 20cm depth from unrehabilitated waste dump soil within the Anglogold Ashanti concessions in Obuasi.
	-	-	36	48	3	-	-		Samples collected at 20cm depth from mined, unrehabilitaed soil within the Anglogold Ashanti concessions in Obuasi.
Abosso Goldfield Ltd.-Daamang, Western Region	-	d	36	5800	-	-	-	[45]	Samples collected at 10cm depth from a variety of soil sites at each mine (Goldfields Ghana Limited - Tarkwa, Abosso Goldfields Limited - Daamang, Anglogold Ashanti - Iduapriem, Bogosu Gold Limited), including topsoil, waste dumps, tailings, and undisturbed vegetation.
Bogosu Gold Ltd., Western Region		d	20	4900	-	-	-		
Anglogold Ashanti-Iduapriem Mine, Western Region		d	24	3800	-	-	-		
Goldfields Ghana Ltd.-Tarkwa, Western Region		d	42	5400	-	-	-		
Reference sites: Non-mining locations									
Obuasi, Ashanti Region	-	-	36	79	20	-	-	[57]	Samples collected at 20cm depth from unmined, native soil within the Anglogold Ashanti concessions in Obuasi.
Duabone and Nwowam, Brong-Ahafo Region	-	-	6	7200	-	-	-	[45]	3 to 15 random samples of paddy soil samples were collected in inland valley or irrigated paddy fields at a 10cm depth.
Ejura, Kobriti, Biemso (at 2 sites), Odumase, and Nobewam, Ashanti Region		-	27	7800	-	-	-		
Assin Dansame and Assin Akropong, Central Region		-	9	11000	-	-	-		
Abenase, Oda Nkwanta, and Kpong, Eastern Region		-	36	9500	-	-	-		
Sefwi Asafo, Sefwi Anyinabrim, Mempeasem, Aduku Tanoso, Yirase, Simpa Krutu/Anikorkor, and Kobina Anokrom, Western Region		-	42	6700	-	-	-		

Table S16. Total lead (Pb) levels in edible plants near Ghanaian small-scale mining sites and reference non-mining sites.

Study location	Year collected	Dry/wet season	Species (common name/component)	Sample size (n)	Mean Pb (μg/g)	SD Pb (μg/g)	Min. Pb (μg/g)	Max. Pb (μg/g)	Reference	Notes
Obuasi, Obuasi Municipal District, Ashanti Region	1991	-	Citrus sinesis (orange flesh)	60	1.3	-	0.8	1.8	[58]	Fruit was harvested from 6 farms, and 10 fruits were harvested from each tree.
			Citrus sinesis (orange peel)	60	0.8	-	0.2	1.5		
			Persea americana (avocado flesh)	60	1.1	-	0.3	1.9		
			Persea americana (avocado peel)	60	2.2	-	1.9	2.5		
Reference sites: Non-mining locations										
Konongo (Konongo-Odumase), Asante Akim Central District, Ashanti Region	1991	-	Citrus sinesis (orange flesh)	60	1.9	-	0.6	3.2	[58]	Fruit was harvested from 6 farms, and 10 fruits were harvested from each tree.
			Citrus sinesis (orange peel)	60	1.5	-	1	1.9		
			Persea americana (avocado flesh)	60	0.7	-	0.3	1.1		
			Persea americana (avocado peel)	60	1.3	-	1	1.5		
Kumasi, Kumasi Metropolitan District, Ashanti Region	1991	-	Citrus sinesis (orange flesh)	60	1.3	-	0.4	2.2	[58]	Fruit was harvested from 6 farms, and 10 fruits were harvested from each tree. Kumasi data is control.
			Citrus sinesis (orange peel)	60	1.2	-	0.8	1.6		
			Persea americana (avocado flesh)	60	0.9	-	0.6	1.2		
			Persea americana (avocado peel)	60	1.2	-	0.4	2.4		

Table S17. Total lead (Pb) levels in sediment near Ghanaian small-scale mining sites and reference non-mining sites.

Study location	Year collected	Dry/wet season	Sample size (n)	Mean Pb (ng/g)	SD Pb (ng/g)	Min. Pb (ng/g)	Max. Pb (ng/g)	Reference	Notes
Mining area, River Pra Basin, Southwestern	2002-2003	d	14	ND	ND	ND	ND	[47]	14 sampling sites along the River Pra (2 downstream--1 past ASGM--and 12 upstream)
		w	14	243	635	14	2441		
Non-mining area, River Pra Basin, Southwestern	2002-2003	d	7	ND	ND	ND	ND	[47]	7 sites from non-mining areas along the River Pra (5 are from downstream and 2 upstream)
		w	7	105	86	12	238		

ND = Not detectable or below the detection limit.

Table S18. Total lead (Pb) levels in water near Ghanaian small-scale mining sites and reference non-mining sites.

Study location	Year collected	Dry/wet season	Water type	Water source	Sample size (n)	Mean Pb (µg/L)	SD Pb (µg/L)	Min. Pb (µg/L)	Max. Pb (µg/L)	Reference	Notes
Tarkwa, Western Region	2004	d	Groundwater	Borehole	12	0.16	0.1	0.01	0.32	[24]	Drinking water samples taken from around Tarkwa in March 2004.
Tarkwa, Western Region	2004	d	Groundwater	Well	6	7.76	17.2	0.3	42.9	[24]	Drinking water samples taken from around Tarkwa in March 2004.
Obuasi, Ashanti Region	2008	-	Groundwater	Tap	8	0.23	0.19	0.05	0.57	[50]	Samples were collected from around Obuasi based on the water demand of the surrounding communities (as a reference to an e-waste worker study).
				Borehole	12	1.86	5.9	0.01	20.6		
				Spring	1	0.11	-	-	-		
				Well	16	0.2	0.13	0.05	0.44		
Tereribe, Anglogold Ashanti-Iduapriem Mine, Tarkwa, Western Region	2010	d	Groundwater	Boreholes	3	< 10	-	< 10	< 10	[48]	Samples collected in March 2010 from boreholes.
Mile 7, Anglogold Ashanti-Iduapriem Mine, Tarkwa, Western Region	2010	d	Groundwater	Boreholes	2	< 10	-	< 10	< 10	[48]	Samples collected in March 2010 from boreholes.
Abekoase, Gold Fields Ghana Ltd., Tarkwa, Western Region	2010	d	Groundwater	Boreholes	5	< 10	-	< 10	< 10	[48]	Samples collected in March 2010 from boreholes.
Huniso, Gold Fields Ghana Ltd., Tarkwa, Western Region	2010	d	Groundwater	Boreholes	2	< 10	-	< 10	< 10	[48]	Samples collected in March 2010 from boreholes.
Dumasi, Wassa West District, Western Region	2004-2005	d/w	Groundwater	Boreholes	160	6.5	1.29	5	8	[49]	Samples were collected from 4 boreholes (40 samples at each borehole) between Aug. 2004 and Jun. 2005. Values here are the means from each borehole.
Kejetia ASGM community, Talensi District, Upper East Region	2011	d	Groundwater	Borehole	2	0.335	0.106	0.26	0.41	[51]	Samples collected in June and July 2011 from drinking water sources cited by ASGM community members.
				Bottled/sachet	2	0.145	0.0354	0.12	0.17		
Obuasi, Ashanti Region	2009	d/w	Surface & tap water	-	48	1580.21	2933.434	1	15200	[29]	Samples collected from Feb - Jun. in the Obuasi mining area.
Obuasi, Ashanti Region	2010	w	Surface water	Process effluent	75	200	200	-	-	[52]	Samples collected between Jun. and Sep. 2010 in the morning.
				Profess effluent	75	200	300	-	-		Samples collected between Jun. and Sep. 2010 in the afternoon.
				Tailings dam	75	100	100	-	-		Samples collected between Jun. and Sep. 2010 in the morning.
				Tailings dam	75	200	200	-	-		Samples collected between Jun. and Sep. 2010 in the afternoon.
Tarkwa, Western Region	2004	d	Surface water	River/stream	12	0.85	1.06	0.02	3.71	[24]	Drinking water samples taken from around Tarkwa in March 2004.
Obuasi, Ashanti Region	2008	-	Surface water	Stream	1	0.01	-	-	-	[50]	Samples were collected from around Obuasi based on the water demand of the surrounding communities (as a reference to an e-waste worker study).
Tereribe, Anglogold Ashanti-Iduapriem Mine, Tarkwa, Western Region	2010	d	Surface water	Streams	2	< 10	-	< 10	< 10	[48]	Samples collected in March 2010 from Etiheni and Drobo Streams.

ND = Not detectable or below the detection limit.

Table S18. Total lead (Pb) levels in water near Ghanaian small-scale mining sites and reference non-mining sites.

Study location	Year collected	Dry/wet season	Water type	Water source	Sample size (n)	Mean Pb (µg/L)	SD Pb (µg/L)	Min. Pb (µg/L)	Max. Pb (µg/L)	Reference	Notes
Abekoase, Gold Fields Ghana Ltd., Tarkwa, Western Region	2010	d	Surfacewater	River	2	< 10	-	< 10	< 10	[48]	Samples collected in March 2010 from River Essumang.
Huniso, Gold Fields Ghana Ltd., Tarkwa, Western Region	2010	d	Surfacewater	River	1	< 10	-	< 10	< 10	[48]	Samples collected in March 2010 from Huni River.
Obuasi, Ashanti Region	-	d	Surface water	Stream	8	ND	-	-	-	[54]	Samples from 8 sites at the Pompora Treatment Plant (the old pyrometallurgical pre-treatment plant). Samples were all below detection limit (10 µg/L).
		w			8	ND	-	-	-		
Kejetia ASGM community, Talensi District, Upper East Region	2011	d	Surface water	Mine pit water	3	2.65	2.44	0.54	5.33	[52]	Samples collected in June and July 2011 from drinking water sources cited by ASGM community members.
				Rain water	1	1.51	-	-	-		
				Stream	9	13.97	29.7	1.48	92.7		
Reference sites: Non-mining locations											
Across Ghana	2007	w	Groundwater	Boreholes, wells, and some standpipes & trucked water	195	1.526	-	< 0.006	34.94	[55]	Samples collected from boreholes and wells mainly, but also from some standpipes and trucked water during the 2007 rainy season (Jul/Aug) from Regions across Ghana.
Accra, Greater Accra Region	2004	d	Groundwater	Tap	1	0.05	-	-	-	[24]	Tap in Accra, as control.
Accra, Greater Accra Region	2008	-	Groundwater	Tap	10	0.4	0.34	0.09	1.23	[51]	Samples were collected randombly from the environment (tap water) in Accra.

ND = Not detectable or below the detection limit.

Table S19. Guidelines and standards for mercury (Hg) in ecological media. Data are reviewed by the number of sampling sites exceeding guideline values for total, mining (ASGM and/or LSGM), and non-mining sampling sites.

	Mercury (Hg)				
	Standard		*n* sites (%) > std.	*n* sampling sites*	
Soil	0.1 μg/g THg	US EPA [59, 60]	26 (81.3)	32	Total
			22 (88.0)	25	Mining
			4 (57.1)	7	Non-mining
Plants	0.5 μg/g MeHg	FAO/WHO [61]	4 (28.6)	14	Total
			4 (28.6)	14	Mining
			0 (0)	0	Non-mining
Sediment	0.18 μg/g THg	US EPA [62]	17 (51.5)	33	Total
			16 (59.3)	27	Mining
			1 (16.7)	6	Non-mining
Water	6 μg/L Inorg. Hg	WHO [63]	7 (18.9)	37	Total
			7 (23.3)	30	Mining
			0 (0)	7	Non-mining
Fish	0.3 μg/g MeHg	US EPA [64]	7 (6.9)	101	Total
			4 (9.8)	41	Mining
			3 (5.0)	60	Non-mining
	0.5 μg/g MeHg	FAO/WHO [61]	3 (3.0)	101	Total
			2 (4.9)	41	Mining
			1 (1.7)	60	Non-mining
Tailings	0.1 μg/g THg	US EPA [59, 60]	8 (66.7)	12	Total
			8 (66.7)	12	Mining
			0 (0)	0	Non-mining

*Sampling sites are sites sampled at a given location and time point, that include data on mean metals concentrations.

Table S20. Guidelines and standards for arsenic (As) in ecological media. Data are reviewed by the number of sampling sites exceeding guideline values for total, mining (ASGM and/or LSGM), and non-mining sampling sites.

	Arsenic (As)				
	Standard		*n* sites (%) > std.	*n* sampling sites*	
Soil	0.4 μg/g	US EPA [60]	16 (94.1)	17	Total
			6 (100)	6	Mining
			10 (90.9)	11	Non-mining
Plants	0.1 μg/g	FAO/WHO [61]	10 (100)	10	Total
			10 (100)	10	Mining
			0 (0)	0	Non-mining
Sediment	9.8 μg/g	US EPA [62]	1 (14.3)	7	Total
			1 (33.3)	3	Mining
			0 (0)	4	Non-mining
Water	10 μg/L	WHO, GSB/GWC [63,65]	18 (52.9)	34	Total
			18 (62.1)	29	Mining
			0 (0)	5	Non-mining

*Sampling sites are sites sampled at a given location and time point, that include data on mean metals concentrations.

Table S21. Guidelines and standards for cadmium (Cd) in ecological media. Data are reviewed by the number of sampling sites exceeding guideline values for total, mining (ASGM and/or LSGM), and non-mining sampling sites.

	Cadmium (Cd)				
	Standard		*n* sites (%) > std.	*n* sampling sites*	
Soil	78 µg/g	US EPA [60]	0 (0)	11	Total
			0 (0)	4	Mining
			0 (0)	7	Non-mining
Plants	0.1 µg/g	FAO/WHO [61]	6 (100)	6	Total
			6 (100)	6	Mining
			0 (0)	0	Non-mining
Sediment	0.99 µg/g	US EPA [62]	0 (0)	2	Total
			0 (0)	0	Mining
			0 (0)	2	Non-mining
Water	3.0 µg/L	WHO, GSB/GWC [63,65]	4 (15.4)	26	Total
			4 (19.0)	21	Mining
			0 (0)	5	Non-mining

*Sampling sites are sites sampled at a given location and time point, that include data on mean metals concentrations.

Table S22. Guidelines and standards for lead (Pb) in ecological media. Data are reviewed by the number of sampling sites exceeding guideline values for total, mining (ASGM and/or LSGM), and non-mining sampling sites.

	Lead (Pb)				
	Standard		*n* sites (%) > std.	*n* sampling sites*	
Soil	400 µg/g	US EPA [60]	0 (0)	8	Total
			0 (0)	8	Mining
			0 (0)	6	Non-mining
Plants	0.1 µg/g	FAO/WHO [61]	12 (100)	12	Total
			4 (100)	4	Mining
			8 (100)	8	Non-mining
Sediment	35.8 µg/g	US EPA [62]	0 (0)	4	Total
			0 (0)	2	Mining
			0 (0)	2	Non-mining
Water	10 µg/L	WHO, GSB/GWC [63,66, 67]	6 (19.4)	31	Total
			6 (21.4)	28	Mining
			0 (0)	3	Non-mining

*Sampling sites are sites sampled at a given location and time point, that include data on mean metals concentrations.

References

1. Bonzongo, J.J.; Donkor, A.K.; Nartey, V.K.; Lacerda, L.D. Mercury pollution in Ghana: A case study of environmental impacts of artisanal gold mining in Sub-Saharan Africa. In *Environmental Geochemistry in Tropical and Subtropical Environments*; Drude de Lacerda, L., Santelli, R.E., Duursma, E.K., Abrão, J.J., Eds.; Springer Berlin Heidelberg: Heidelberg, Berlin, Germany, 2004; pp. 135–156.
2. Adjorlolo-Gasokpoh, A.; Golow, A.A.; Kambo-Dorsa, J. Mercury in the surface soil and cassava, *Manihot esculenta* (flesh, leaves and peel) near goldmines at Bogoso and Prestea, Ghana. *Bull. Environ. Contam. Toxicol.* **2012**, *89*, 1106–1110.
3. Tetteh, S.; Golow, A.A.; Essumang, D.K.; Zugle, R. Levels of mercury, cadmium, and zinc in the topsoil of some selected towns in the Wassa West District of the Western Region of Ghana. *Soil Sediment Contam*. **2010**, *19*, 635–643.
4. Ahiamadjie, H.; Serfor-Armah, Y.; Tandoh, J.B.; Gyampo, O.; Ofosu, F.G.; Dampare, S.B.; Adotey, D.K.; Nyarko, B.J.B. Evaluation of trace elements contents in staple foodstuffs from the gold mining areas in southwestern part of Ghana using neutron activation analysis. *J. Radioanal. Nucl. Chem.* **2011**, *288*, 653–661.
5. Azanu, D. *Sorption of Inorganic Mercury on Soils from Ankobra Basin in the South-Western Part of Ghana*; Kwame Nkrumah University of Science and Technology: Kumasi, Ghana, 2010.
6. Quarshie, E.; Nyarko, B.J.; Serfor-Armah, Y. Studies of levels of some toxic elements in soil and tailings from Bibiani mining area of Ghana. *Res. J. Environ. Earth Sci.* **2011**, *3*, 512–520.
7. Amonoo-Neizer, E.H.; Nyamah, D.; Bakiamoh, S.B. Mercury and arsenic pollution in soil and biological samples around the mining town of Obuasi, Ghana. *Water Air Soil Pollut.* **1996**, *91*, 363–373.
8. Donkor, A.K.; Bonzongo, J.C.; Nartey, V.K.; Adotey, D.K. Mercury in different environmental compartments of the Pra River Basin, Ghana. *Sci. Total Environ.* **2006**, *368*, 164–176.
9. Oppong, S.O.B.; Voegborlo, R.B.; Agorku, S.E.; Adimado, A.A. Total mercury in fish, sediments and soil from the River Pra Basin, southwestern Ghana. *Bull. Environ. Contam. Toxicol.* **2010**, *85*, 324–329.
10. Rajaee, M.; Long, R.N.; Renne, E.P.; Basu, N. Mercury concentrations and spatial distribution in a Ghanaian small-scale gold mining community. *Int. J. Environ. Res. Public Health* **2015**, under review.
11. Samlafo, B.V.; Sarah, L.A.; Quarshie, E.; Serfor-Armah, Y. Arsenic and mercury levels in earthenware clays in Otsew in Gomoa West District of Central Region of Ghana using Instrumental Neutron Activation Analysis. *Res. J. Environ. Earth Sci.* **2011**, *3*, 541–545.
12. Essumang, D.K.; Dodoo, D.K.; Obiri, S.; Yaney, J.Y. Arsenic, cadmium, and mercury in cocoyam (*Xanthosoma sagititolium*) and watercocoyam (*Colocasia esculenta*) in Tarkwa a mining community. *Bull. Environ. Contam. Toxicol.* **2007**, *79*, 377–379.
13. Babut, M.; Sekyi, R.; Rambaud, A.; Potin-Gautier, M.; Tellier, S.; Bannerman, W.; Beinhoff, C. Improving the environmental management of small-scale gold mining in Ghana: A case study of Dumasi. *J. Clean. Prod.* **2003**, *11*, 215–221.

14. Serfor-Armah, Y.; Nyarko, B.J.B.; Adotey, D.K.; Adomako, D.; Akaho, E.H.K. The impact of small-scale mining activities on the levels of mercury in the environment: The case of Prestea and its environs. *J. Radioanal. Nucl. Chem.* **2004**, *262*, 685–690.
15. Boamponsem, L.K.; Adam, J.I.; Dampare, S.B.; Owusu-Ansah, F.; Addae, G. Heavy metals level in streams of Tarkwa gold mining area of Ghana. *J. Chem. Pharm. Res.* **2010**, *2*, 504–527.
16. Bannerman, W.; Potin-Gautier, M.; Amouroux, D.; Tellier, S.; Rambaud, A.; Babut, M.; Adimado, A.A.; Beinhoff, C. Mercury and arsenic in the gold mining regions of the Ankobra River basin in Ghana. *Journal de Physique IV* **2003**, *107*, 107–110.
17. Akabzaa, T.M.; Yidana, S.M. Evaluation of sources and options for possible clean up of anthropogenic mercury contamination in the Ankobra River Basin in South Western Ghana. *J. Environ. Prot. (Irvine Calif).* **2011**, *2*, 1295–1302.
18. Nartey, V.K.; Doamekpor, L.K.; Sarpong-Kumankuma, S.; Akabzaa, T.M.; Nyame, F.K.; Kutor, J.; Adotey, D.K. Mercury pollution studies of some rivers draining the Bibiani-Anwiaso-Bekwai mining community of south western Ghana. *Res. J. Environ. Earth Sci.* **2011**, *3*, 375–384.
19. Golow, A.A.; Mingle, L.C. Mercury in river water and sediments in some rivers near Dunkwa-On-Offin, an Alluvial Goldmine, Ghana. *Bull. Environ. Contam. Toxicol.* **2003**, *70*, 379–384.
20. Dwumah-Akoanah, S. Studies on levels of mercury, cadmium and zinc in fish and sediments from River Offin in Ghana; Kwame Nkrumah University of Science and Technology, Kumasi, Ghana, 2008.
21. Nartey, V.; Klake, R.K.; Hayford, E.; Doamekpor, L.K.; Appoh, R. Assessment of mercury pollution in rivers and streams around artisanal gold mining areas of the Birim North District of Ghana. *J. Environ. Prot. (Irvine, Calif).* **2011**, *2*, 1227–1239.
22. Adokoh, C.K.; Obodai, E.A.; Essumang, D.K.; Serfor-Armah, Y.; Nyarko, B.J.B.; Asabere-Ameyaw, A. Statistical evaluation of environmental contamination, distribution and source assessment of heavy metals (aluminum, arsenic, cadmium, and mercury) in some lagoons and an estuary along the coastal belt of Ghana. *Arch. Environ. Contam. Toxicol.* **2011**, *61*, 389–400.
23. Madkour, H.A.; Obirikorang, K.A.; Amisah, S.; Otchere, F.A.; Adjei-Boateng, D. Relationship between heavy metal concentrations in bottom sediments and the clam, Galatea Paradoxa (born 1778) from the Volta Estuary, Ghana. *J. Environ. Prot. (Irvine Calif.)*. **2011**, *2*, 720–728.
24. Asante, K.A.; Agusa, T.; Subramanian, A.; Ansa-Asare, O.D.; Biney, C.A.; Tanabe, S.; Ansong, K. Contamination status of arsenic and other trace elements in drinking water and residents from Tarkwa, a historic mining township in Ghana. *Chemosphere* **2007**, *66*, 1513–1522.
25. Obiri, S.; Dodoo, D.K.; Armah, F.A.; Essumang, D.K.; Cobbina, S.J. Evaluation of lead and mercury neurotoxic health risk by resident children in the Obuasi Municipality, Ghana. *Environ. Toxicol. Pharmacol*. **2010**, *29*, 209–212.
26. Cobbina, S.J.; Dagben, J.Z.; Obiri, S.; Tom-Dery, D. Assessment of non-cancerous health risk from exposure to Hg, As and Cd by resident children and adults in Nangodi in the Upper East Region, Ghana. *Water Qual. Expo. Heal.* **2011**, *3*, 225–323.

27. Cobbina, S.J.; Nkuah, D.; Tom-dery, D.; Obiri, S. Non-cancer risk assessment from exposure to mercury (Hg), cadmium (Cd), arsenic (As), copper (Cu) and lead (Pb) in boreholes and surface water in Tinga , in the Bole-Bamboi District, Ghana. *Glob. J. Toxicol. Signal Transduct.* **2013**, *1*, 49–56.
28. Ofosu-Asiedu, L.; Cobbina, S.J.; Obiri, S. Non-cancer human health risk assessment from exposure to cadmium, copper, lead and mercury in surface water and ground water in Konongo-Odumasi Municipality, Ghana. *J. Environ. Chem. Ecotoxicol.* **2013**, *5*, 106–112.
29. Armah, F.A.; Obiri, S.; Yawson, D.O.; Onumah, E.E.; Yengoh, G.T.; Afrifa, E.K.A.; Odoi, J.O. Anthropogenic sources and environmentally relevant concentrations of heavy metals in surface water of a mining district in Ghana: a multivariate statistical approach. *J. Environ. Sci. Health. A. Tox. Hazard. Subst. Environ. Eng.* **2010**, *45*, 1804–1813.
30. Kortatsi, B.K.; Tay, C.K.; Anornu, G.; Hayford, E.; Dartey, G.A. Hydrogeochemical evaluation of groundwater in the lower Offin Basin, Ghana. *Environ. Geol.* **2008**, *53*, 1651–1662.
31. Kwaansa-Ansah, E.E.; Agorku, S.E.; Nriagu, J.O. Levels of total mercury in different fish species and sediments from the Upper Volta Basin at Yeji in Ghana. *Bull. Environ. Contam. Toxicol.* **2011**, *86*, 406–409.
32. Laar, C.; Fianko, J.R.; Akiti, T.T.; Osae, S.; Brimah, A.K. Determination of heavy metals in the Black-Chin Tilapia from the Sakumo Lagoon, Ghana. *Res. J. Environ. Earth Sci.* **2011**, *3*, 8–13.
33. Agorku, E.S.; Voegborlo, R.B.; Adimado, A.A. Total mercury levels in nine species of freshwater fish from two hydroelectric reservoirs and a crater lake in Ghana. *Environ. Monit. Assess.* **2009**, *153*, 383–389.
34. Voegborlo, R.B.; Adimado, A.A.; Ephraim, J.H. Total mercury distribution in different tissues of frigate tuna (*Auxis thazard* thazard) from the Atlantic Coastal Waters of Ghana, Gulf of Guinea. *Environ. Monit. Assess.* **2007**, *132*, 503–508.
35. Voegborlo, R.B.; Baah, D.A.; Kwaansa-Anah, E.; Adimado, A.A.; Ephraim, J.H. Mercury concentrations in fish species from the Gulf of Guinea, Ghana. *Bull. Environ. Contam. Toxicol.* **2004**, *73*, 1057–1064.
36. Voegborlo, R.B.; Matsuyama, A.; Akagi, H.; Adimado, A.A.; Ephraim, J.H. Total mercury and methylmercury accumulation in the muscle tissue of frigate (*Auxis thazard* thazard) and yellow fin (*Thunnus albacares*) tuna from the Gulf of Guinea, Ghana. *Bull. Environ. Contam. Toxicol.* **2006**, *76*, 840–847.
37. Voegborlo, R.B.; Akagi, H. Determination of mercury in fish by cold vapour atomic absorption spectrometry using an automatic mercury analyzer. *Food Chem.* **2007**, *100*, 853–858.
38. Joiris, C.R.; Azokwu, M.I.; Otchere, F.A.; Ali, I.B. Mercury in the bivalve *Anadara* (Senilia) *senilis* from Ghana and Nigeria. *Sci. Total Environ.* **1998**, *224*, 181–188.
39. Joiris, C.R.; Holsbeek, L.; Otchere, F.A. Mercury in the Bivalves *Crassostrea tulipa* and *Perna perna* from Ghana. *Mar. Pollut. Bull.* **2000**, *40*, 457–460.
40. Froese, R.; Pauly, D. FishBase www.fishbase.org (accessed Jul 1, 2014).

41. Boamponsem, L.K.; Adam, J.I.; Dampare, S.B.; Nyarko, B.J.B.; Essumang, D.K. Assessment of atmospheric heavy metal deposition in the Tarkwa gold mining area of Ghana using epiphytic lichens. *Nucl. Instruments Methods Phys. Res. Sect. B Interact with Mater. Atoms.* **2010**, *268*, 1492–1501.
42. Nyarko, B.J.B.; Dampare, S.B.; Serfor-Armah, Y.; Osae, S.; Adotey, D.K.; Adomako, D. Biomonitoring in the forest zone of Ghana: The primary results obtained using neutron activation analyses and lichens. *Int. J. Environ. Pollut.* **2008**, *32*, 467–476.
43. Serfor-Armah, Y.; Nyarko, B.J.B.; Osae, E.K.; Carboo, D.; Seku, F. Elemental analysis of some green and brown seaweeds from the coastal belt of Ghana. *J. Radioanal. Nucl. Chem.* **1999**, *242*, 193–197.
44. Serfor-Armah, Y.; Nyarko, B.J.B.; Osae, E.K.; Carboo, D.; Anim-Sampong, S.; Seku, F. Rhodophyta seaweed species as bioindicators for monitoring toxic element pollutants in the marine ecosystem of Ghana. *Water Air Soil Pollut.* **2001**, *127*, 243–253.
45. Adomako, E.E.; Deacon, C.; Meharg, A.A. Variations in concentrations of arsenic and other potentially toxic elements in mine and paddy soils and irrigation waters from Southern Ghana. *Water Qual. Expo. Heal.* **2010**, *2*, 115–124.
46. Dampare, S.B.; Ameyaw, Y.; Adotey, D.K.; Osae, S.; Nyarko, B.J.B.; Adomako, D. Seasonal trend of potentially toxic trace elements in soils supporting medicinal plants in the Eastern Region of Ghana. *Wate Air Soil Pollut.* **2005**, *169*, 185–206.
47. Donkor, A.K.; Bonzongo, J.; Nartey, V.; Adotey, D.K. Heavy metals in sediments of the gold mining impacted Pra River Basin , Ghana , West Africa. *Soil Sediment Contam.* **2005**, *14*, 479–503.
48. Adomako, D.; Nyarko, B.J.B.; Dampare, S.B.; Serfor-Armah, Y.; Osae, S.; Fianko, J.R.; Akaho, E.H.K. Determination of toxic elements in waters and sediments from River Subin in the Ashanti Region of Ghana. *Environ. Monit. Assess.* **2008**, *141*, 165–175.
49. Kusimi, J.M.; Kusimi, B.A. The hydrochemistry of water resources in selected mining communities in Tarkwa. *J. Geochemical Explor.* **2012**, *112*, 252–261.
50. Obiri, S. Determination of heavy metals in water from boreholes in Dumasi in the Wassa West District of western region of Republic of Ghana. *Environ. Monit. Assess.* **2007**, *130*, 455–463.
51. Asante, K.A.; Agusa, T.; Biney, C.A.; Agyekum, W.A.; Bello, M.; Otsuka, M.; Itai, T.; Takahashi, S.; Tanabe, S. Multi-trace element levels and arsenic speciation in urine of e-waste recycling workers from Agbogbloshie, Accra in Ghana. *Sci. Total Environ.* **2012**, *424*, 63–73.
52. Long, R.; Renne, E.; Robins, T.; Wilson, M.; Pelig-ba, K.; Rajaee, M.; Yee, A.; Koomson, E.; Sharp, C.; Lu, J.; Basu, N. Water values in a Ghanaian small-scale gold mining community. *Hum. Organ.* **2013**, *72*, 199–210.
53. Acheampong, M.A.; Paksirajan, K.; Lens, P.N.L. Assessment of the effluent quality from a gold mining industry in Ghana. *Environ. Sci. Pollut. Res. Int.* **2013**, *20*, 3799–3811.
54. Foli, G.; Nude, P.M. Concentration levels of some inorganic contaminants in streams and sediments in areas of pyrometallurgical and hydrometallurgical activities at the Obuasi gold mine, Ghana. *Environ. Earth Sci.* **2011**, *65*, 753–763.

55. Rossiter, H.M.A.; Owusu, P.A.; Awuah, E.; Macdonald, A.M.; Schäfer, A.I. Chemical drinking water quality in Ghana: Water costs and scope for advanced treatment. *Sci. Total Environ.* **2010**, *408*, 2378–2386.
56. Boadu, M.; Osae, E.K.; Golow, A.A.; Nyarko, B.J.B. Determination of arsenic in some water bodies, untreated ore and tailing samples at Konongo in the Ashanti region of Ghana and its surrounding towns and villages by instrumental neutron activation analysis. *J. Radioanal. Nucl. Chem.* **2001**, *249*, 581–585.
57. Dorgbetor, W.H.K. Evaluation of quality of some rehabilitated mined soils within the AngloGold-Ashanti Concession in Ghana. *Int. J. Geosci.* **2012**, *3*, 50–61.
58. Golow, A.A.; Laryea, J.N. Levels of iron, silver, zinc, and lead in oranges and avocados from two gold-rich towns compared with levels in an adjacent gold-deficient town. *Bull. Environ. Contam. Toxicol.* **1994**, *53*, 332–336.
59. Buchman, M.F. *NOAA Screening Quick Reference Tables*; NOAA: Seattle, WA, USA, 2008; pp. 1–34.
60. U.S. Environmental Protection Agency (EPA) Generic Soil Screening Levels 2011, 1–11.
61. Food and Agriculture Organization (FAO); World Health Organization (WHO) *Codex General Standard for Contaminants and Toxins in Food and Feed*; WHO: Geneva, Switzerland, 2013; pp. 1–48.
62. U.S. Environmental Protection Agency (EPA). *Freshwater Sediment Screening Benchmarks*; US EPA: Washington, DC, USA, 2006; pp. 1–6.
63. World Health Organization (WHO) *Guidelines for Drinking-Water Quality: Fourth Edition*; WHO: Geneva, Switzerland, 2011.
64. U.S. Environmental Protection Agency (EPA) *Water Quality Criterion for the Protection of Human Health: Methylmercury*; US EPA: Washington, DC, USA, 2001.
65. Ghana Environmental Protection Agency (EPA) Environmental Quality Standards--Mining Sector 2010.
66. Kusimi, J.M.; Kusimi, B.A. The hydrochemistry of water resources in selected mining communities in Tarkwa. *J. Geochemical Explor.* **2012**, *112*, 252–261.
67. Obiri, S. Determination of heavy metals in water from boreholes in Dumasi in the Wassa West District of western region of Republic of Ghana. *Environ. Monit. Assess.* **2007**, *130*, 455–463.
